# Comparative kinetic analysis of PNA and γ-miniPEG-PNA hybridization with RNA by surface plasmon resonance

**DOI:** 10.1039/d6ob01345g

**Published:** 2026-10-16

**Authors:** Birgit Meindl, Agnes Lenz, Matthias Schittmayer, Hannes Mikula, Brigitte Holzer

**Affiliations:** a TU Wien, Institute of Applied Synthetic Chemistry Getreidemarkt 9 1060 Wien Austria brigitte.holzer@tuwien.ac.at; b TU Wien, Institute of Chemical Technology and Analytics Getreidemarkt 9 1060 Wien Austria

## Abstract

Nucleic acid analogs that bind RNA through sequence-specific base pairing have emerged as important tools for modulating RNA function. Peptide nucleic acids (PNAs), featuring a neutral *N*-(2-aminoethyl)-glycine backbone, exhibit high affinity, sequence selectivity and enzymatic stability. Their non-phosphodiester backbone prevents RNase H recruitment, favoring steric and recognition-based mechanisms. However, conventional PNAs are limited by poor aqueous solubility, aggregation, non-specific interactions and insufficient pharmacokinetic properties. This limitation can be addressed by incorporating γ-miniPEG (mp) side chains, which improve solubility, reduce aggregation and preorganize the backbone while retaining favorable binding characteristics. Because biological activity depends on target engagement and residence time, understanding PNA/RNA hybridization kinetics is essential, in addition to cellular uptake, localization and target accessibility. A quantitative understanding of hybridization kinetics is therefore critical for candidate selection. Yet, direct comparisons of γ-miniPEG-modified PNAs with RNA remain scarce; to date, such studies have been limited to mp-PNA/DNA duplexes. Here, we systematically compare the hybridization of RNA, PNA and mp-PNA toward complementary RNA using surface plasmon resonance (SPR) spectroscopy, which enables real-time analysis of association and dissociation kinetics at constant temperature. Unlike thermal melting analysis, SPR resolves individual kinetic steps and provides insights into duplex stability under defined conditions. Our results reveal that dissociation kinetics depend strongly on strand length, whereas association is governed primarily by backbone chemistry. Unmodified PNA associates 5–8-fold faster than RNA while maintaining strong affinity. These findings establish a quantitative kinetic framework for understanding how backbone architecture and oligomer length shape PNA/RNA hybridization dynamics, supporting kinetic characterization as a complement to equilibrium measurements in the evaluation of PNA designs.

## Abstract 2

We compare PNA, γ-miniPEG-PNA, and RNA hybridization to complementary RNA by SPR, revealing dynamic antisense recognition beyond conventional stability measurements.

## Graphical abstract

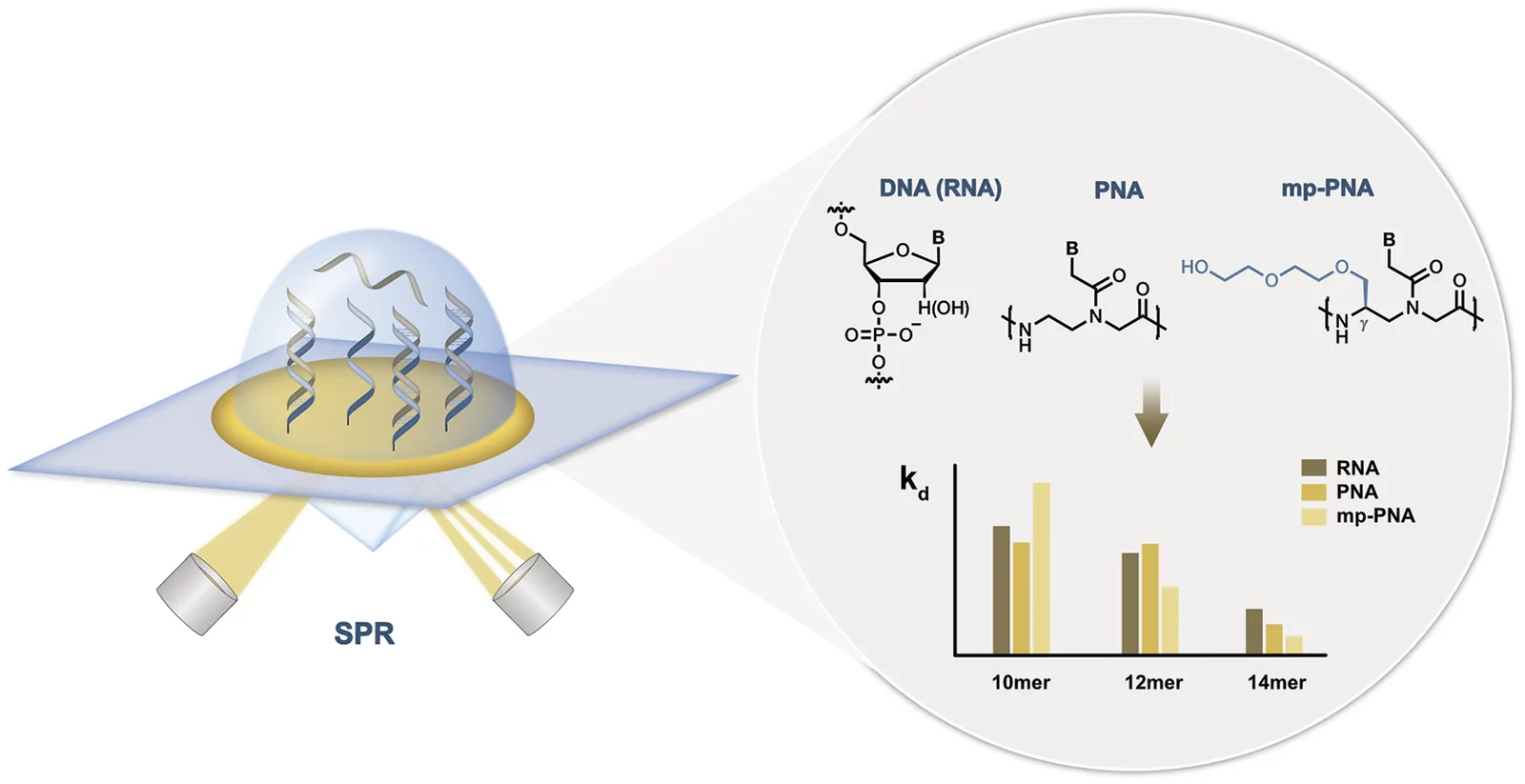

## Introduction

RNA is a central target in molecular biology and plays a key role in both research and therapeutic applications. Peptide nucleic acids (PNAs), DNA and RNA mimics with a neutral *N*-(2-aminoethyl)glycine backbone (Fig. 1), bind complementary nucleic acids with high affinity and exceptional enzymatic stability.^1–4^ Unlike conventional antisense oligonucleotides (ASOs), PNAs do not recruit RNase H cleavage because their peptide-like backbone differs fundamentally from the charged phosphodiester backbone recognized in RNA/DNA hybrids.^5,6^ This distinction has redirected PNA development toward applications that exploit high-affinity, sequence-selective binding without requiring target degradation. PNAs have been investigated as splice-switching oligonucleotides of pre-mRNA targets, as microRNA binders that modulate regulator networks, and as DNA binders that form sequence-specific duplexes or triplexes for gene regulation.^7–9^ Across these modalities, biological activity requires the formation of a sufficiently stable PNA/nucleic acid complex, determined by the target residence time and binding kinetics, alongside cellular uptake, intracellular localization and target accessibility.^10–13^

**Fig. 1:**
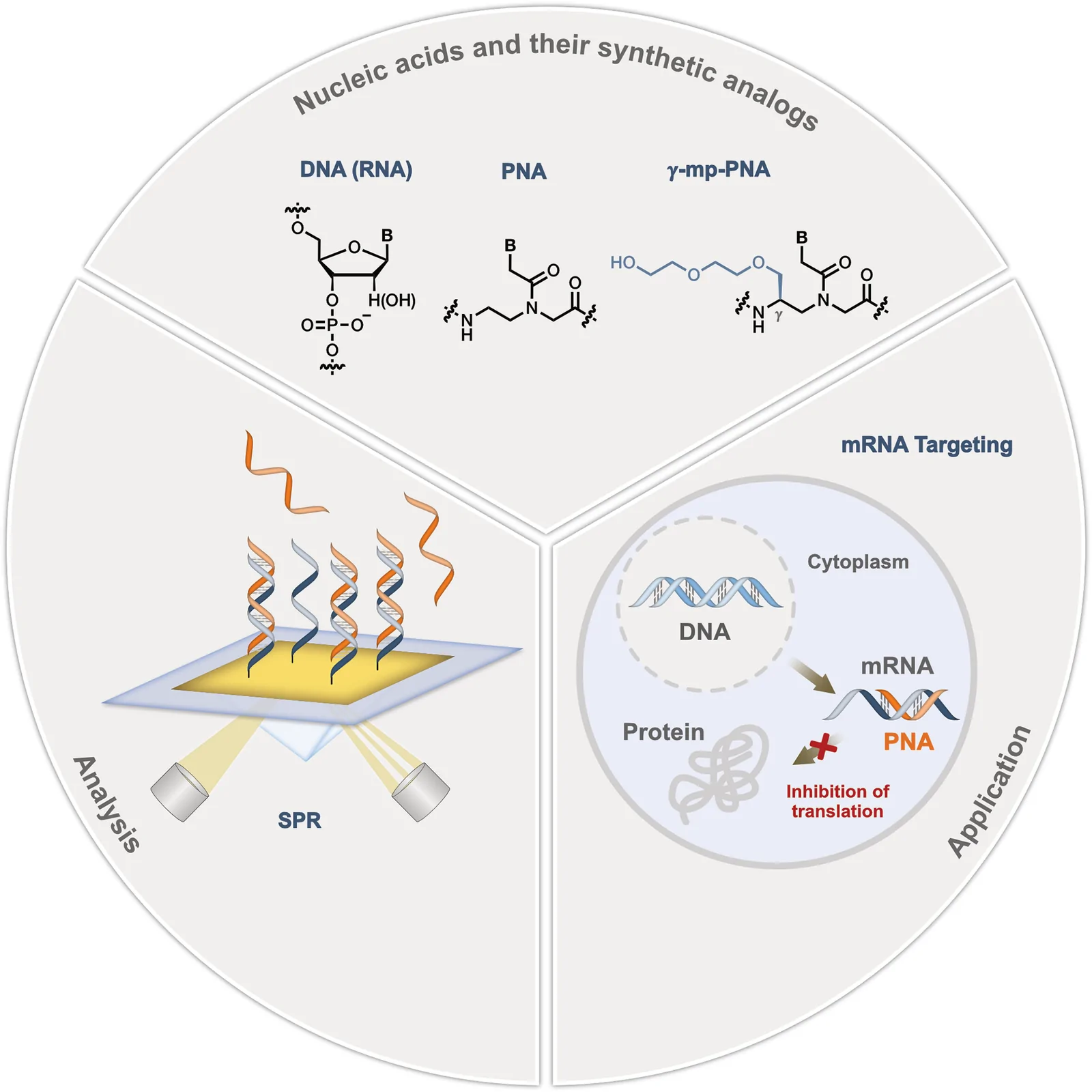
Structures of synthetic PNA and mp-PNA (B = adenine, guanine, cytosine or thymine), analysis of binding kinetics with SPR spectroscopy and application of PNAs.

The neutral PNA backbone provides exceptional high resistance to enzymatic degradation and minimizes electrostatic repulsion with nucleic acid targets. However, conventional PNAs face limitations that restrict broader applicability: poor aqueous solubility, aggregation, non-specific interactions and insufficient pharmacokinetic properties.^14–17^ These challenges have driven the development of chemically modified PNA analogs that improve physicochemical properties while retaining favorable nucleic acid recognition characteristics.

A successful strategy to address these limitations is the incorporation of diethylene glycol in γ-position of the backbone yielding γ-miniPEG-PNAs (mp-PNAs, Fig. 1).^18^ Compared to conventional PNAs, mp-PNAs exhibit enhanced aqueous solubility, reduce aggregation, and induce conformational preorganization of the PNA backbone, thereby influencing the thermodynamic and kinetic properties of PNA-nucleic acid duplex formation. γ-miniPEG modification increases duplex melting temperature (*T*_m_) and improves mismatch discrimination, with the magnitude of these effects depending on the number and position of γ-modifications.^18,19^

Accordingly, γ-miniPEG-modified PNAs have demonstrated considerable promise in nucleic acid recognition and gene-modulation applications, including triplex formation and strand invasion strategies for gene modulation and gene editing.^20–22^ These studies highlight the potential of γ-modified PNAs as versatile nucleic acid recognition agents; rather than as direct replacements for classical RNase H-recruiting ASOs. Understanding how backbone modification affects RNA binding behavior is therefore critical across PNA applications, from splice-switching to microRNA inhibition.

In this context, equilibrium duplex stability, as obtained from *T*_m_ measurements, is an important but incomplete descriptor of target recognition, because the kinetics of target engagement, including the rates of association and dissociation, provide additional information on how rapidly a PNA forms a complex and how long that interaction persists, information that equilibrium measurements alone cannot capture.^11,23^

Several methods can be used to characterize nucleic acid duplex formation, including UV melting analysis, isothermal titration calorimetry, fluorescence-based binding assays, nuclear magnetic resonance spectroscopy and surface plasmon resonance (SPR).^24–26^ UV melting analysis determines thermal duplex stability (*T*_m_) and thermodynamic parameters (Δ*H*, Δ*S*),^27^ but describes only temperature-dependent equilibrium stability of a duplex without resolving individual association and dissociation processes.^11,28^ Moreover, melting experiments probe the interaction across a temperature gradient, whereas biological activity occurs at constant physiological temperature. Such temperature variations can affect nucleic acid conformation and alter association and dissociation.^29^ This consideration is particularly relevant for interactions involving structural rearrangements or slow thermodynamic processes.^30,31^

SPR addresses these limitations by enabling label-free, real-time monitoring of association (*k*_a_) and dissociation (*k*_d_) rates at defined, constant temperatures. By independently determining apparent *k*_a_ and *k*_d_ rate constants, SPR can distinguish duplexes with similar equilibrium affinities but different kinetic pathways^25,32–34^ and provides a direct measure of duplex persistence under the measurement conditions, which is not obtained from *T*_m_ alone.^28^

Although mp-PNA/RNA duplexes have been characterized with respect to thermal stability and structural preorganization, systematic studies of their hybridization kinetics remain limited. Reported SPR studies have commonly used PNA immobilized on the sensor surface to capture complementary RNA targets from solution, including miR-155 and other miRNA targets.^35–37^ In contrast, the inverse configuration, in which RNA is immobilized and PNA (or mp-PNA) is injected as analyte, is less commonly used for kinetic characterization of PNA/RNA hybridization.^11,18,38,39^ A systematic comparison of PNA and γ-modified PNA in this configuration can therefore provide kinetic information that complements existing thermodynamic and structural characterization.

We therefore established a controlled SPR platform with complementary RNA immobilized on the sensor surface and PNA or mp-PNA as a soluble analyte. This configuration keeps the RNA target constant on the sensor surface while comparing PNA architectures under identical conditions. We systematically examined RNA/RNA, PNA/RNA and mp-PNA/RNA duplexes of varying lengths to determine how backbone chemistry and oligomer length influence the association and dissociation phases of hybridization. Terminal lysine was included as a minimal, widely used modification that improves solubility and introduces a single positive charge to partially offset the neutral PNA backbone and enhance electrostatic interactions with the RNA target. We also compared the kinetics of a representative PNA/RNA duplex at 22 and 37 °C. The latter experiment provides an initial assessment of temperature-dependent kinetic behavior at a temperature relevant to biological applications.

By systematically varying nucleotide length, nucleic acid type and γ-modification while keeping the RNA surface and measurement configuration constant, this study delivers a side-by-side kinetic map of how PNA architecture governs PNA/RNA hybridization. These findings provide a quantitative framework for examining how PNA backbone architecture influences the kinetic fingerprints of RNA binding and for identifying PNA designs with association and dissociation characteristics and isothermal conditions that warrant further investigation in solution-phase and functional PNA-targeting studies.

## Results and discussion

### Design and synthesis of nucleic acids

RNAs, PNAs and mp-PNAs were designed to target the nucleotide 705 mutation in the human β-globin gene associated with β-thalassemia,^7^ which was selected as a model target sequence for this study. The thiol-modified 24mer target RNA (Fig. 2A) was used as the target immobilized on gold for all hybridization measurements, while the soluble complementary strands were varied in nucleic acid type and strand. Complementary RNA, PNA and mp-PNA sequences were then designed to bind to the central region of the target RNA. 10mer, 12mer and 14mer constructs were generated by progressively trimming nucleotides from both ends of the longest sequence, thereby maintaining the same central recognition region across all lengths (Fig. 2B). A short 6mer PNA, corresponding to the second half of the 12mer sequence, was also included.

**Fig. 2:**
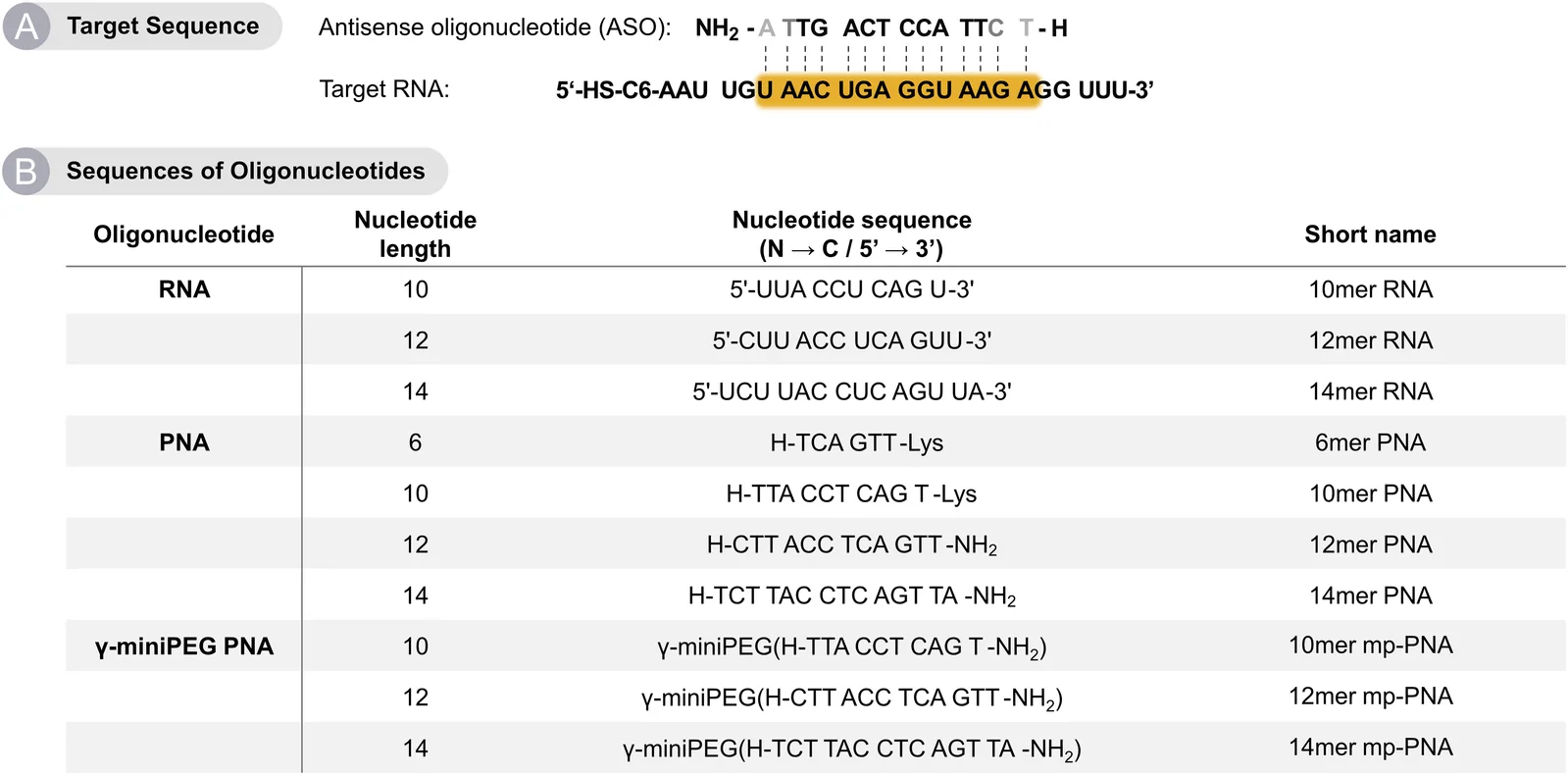
(A) Target sequence with complementary antisense oligonucleotides (for RNA: T = U); (B) sequences of oligonucleotides used for SPR hybridization studies; abbreviations: A = adenine, C = cytosine, G = guanine, T = thymine, U = uracil, H = NH_2_ (N-terminus), NH_2_ = amide (C-terminus), SH = thiol-modification.

Because the stability, aqueous solubility, and resistance to non-specific binding and aggregation of mp-PNAs can be improved by increasing the number of miniPEG groups in the backbone,^18^ mp-PNAs were prepared with miniPEG groups incorporated throughout the PNA backbone. Lysine was introduced into the 6mer and 10mer PNAs to investigate how a single positive charge influences the binding behavior of short PNA sequences. This modification was included to assess whether introducing a positive charge can measurably alter the interaction of short PNA oligomers with the immobilized RNA target under the applied SPR conditions. As a negative control, a scrambled 12mer PNA (sc-PNA) was designed to assess non-specific surface binding. The sequence contains the same nucleobase composition as the complementary PNA but in a randomized order (see SI section 5.12).

PNAs and mp-PNAs were synthesized *via* solid-phase peptide synthesis using the Boc-protected monomers and HBTU-mediated coupling according to known procedures (see SI section 2).^40^ After cleavage, precipitation and purification by reversed-phase column chromatography, the purified PNAs were desalted under neutral conditions prior to SPR hybridization experiments.

### Surface preparation

For this systematic kinetics study, thiolated RNA was directly immobilized on gold-coated sensor chips because this approach offers a straightforward and reproducible surface preparation while enabling label-free detection of hybridization events.^41–43^ To optimize RNA surface density while minimizing non-specific interaction, immobilization was first tested using thiol-RNA concentrations of 3 and 1 µM, either alone or in combination with 6-mercapto-1-hexanol (MCH) at RNA : MCH ratios of 1 : 1, 1 : 9 and 1 : 15 in buffer. Among the conditions tested, the 1 : 15 RNA : MCH ratio provided the most favorable hybridization response (see SI section 1.3) and was therefore selected for subsequent experiments.

For kinetic analysis, RNAs, PNAs and mp-PNAs were prepared at concentrations of 6.25–1600 nM in running buffer. Unless otherwise stated, hybridization measurements were performed at 22 °C to maintain consistent experimental conditions across the different nucleic acid constructs.^44^ Measurements at 37 °C were additionally performed for the representative 12mer PNA/RNA system to assess the effect of temperature on the observed kinetic behavior at a temperature relevant to biological applications. Samples were injected over the immobilization surface in a four-channel flow cell for 4 min at 22 °C (1 min for the 6mer PNA) or 2 min at 37 °C, followed by a dissociation for 40 min at 22 °C (5 min for the 6mer PNA) or 10 min at 37 °C. The shorter association and dissociation periods used for the 6mer PNA were selected based on the observed rapid equilibrium and the resulting low change in SPR response during extended measurements; extending these periods did not measurably affect the fitted kinetic parameters. Between measurements, the surface was regenerated with a high-salt/alkaline buffer (NaOH/NaCl), as commonly used for regeneration in nucleic acid SPR assays.^45–47^ Complete removal of the hybridized strand was confirmed by return of the SPR response to the pre-injection baseline, while recovery of the binding response in subsequent injections indicated preservation of the functionalized surface (see SI section 5.15). Association (*k*_a_) and dissociation (*k*_d_) rate constants were obtained by fitting the SPR sensorgrams to a 1 : 1 Langmuir binding model, and the corresponding equilibrium dissociation constants (*K*_D_) were calculated from *k*_d_/*k*_a_.

### Hybridization kinetics

Using this kinetic framework, first we assessed the influence of oligomer length (10 to 14mers) on hybridization of RNA, PNA and mp-PNA to surface-immobilized RNA. Across the 10–14mer series, the association was largely independent of oligomer length, consistent with previous reports.^39^ In contrast, dissociation was strongly length dependent (Fig. 3B). Increasing oligomer length systematically decreased dissociation, indicating increased kinetic stability of the resulting duplexes. This length dependence was observed for all three nucleic acid classes, although the magnitude of the effect differed substantially, with mp-PNA showing the strongest dependence on oligomer length. For RNA and PNA, *k*_d_ was 2.9- to 3.8-fold higher for the 10mer than for the 14mer, whereas mp-PNA showed a more pronounced 8.8-fold difference. For mp-PNA, extending the sequence by two nucleotides reduced *k*_d_ by 2.4- to 3.6-across the 10–14mer series. Association and dissociation rate constants are summarized in Fig. 3A. The pronounced length dependence of dissociation is consistent with the expected contribution of additional base pairs to duplex stability. For PNA/RNA duplexes, the neutral PNA backbone additionally eliminates the electrostatic repulsion associated with two negatively charged nucleic acid strands.^15^ However, the neutral PNA backbone also lacks favorable electrostatic attraction to the negatively charged RNA target. We therefore asked whether introducing a terminal positively charge could improve the binding behavior of short PNA sequences. Because short PNA/RNA duplexes are intrinsically less stable than longer duplexes, we hypothesized that an additional positive charge might have a proportionally larger effect on the binding of short PNA sequences. To test this hypothesis, a lysine residue was introduced at the C-terminus of 6mer and 10mer PNA sequences.

**Fig. 3:**
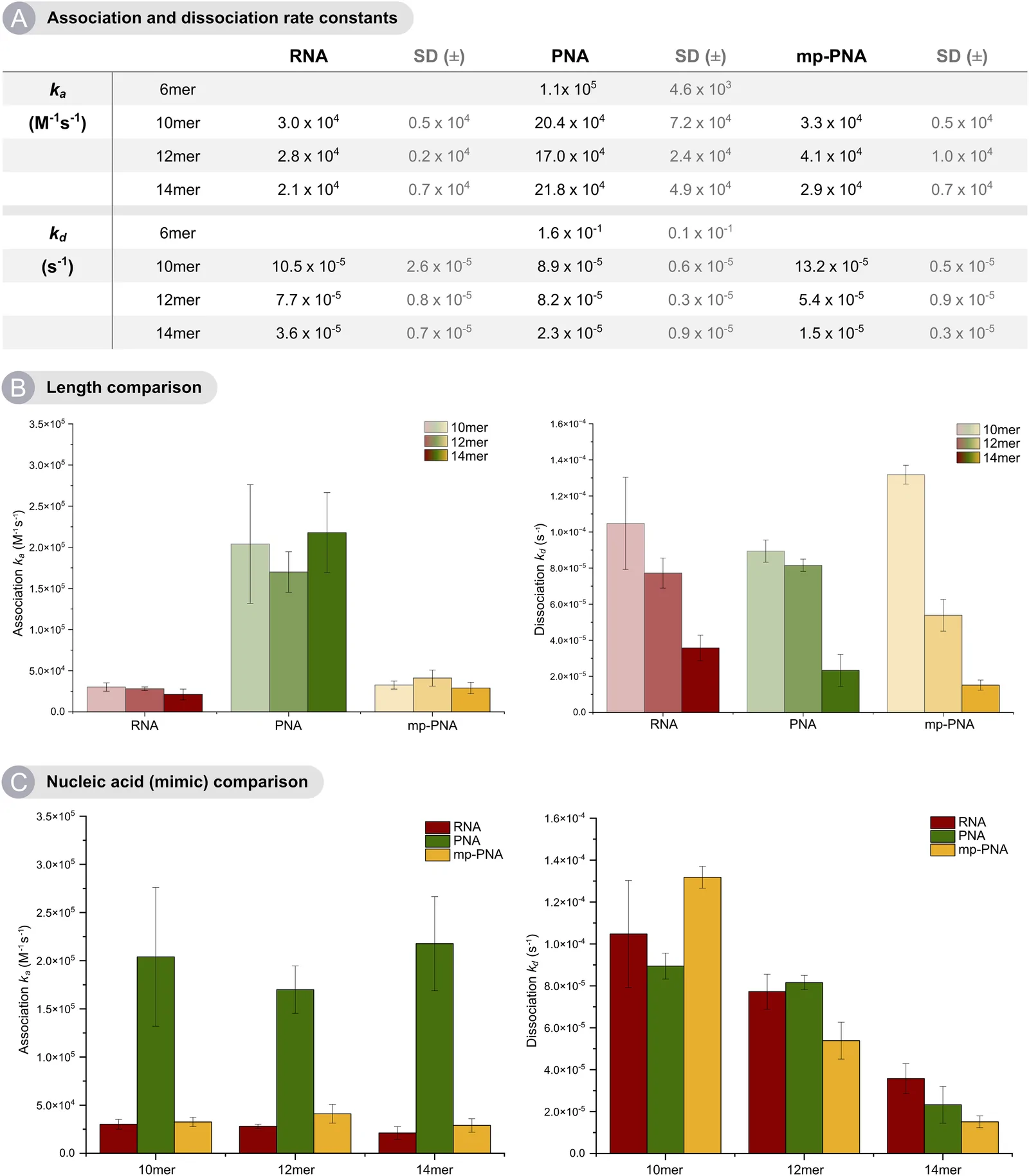
(A) Association and dissociation rate constants: *k*_a_ and *k*_d_ of 6, 10, 12 and 14mer RNA, PNA and mp-PNA measured *via* SPR (*n* = 6–8); SD = standard deviation; (B) length comparison of association and dissociation of 10 (light color), 12 (middle color) and 14mer (dark color) RNA, PNA and mp-PNA measured *via* SPR (*n* = 6–8); (C) nucleic acid (mimic) comparison of association and dissociation: comparison of RNA (red), PNA (green) and mp-PNA (yellow).

Under identical conditions, the unmodified 6mer PNA exhibited negligible hybridization, consistent with the low stability expected for a short PNA/RNA duplex (see SI section 5.13).

In contrast, the lysine-modified 6mer produced a measurable SPR response but also displayed the fastest dissociation of all sequences investigated, with a *k*_d_ approximately three orders of magnitude higher than that of the lysine-modified 10mer (Fig. 4). Thus, the additional positive charge enabled measurable binding of the 6mer but did not compensate for the intrinsically low kinetic stability of the short duplex. A similar behavior was observed for the unmodified 10mer PNA (see SI section 5.14).

**Fig. 4:**
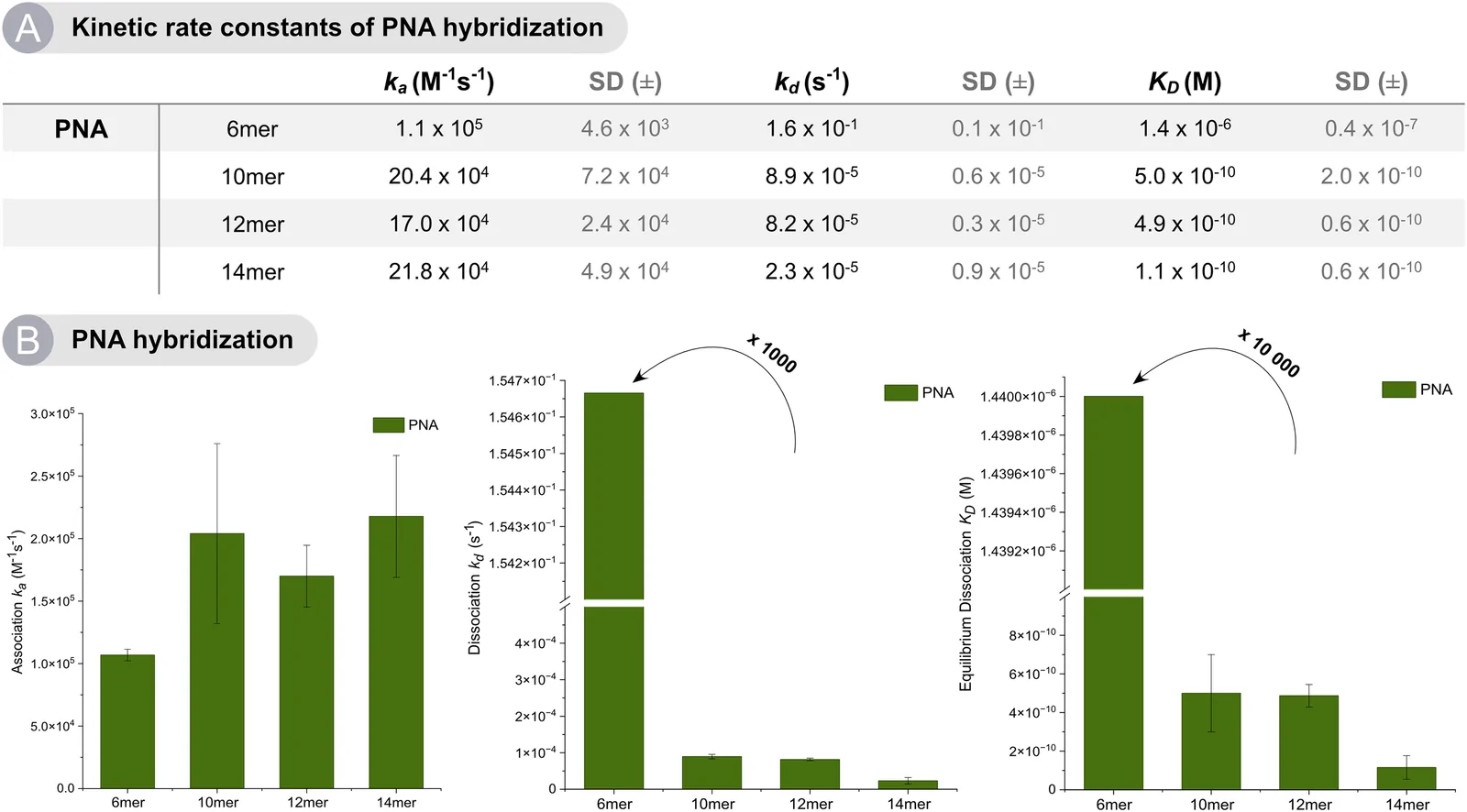
(A) Kinetic rate constants (*k*_a_, *k*_d_ and *K*_D_) of 6, 10, 12 and 14mer PNA measured *via* SPR (*n* = 6–8), (B) PNA length comparison.

Introducing lysine at the C-terminus of the 10mer PNA resulted in a more reproducible concentration-dependent SPR response; however, the irregular concentration dependence observed for the unmodified 10mer prevented a quantitative kinetic comparison. This behavior is consistent with the contribution from the additional positive charge, although the present SPR measurements do not allow its electrostatic contribution to be separated from other surface-dependent effects. In contrast, lysine modification was not required to obtain measurable hybridization of the 10mer RNA and mp-PNA under the same conditions. For mp-PNA, this behavior is consistent with the improved physicochemical properties associated with miniPEG-modification, including increased aqueous solubility and reduced aggregation.^18^ However, the present experiments do not allow the relative contributions of these properties to the observed surface binding behavior to be separated.

Taken together, the length series reveals a clear distinction between association and dissociation kinetics. The association rate was largely independent of oligomer length across 10–14mer series and decreased only modestly for the 6mer PNA (approximately threefold). In contract, dissociation was strongly length dependent, with longer oligomers displaying substantially lower *k*_d_ values (Fig. 3). Backbone chemistry had a comparatively modest effect on dissociation, with *k*_d_ values differing by only 0.8- to 2.4-fold relative to RNA. In contrast, substantially larger differences were observed in the association kinetics (Fig. 3C). Association rate constants for RNA and mp-PNA were similar, with mp-PNA associating 1.1- to 1.5-fold faster than RNA, whereas unmodified PNA showed substantially faster association, with *k*_a_ values 5- to 8-fold higher than those of RNA. The faster association observed for unmodified PNA is consistent with a reduced electrostatic penalty relative to the association of two negatively charged nucleic acid strands.^9,13^ Although γ-modification is expected to preorganize the PNA backbone and thereby reduce the entropic cost of hybridization, the mp-PNA in this study associated more slowly than unmodified PNA.

Fully modified mp-PNA showed approximately sixfold slower association, whereas Sahu *et al.* reported the opposite trend, with mp-PNA associating nearly twice as fast.^18^ One possible explanation for this trend is that miniPEG side chains impose steric constraints: while preorganization should accelerate binding, the bulky miniPEG substituents can hinder approach and optimal alignment to the target RNA. Molecular simulations support this view, indicating that miniPEG side chains can invade the grooves and disrupt Watson–Crick base pairing and stacking.^48^ Importantly, prior reports of accelerated association for γ-modified PNAs have focused on partially substituted (mixed PNA/mp-PNA) strands; in contrast, our fully γ-substituted mp-PNAs may experience more pronounced steric hindrance, tipping the balance away from a net kinetic benefit. Furthermore, prior studies differ in target identity (DNA *vs.* RNA), the immobilized strand, and key assay conditions such as buffer composition (salt concentration, chelators, surfactants), temperature, and pH, as well as in immobilization strategies (non-covalent biotin/streptavidin *vs.* covalent thiol chemistry). These variables may alter target orientation, accessibility, local surface environment and steric constraints, and may therefore affect the apparent association kinetics of modified PNA differently from those of unmodified PNA.^18,49^ Furthermore, ionic strength has been shown to influence PNA hybridization kinetics, particularly the association rate may decrease with increasing ionic strength,^50,51^ and therefore differences in buffer composition may contribute to differences in the apparent *k*_a_ values between studies. Accordingly, the *k*_a_ values obtained in this study should be interpreted primarily as comparative kinetic parameters within the present surface and experimental configuration (using a physiological, Ca^2+^- and Mg^2+^-free buffer eliminating the need of a chelator), rather than as universal intrinsic association rate constants for RNA/PNA hybridization in solution.

Because *K*_D_ was calculation from *k*_d_/*k*_a_, the differences in association and dissociation kinetics were reflected directly in the resulting equilibrium dissociation constants. PNA exhibited approximately 6- to 16-fold lower *K*_D_ values than RNAs, and approximately 3- to 8-fold lower values than mp-PNA. Across the 10–14mer series, *K*_D_ generally decreased with increasing oligomer length, consistent with the stronger kinetic stability observed for longer duplexes. The effect was most pronounced for mp-PNA, for which *K*_D_ decreased by approximately 8-fold from 10 to 14mer. PNA showed a 4.5-fold decreases, whereas RNA showed a less than 2-fold change (Fig. 5). As expected from its rapid dissociation, the 6mer PNA exhibited markedly different *K*_D_ behavior from the longer PNA sequences. These results further support the conclusion that very short PNA/RNA interactions are kinetically unstable under the present surface conditions. Summarized kinetic data and a graphical depiction are provided in Fig. 5.

**Fig. 5:**
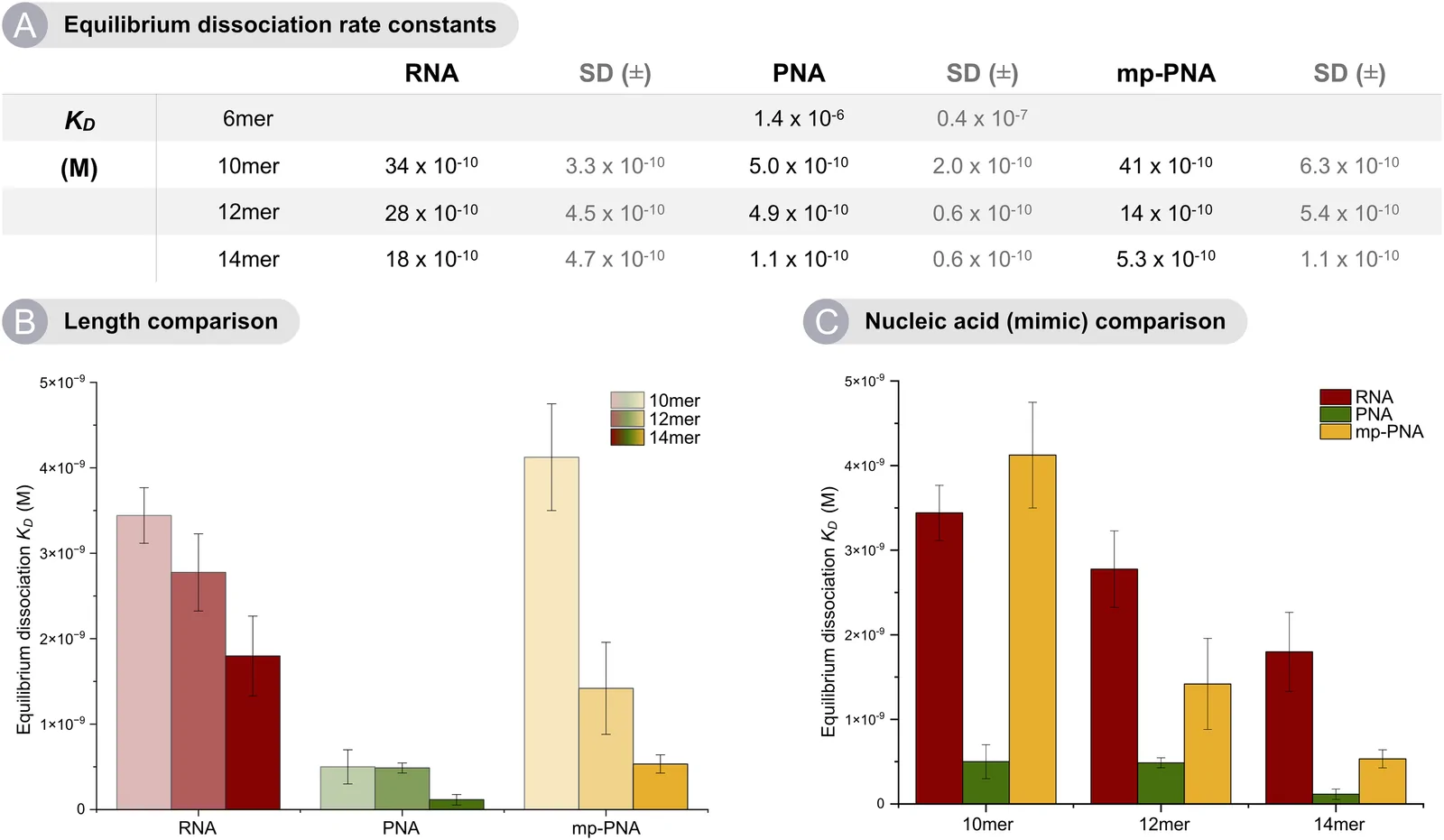
(A) Equilibrium dissociation rate constants (*K*_D_) of 10, 12 and 14mer RNA, PNA and mp-PNA measured *via* SPR (*n* = 6–8), (B) length comparison, (C) nucleic acid (mimic) comparison.

Comparison of 12mer PNA/RNA hybridization at 22 and 37 °C revealed substantially faster association and dissociation at the higher temperature. The association rate constant increased by a factor of 3.7, while the dissociation rate constant increased 4.5-fold at 37 °C (Fig. 6). Despite these pronounced changes in both kinetic parameters, the resulting equilibrium dissociation constant remained within a similar range. Thus, similar apparent affinities can arise from substantially different combinations of association and dissociation rates. This comparison illustrates that *K*_D_ alone does not capture the kinetic behavior of PNA/RNA hybridization and that consideration of both *k*_a_ and *k*_d_ provides additional information on the dynamics of duplex formation and dissociation.

**Fig. 6:**
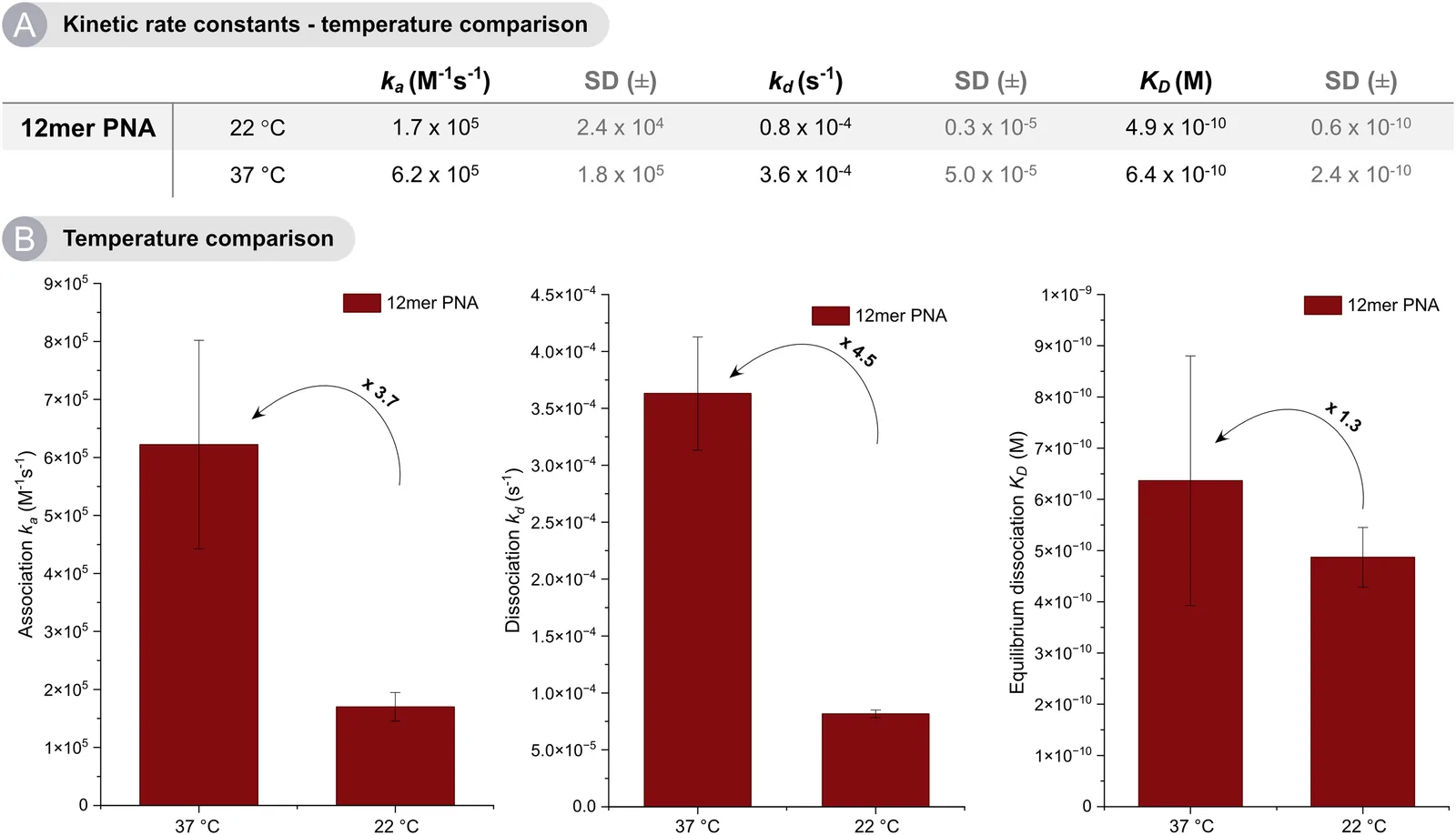
(A) Kinetic rate constants (*k*_a_, *k*_d_ and *K*_D_) of 12mer PNA at 37 and 22 °C measured *via* SPR (*n* = 6–8), (B) Temperature comparison of 12mer PNA at 37 and 22 °C.

## Conclusions

This study provides a systematic comparison of the hybridization kinetics of RNA, PNA and γ-miniPEG-modified PNA (mp-PNA) using complementary sequences targeting nucleotide 705 of the human β-globin gene as a model system. By varying oligomer length and backbone chemistry while maintaining a common immobilized RNA target, we distinguish the effects of sequence length and backbone modification on association and dissociation kinetics. Across the 10–14mer series, dissociation kinetics were strongly dependent on oligomer length, with longer sequences exhibiting substantially lower *k*_d_ values, whereas association rates were comparatively insensitive to length. In contrast, backbone chemistry had a stronger effect on association than on dissociation: unmodified PNA associated 5–8-fold faster than RNA, while mp-PNA showed association rates similar to those of RNA. These results demonstrate that association and dissociation kinetics can respond differently to changes in oligomer architecture and that sequence length and backbone chemistry contribute differently to the observed hybridization behavior.

The introduction of a terminal lysine residue was explored as a means of facilitating hybridization of short PNA sequences. Under the present experimental conditions, lysine modification enabled a detectable SPR response of the 6mer PNA, whereas the corresponding unmodified 6mer showed no clearly detectable concentrations-dependent response. For the 10mer PNA, lysine modification was associated with a more reproducible concentration-dependent response, although the available measurements do not permit a quantitative determination of the effect of lysine on *k*_a_ and *k*_d_. However, the very rapid dissociation of the lysine-modified 6mer demonstrates that the additional positive charge did not compensate for the intrinsically low kinetic stability of very short PNA/RNA duplexes. The effect of lysine was therefore most apparent for short PNA sequences and was not required for efficient hybridization of RNA or mp-PNA under the conditions investigated.

Comparison of the 12mer PNA/RNA system at 22 and 37 °C showed that increasing temperature accelerated both association and dissociation, while the resulting *K*_D_ values remained within similar range. This illustrates that similar apparent equilibrium affinities can arise from substantially different combinations of association and dissociation rates and highlights the additional information provided by SPR kinetic analysis.

Overall, this work supports the potential of mp-PNA as a promising backbone modification for antisense applications, combining favorable hybridization kinetics with the established advantages of improved aqueous solubility and reduced aggregation. The systematic kinetic analysis presented here provides additional parameters for evaluating PNA candidates beyond equilibrium affinity alone and may help streamline the selection of promising sequences for subsequent cellular studies. Ultimately, integrating kinetic properties with factors such as target accessibility, stability, administration and intracellular delivery will be important for translating these molecular features into effective antisense applications.

## Author contributions

B. Meindl: data curation, formal analysis, investigation, validation, visualization, writing – original draft, review & editing. A. Lenz: data curation, formal analysis, investigation, validation, visualization, writing – original draft. M. Schittmayer: data curation, formal analysis, investigation, writing – original draft. H. Mikula: conceptualization, funding acquisition, supervision, resources and writing – original draft. B. Holzer: conceptualization, funding acquisition, resources, supervision, validation and writing – original draft, review & editing. All authors have given approval to the final version of the manuscript.

## Conflicts of interest

There are no conflicts to declare.

## Supplementary Material

OB-024-D6OB01345G-s001

## Data Availability

The data supporting this article have been included as part of the supplementary information (SI), including detailed experimental procedures, synthesis and characterization of PNAs, SPR kinetic data and sensorgrams, and results of control experiments.18,40 Supplementary information is available. See DOI: https://doi.org/10.1039/d6ob01345g. Raw data for SPR sensorgrams and corresponding fitted curves underlying the graphs presented in the supplementary information are available as txt files in https://researchdata.tuwien.ac.at/records/0kg53-vn725.
