# Supplementary material for: Comparative kinetic analysis of PNA and γ-miniPEG-PNA hybridization with RNA by surface plasmon resonance
Source: Org Biomol Chem. 2026 Oct 16. Online ahead of print. doi: 10.1039/d6ob01345g (PMC13688168; doi:10.1039/d6ob01345g)
Supplement: Supplementary file 1 [file OB-OLF-D6OB01345G-s001.pdf]

Supplementary Information (SI) for Organic & Biomolecular Chemistry.
This journal is © The Royal Society of Chemistry

Supporting Information

Comparative Kinetic Analysis of PNA and γ-MiniPEG-PNA Hybridization with RNA by Surface Plasmon Resonance

*Birgit Meindl,[1‡] Agnes Lenz,[1‡] Matthias Schittmayer,[2] Hannes Mikula[1] and Brigitte Holzer[1]**

[1]TU Wien, Institute of Applied Synthetic Chemistry, Getreidemarkt 9, 1060 Vienna, Austria

[2]TU Wien, Institute of Chemical Technology and Analytics, Getreidemarkt 9, 1060 Vienna, Austria

Table of contents

1. Materials and Methods 2
1.1. Materials 2
1.2. Analytical and semi-preparative chromatographic methods 2
1.3. Surface plasmon resonance (SPR) spectroscopy 3
2. Synthesis of PNAs and mp-PNAs 4
3. Structures and Analytics of Synthesized PNAs and mp-PNAs 6
3.1. 6mer PNA 6
3.2. 10mer PNA 8
3.3. 12mer PNA 10
3.4. 14mer PNA 12
3.5. 10mer mp-PNA 13
3.6. 12mer mp-PNA 14
3.7. 14mer mp-PNA 15
4. SPR Kinetic Data of RNAs, PNAs and mp-PNAs 16
5. SPR Sensorgrams of RNAs, PNAs and mp-PNAs 20
5.1. 10mer RNA 20
5.2. 12mer RNA 21
5.3. 14mer RNA 22
5.4. 6mer PNA 23
5.5. 10mer PNA 24
5.6. 12mer PNA 25
5.7. 14mer PNA 26
5.8. 10mer mp-PNA 27
5.9. 12mer mp-PNA 29
5.10. 14mer mp-PNA 30
5.11. 12mer PNA 37 °C 32
5.12. Control experiment using scrambled PNA 33

5.13. Sensorgram of 6mer PNA without lysine modification 33
5.14. Sensorgram of 10mer PNA without lysin modification..34
5.15. Control of surface regeneration 34
6. References 36

1. Materials and Methods

1.1. Materials

Dulbecco's phosphate-buffered saline (dPBS containing: 0.2 g/L KCl, 0.2 g/L KH_2PO_4, 8.0 g/L NaCl, 1.15 g/L Na_2HPO_4 (anhydrous)) was purchased from Merck. Thiolated target RNA (5'-thiol C6-AAU UGA AAC UGA GGU AAG AGG UUU-3') and antisense strand RNAs (5'-CCU CUU ACC UCA GUU ACA-3', 5'-UCU UAC CUC AGU UA-3', 5'-CUU ACC UCA GUU-3', 5'-UUA CCU CAG U-3') were purchased from biomers.net. All PNAs and mp-PNA were synthesized according to the solid-phase peptide synthesis (SPPS) protocol described below. MBHA resin LL (100-200 mesh).HCl was purchased from Merck and Boc-Lys(2-Cl-Z)-MBHA resin (100-200 mesh, 0.7 mmol/g) from Advanced Chemtech. PNA monomers (adenine, cytosine, guanine and thymine) were obtained from PolyOrc.Inc. mp-PNA monomers were synthesized following the procedure of Sahu et al.[1]

1.2. Analytical and semi-preparative chromatographic methods

Semi-preparative column chromatography and desalting was performed on a Nexera Prep® system (Shimadzu®) comprised of LC-20AR pumps, manual injector, CTO-40C column oven, DGU-405 degassing unit and FRC-10A fraction collector. Detection was done using an SPD-M40 photo diode array. Separations were performed using a Phenomenex® Luna® C18(2) 5 μm (100 Å, 250 x 4.6 mm), Phenomenex® Luna® C8(2) 5 μm (100 Å, 250 x 4.6 mm) or Waters XBridge® BEH Amide (HILIC) 5 μm (250 x 4.6 mm) column at 40 °C and a flowrate of 1.2 mL/min with 0.1% aqueous TFA, ammonium formate buffer (2.5 mM, pH 8.5) or neutral H_2O and ACN (5-95% gradient elution).

HPLC analysis (LC-MS) was performed on a Nexera HPLC system (Shimadzu®) system (Shimadzu®) comprised of LC-40D XR pumps, SIL-40C XR autosampler, CTO-40AC column oven and DGU-405 degasser module. Detection was done using an SPD-M40 photo diode array, an ELSD-LTIII evaporative light scattering detector (Shimadzu®) and an LCMS-2050 mass spectrometer (DUIS). If not stated otherwise, all separations were performed using a Waters™ XSelect® CSH™ C18 XP Column, 130 Å, 3.5 μm (3.0 x 50 mm) column at 40 °C, and a flowrate of 1.7 mL/min with 0.1% aqueous formic acid ("acidic") or ammonium

formate buffer ("basic", 2.5 mM, pH 8.5) and ACN (gradient elution): 0 min: 5%, 0.15 min: 5%, 2.20 min: 98%, 2.50 min: 98%.

High resolution mass spectra (TOF-MS) were acquired employing a Bruker Daltonics timsTOF Pro mass spectrometer coupled to a Thermo Fisher Scientific Vanquish H UHPLC system. Chromatography was carried out on a Waters Acquity UPLC BEH C18 column, Dimensions 1 mm x 150 mm x 1.7 µm. The following gradient was applied at a constant flow of 100 µL/min, with A being H_2O/0.1% formic acid and B being acetonitrile/0.1% formic acid: 0 min: 0-2 min, 2% B; linear increase to 98% B until 7 min. Hold 98% for 2 min, followed by a re-equilibration step to 2% B for 1 min. The LC was coupled to the mass spectrometer employing a VIP-HESI source operated in positive polarity. The mass spectrometer was operated in DDA PASEF mode at the standard 1.1 sec cycle time with a mass range of 100–1700 m/z. Data Analysis was conducted on Bruker Daltonics Data Analysis 6.0.

1.3. Surface plasmon resonance (SPR) spectroscopy

For optimization of the target RNA immobilization solution, experiments with different RNA concentrations and dilutions with MCH were conducted on a BioNavis MP-SPR NaviTM 210A VASA instrument equipped with two laser sources (670 nm and 785 nm wavelengths). Au-coated (~50 nm) SPR slides comprising a chromium adhesion layer (~2 nm) were also provided by BioNavis. For flushing the system and washing steps, a dPBS buffer from Sigma Aldrich (Dulbecco´s Phosphate Buffered Saline) was used. Data analysis was carried out with Tracedrawer™ and Layersolver (from BioNavis). A gold slide was cleaned with ozone, rinsed with Milli-Q water, dried under a stream of argon and immersed in the target RNA solution in PBS for 16-24 h. The gold slide was rinsed with Milli-Q water, dried under a stream of argon and put into the SPR device. dPBS was pumped through the flow cell for about 5 min with a flow of 10 µL/min, afterwards a 1 mM MCH solution in dPBS was injected for blocking free gold sites. After washing with a 10 mM NaOH solution and dPBS twice and obtaining a stable baseline, hybridization with complementary 18mer RNA was tested. Different dilutions of RNA:MCH (pure PNA, 1:9 and 1:15) were tested at various incubation times to optimize the efficient hybridization and surface densities were calculated. The optimized conditions of a 1 µM 1:15 RNA:MCH solution in buffer resulted in ideal hybridization efficiency, also on the Biacore SPR instrument, and were implemented for the kinetic study. The data is summarized in Table 1.

*Table 1: Optimization of immobilization solution for SPR experiments; measured on a BioNavis MP-SPR NaviTM 210A VASA instrument; *no equilibrium was reached in this timeframe.*

Immobilization solution	Surface density (molecules*cm^{-2})	SD (±)	Injection time (min)
3 µM	1.92 x 10^{13}		69
1 µM	1.95 x 10^{13}	1.02 x 10^{13}	69
1:1	1.38 x 10^{13}	3.65 x 10^{12}	19*
1:9	3.47 x 10^{13}	3.17 x 10^{13}	24
1:15	2.52 x 10^{13}	2.25 x 10^{13}	19

SPR kinetic measurements were performed on a Biacore T200 system from Cytiva. A gold coated glass chip (purchased from Cytiva) was purified in an ozone cleaner (Ossila) for 15 min and washed with boiling water. After drying under an argon stream, the chip was immersed in a 1 µM thiol-RNA solution (1:15 diluted with MCH) in dPBS overnight. After washing with water and drying under an argon stream, the immobilized chip was docked into the SPR cell and dPBS running buffer was pumped over the surface with a flow rate of 30 µL/min. After blocking free gold sites with a 1 mM MCH solution in dPBS and washing with a 1 M NaCl in 50 mM NaOH solution in water, the surface was checked with 3 consecutive hybridizations, with a regeneration step (1 M NaCl in 50 mM NaOH) in between. Hybridization kinetics were measured in a multi-cycle kinetics method with a series of different concentrations in the range of 6.25 - 1600 nM of anti-strand RNA, PNA or mp-PNA. Nucleic acids were injected for 4 min (1 min for 6mer PNA) at 22 °C and 2 min at 37 °C and after a dissociation time of 40 min (5 min for 6mer PNA) at 22 °C and 10 min at 37 °C, the surface was regenerated with NaOH/NaCl between injections of different concentrations. To ensure a stable RNA surface, one concentration was measured at the beginning and end of the measurement (Section 5.15). The SPR curves were fitted and kinetic constants calculated using Biacore Evaluation Software 3.2.1.

2. Synthesis of PNAs and mp-PNAs

A standard solid-phase peptide synthesis (SPPS) protocol applying the Boc-strategy following an adapted protocol of Christensen et al.[2] was carried out in a glass vessel equipped with a membrane, a nitrogen inlet and a vacuum outlet. Nitrogen bubbling was used for stirring. The resin was swelled in DMF for 24 h, washed with DMF:DCM (1:1) three times and dried with DCM. This washing procedure was applied after every step. In case of Lys-modified resin, first deprotection of the Boc-protected amine was done with TFA/m-cresol (95:5) for 10 min before the first coupling step, followed by a washing step. For each coupling, 5 eq amino acid monomer was activated with 4.5 eq HBTU and 10 eq *N,N*-diethylcyclohexylamine (DECA) in pyridine (0.1 M) for 2 min before applying the solution to the resin. For mp-PNAs, this solution was prepared in halve and

added in two portions about 30-60 min apart with a washing step in between. The progress of coupling was monitored using the Kaiser test. If the Kaiser test was positive, either the coupling time was extended, or a new coupling solution was reapplied. After each complete coupling, possible unreacted amine groups were capped with acetic acid anhydride/DCM (1:1) for 15 min. Before the next coupling step, the Boc-protected amine groups were deprotected using TFA/m-cresol (95:5) for 10 min. For cleaving and global deprotection, a solution containing m-cresol, thioanisol, TFA and TFMSA (1:1:5:2) was applied for 2 h. After collecting the cleaving solution and removing volatile compounds, PNAs were precipitated and washed several times in ice-cold anhydrous ether. After drying under reduced pressure, PNAs were purified via reversed phase chromatography (conditions vary depending on PNA class and sequence length, see supporting information). Desalting was performed under neutral conditions. PNA yields were calculated from OD (optical density) values determined at 260 nm using the Lambert Beer equation, with extinction coefficients for cytosine = 6.6, adenine = 13.7, guanine = 11.7 and thymine = 8.8 mL / (μmol x cm). Absorption measurements were conducted on a NanoDrop One© UV/Vis Spectrophotometer from ThermoFischer Scientific. The samples were dissolved in water and stored at -20 °C until use.

3. Structures and Analytics of Synthesized PNAs and mp-PNAs

3.1. 6mer PNA

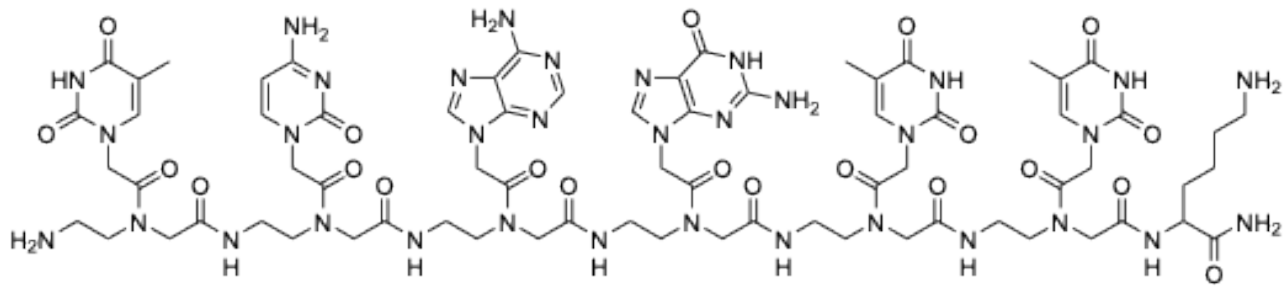

TCA GTT-Lys
Exact Mass: 1760,75

Sequence: H-TCA GTT-Lys

Calculated mass: 1760.75 g/mol

Masses found: 881.3797 $[M+2H]^{2+}$, 587.9229 $[M+3H]^{3+}$, 441.1943 $[M+4H]^{4+}$

□ 1760.7481 g/mol

Chromatographic conditions: C18, 0.1% TFA in H_2O/ACN

TOF-MS: C18, acidic

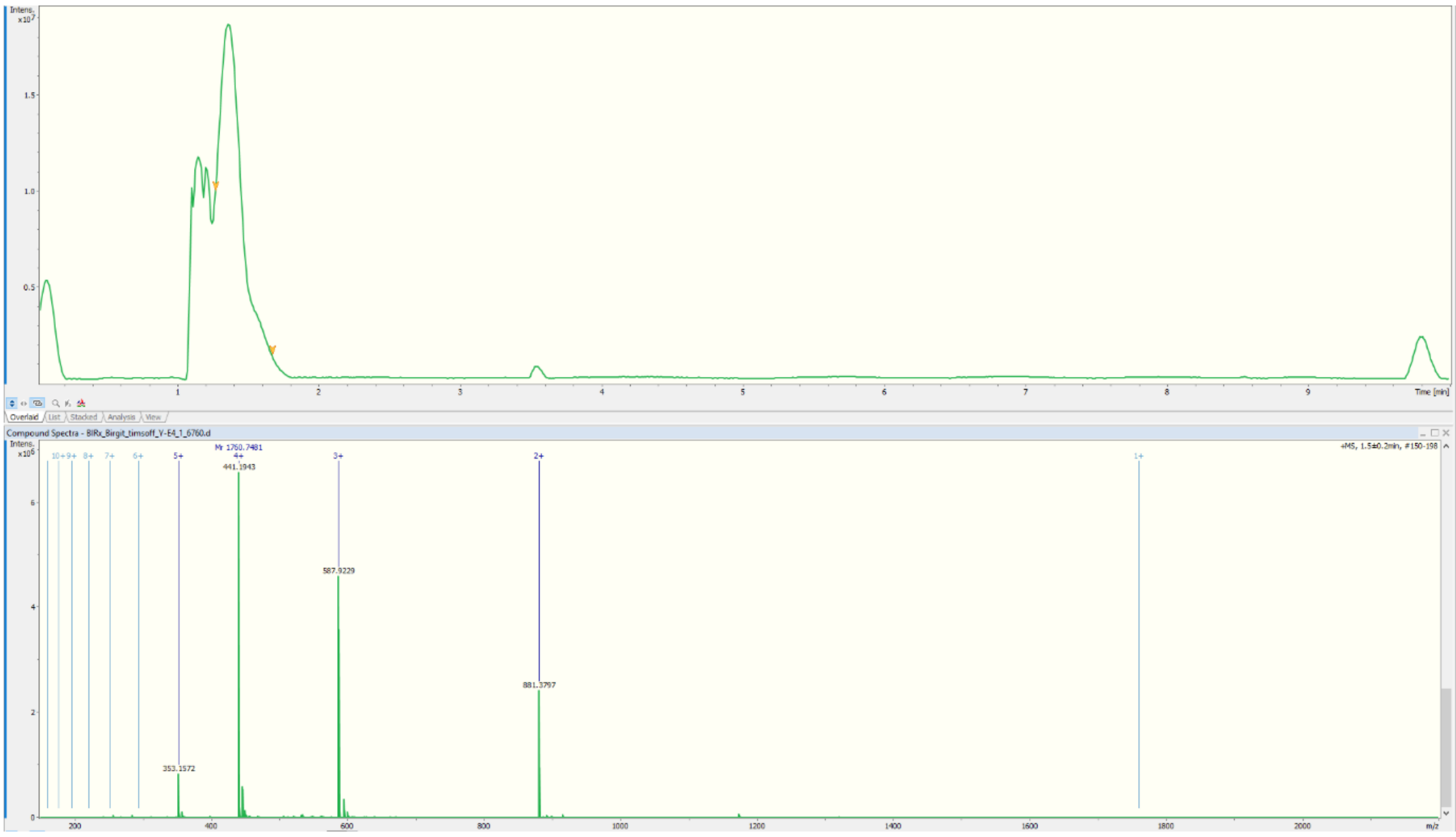

LC-MS: C18, basic

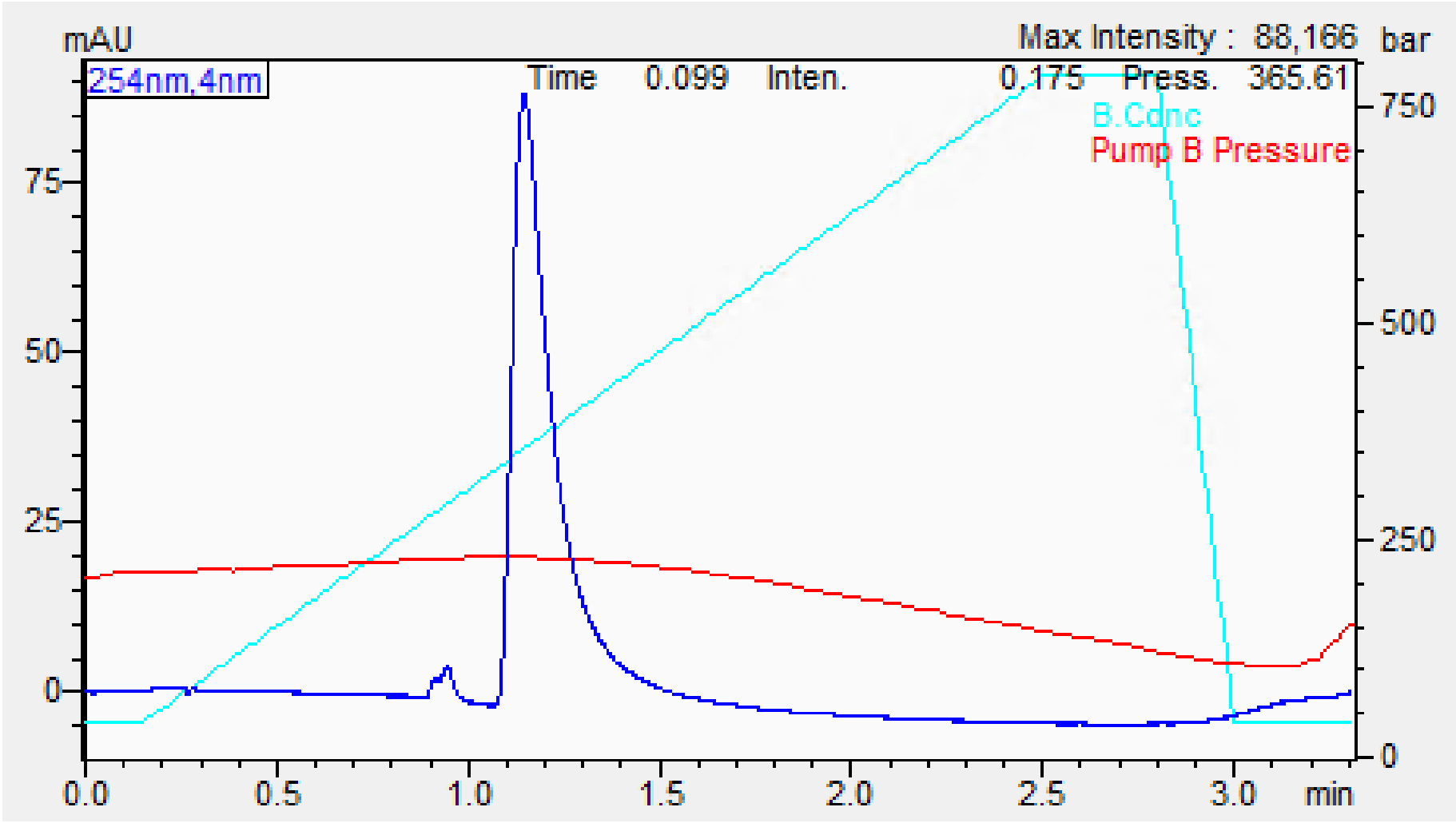

mAU
Max Intensity : 88,166
bar
254nm,4nm
Time 0.099 Inten. 0.175 Press. 365.61
B.Conc
Pump B Pressure
75
50
25
0
750
500
250
0
0.0
0.5
1.0
1.5
2.0
2.5
3.0
min

3.2. 10mer PNA

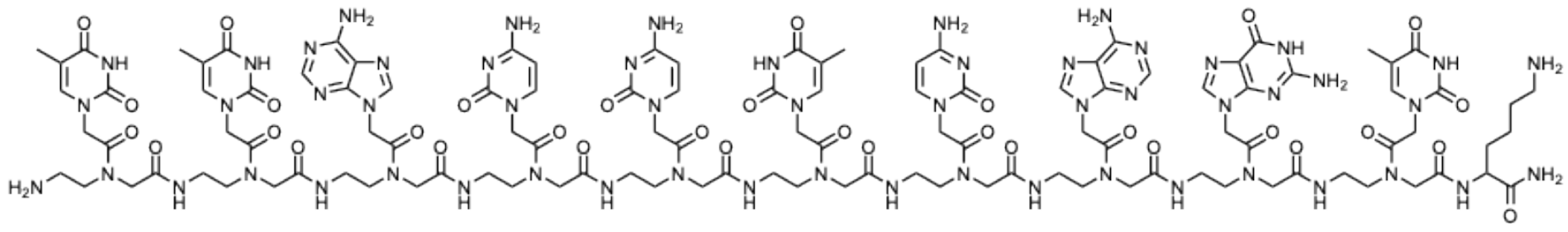

H-TTA CCT CAG T-Lys

Exact Mass: 2804,17

Sequence: TTA CCT CAG T-Lys

Calculated mass: 2804.17 g/mol

Masses found: 1403.5867 $[M+2H]^{2+}$, 936.0610 $[M+3H]^{3+}$, 702.2980 $[M+4H]^{4+}$

□ 2804.1585 g/mol

Chromatographic conditions: C18, 0.1% TFA in H_2O/ACN

TOF-MS: C18, acidic

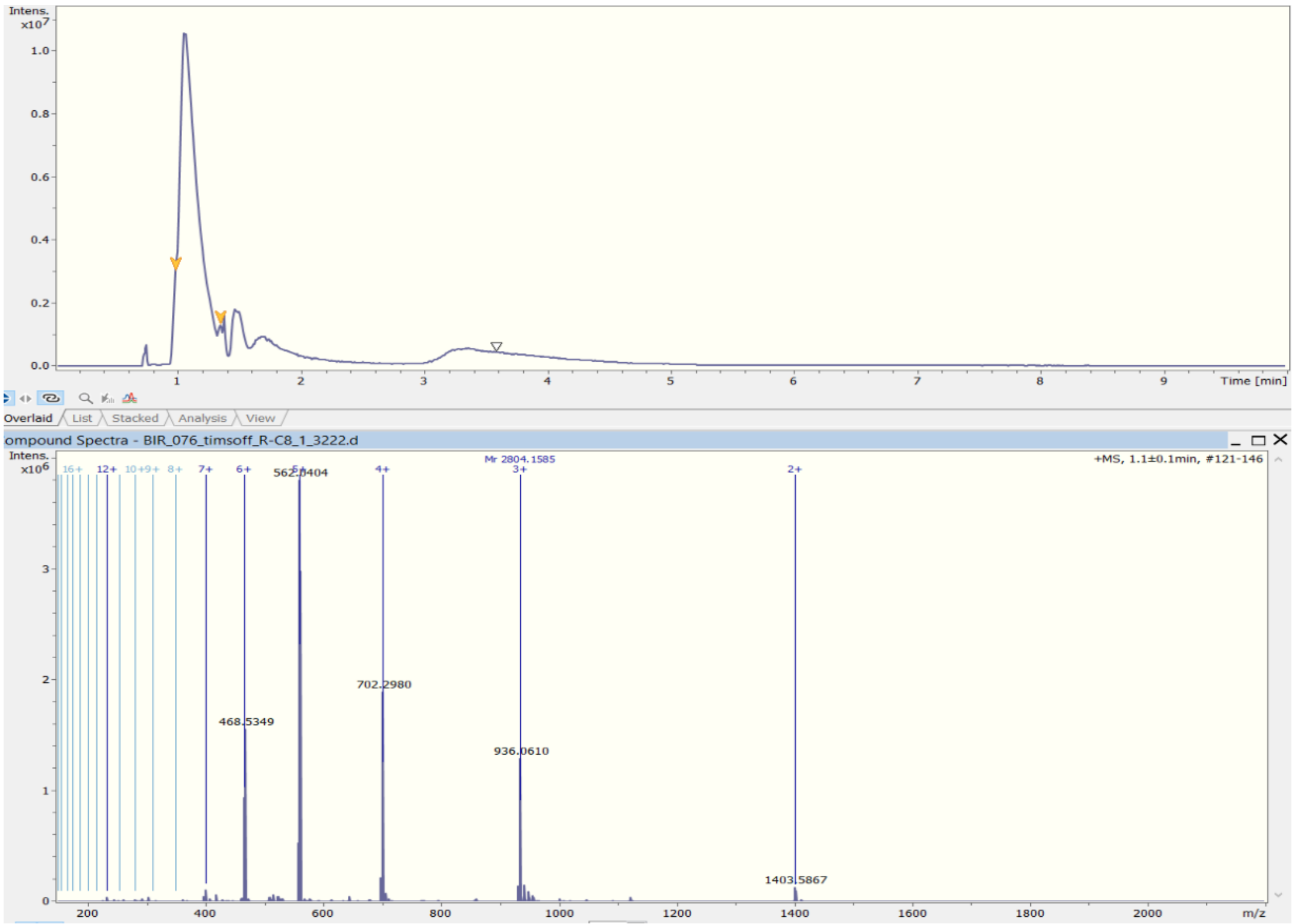

LC-MS: C18, acidic

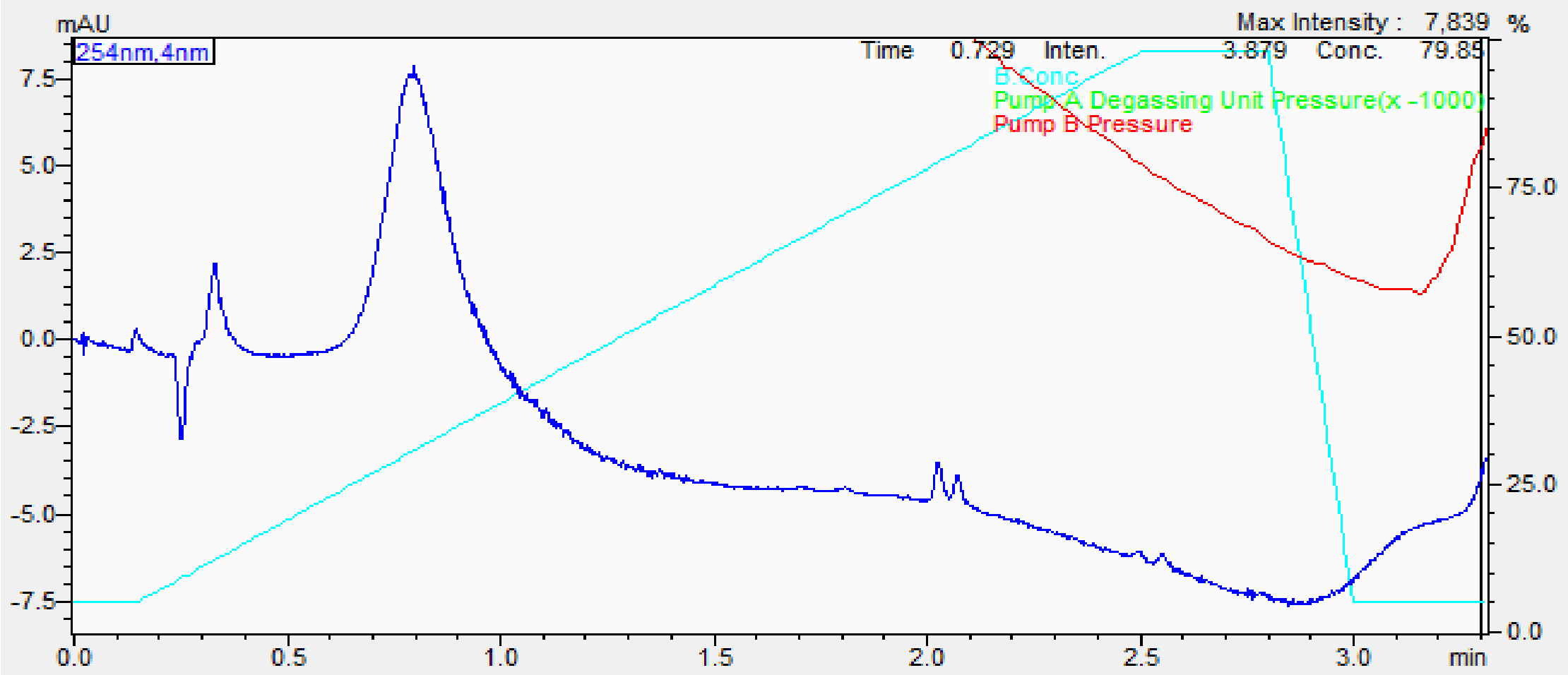
mAU
Max Intensity : 7,839
%
254nm,4nm
Time 0.729 Inten. 3.879 Conc. 79.85
B Conc.
Pump A Degassing Unit Pressure(x -1000)
Pump B Pressure
7.5
5.0
2.5
0.0
-2.5
-5.0
-7.5
75.0
50.0
25.0
0.0
0.0
0.5
1.0
1.5
2.0
2.5
3.0
min

3.3. 12mer PNA

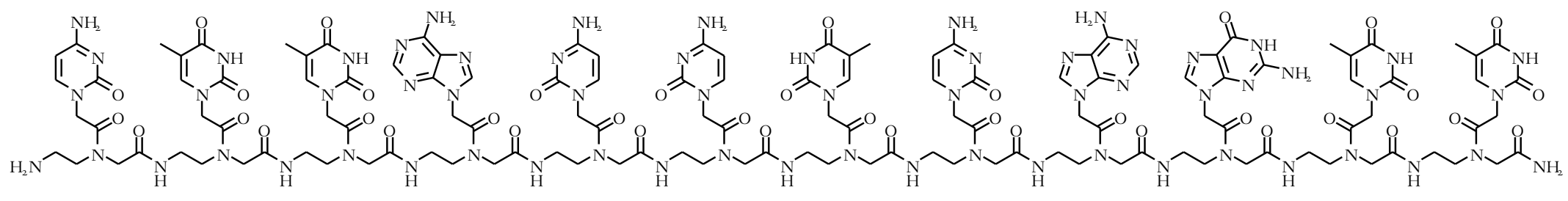

H-CTT ACC TCA GTT-NH$_2$

Exact Mass: 3193.28

Sequence: CTT ACC TCA GTT

Calculated mass: 3193.28 g/mol

Masses found: 1065.7693 $[M+3H]^{3+}$, 799.5793 $[M+4H]^{4+}$, 639.8651 $[M+5H]^{5+}$

□ 3194.2878 g/mol

Chromatographic conditions: C8, 0.1% TFA in H_2O/ACN

TOF-MS: C18, acidic

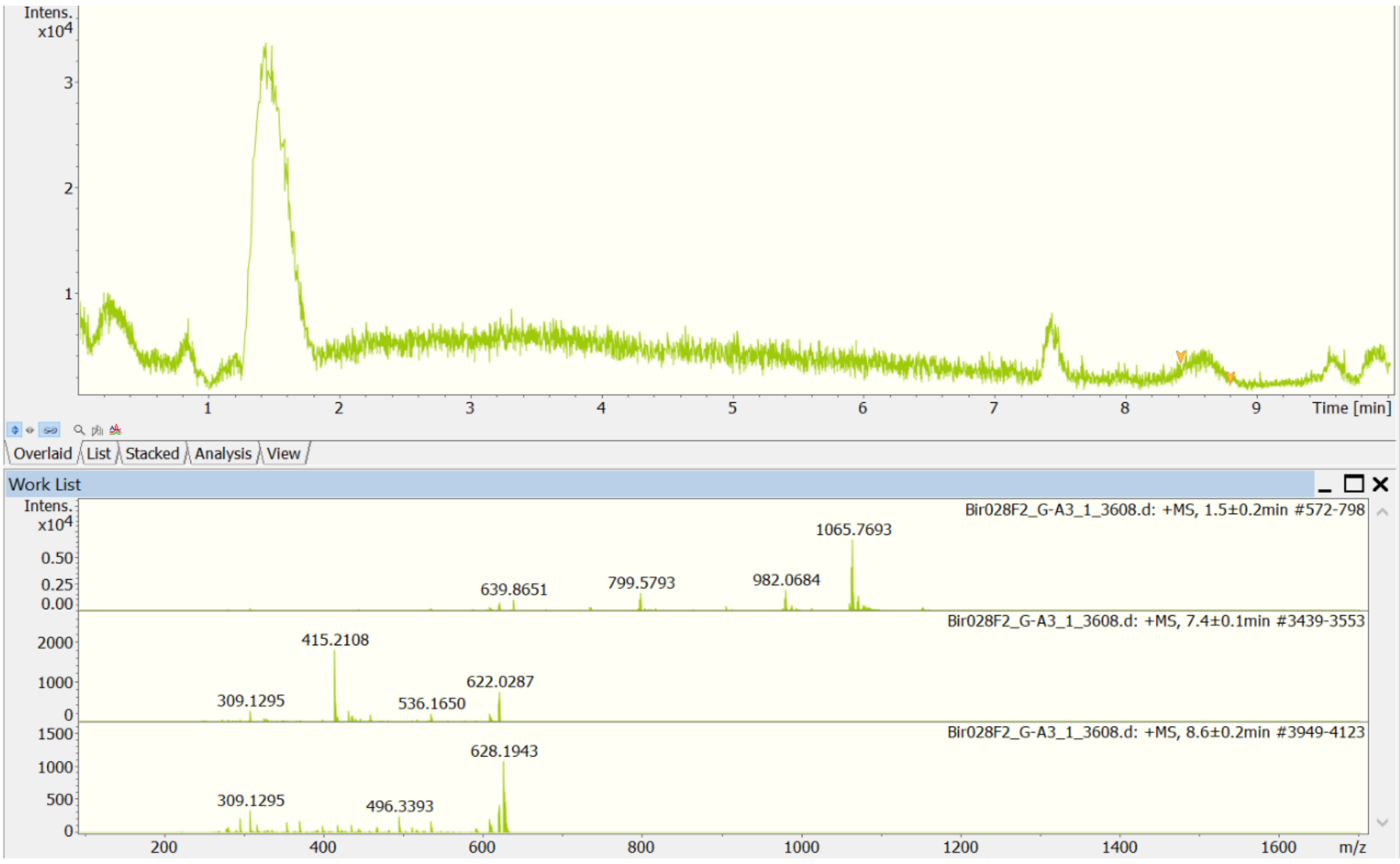

LC-MS: C18, acidic

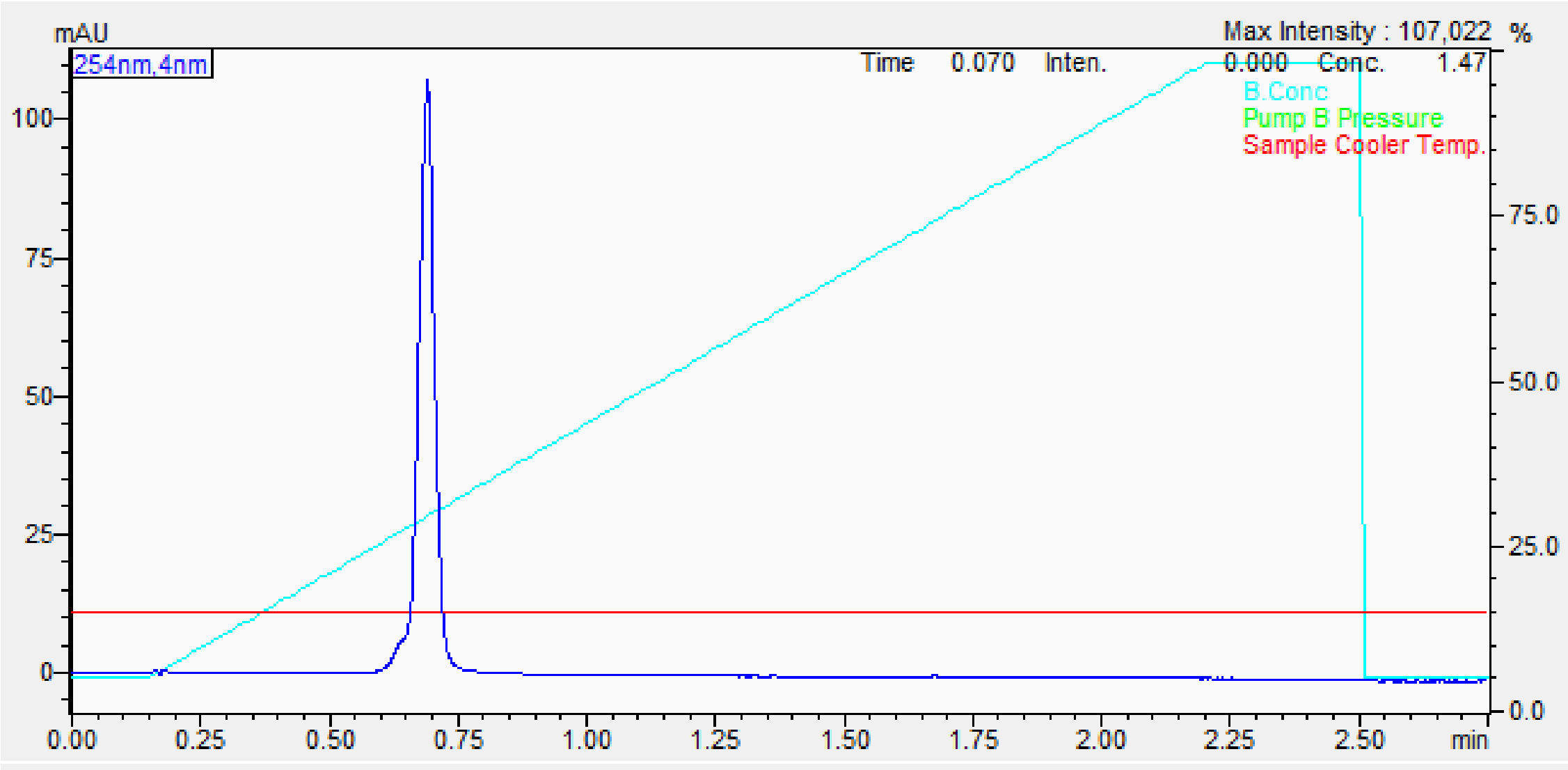
mAU
Max Intensity : 107,022
%
254nm,4nm
Time 0.070 Inten. 0.000 Conc. 1.47
B.Conc
Pump B Pressure
Sample Cooler Temp.
100
75
50
25
0
75.0
50.0
25.0
0.0
0.00
0.25
0.50
0.75
1.00
1.25
1.50
1.75
2.00
2.25
2.50
min

3.4. 14mer PNA

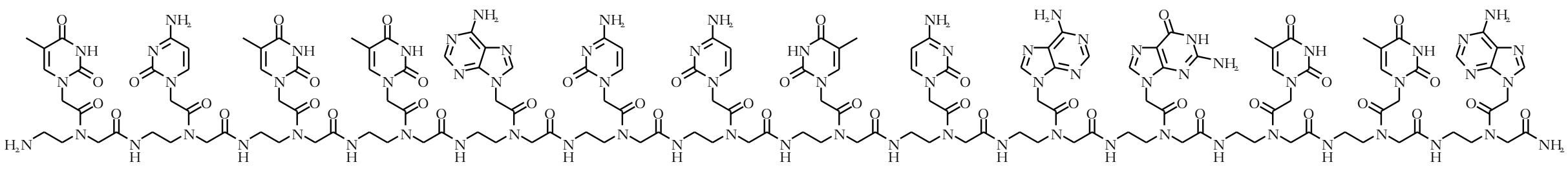

H-TCTTACCTCAGTTA-NH_2

Exact Mass: 3734.49

Sequence: TCA TAC CTC AGT TA

Calculated mass: 3734.49 g/mol

Masses found: 935.1377 $[M+4H]^{4+}$, 748.3104 $[M+5H]^{5+}$, 623.5923 $[M+6H]^{6+}$

□ 3734.5112 g/mol

Chromatographic conditions: C18, 0.1% TFA in H_2O/ACN

TOF-MS: C18, acidic

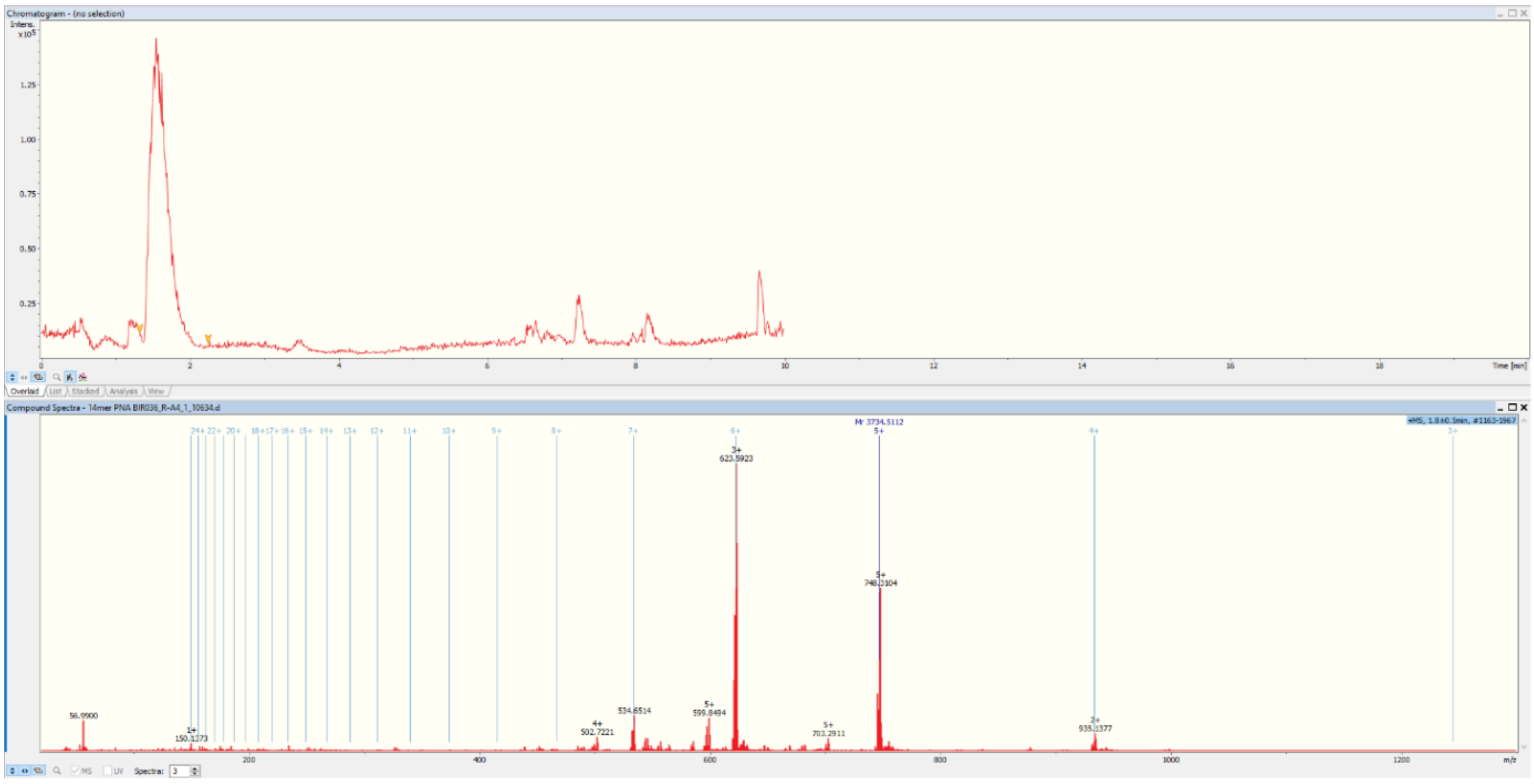

LC-MS: C18, acidic

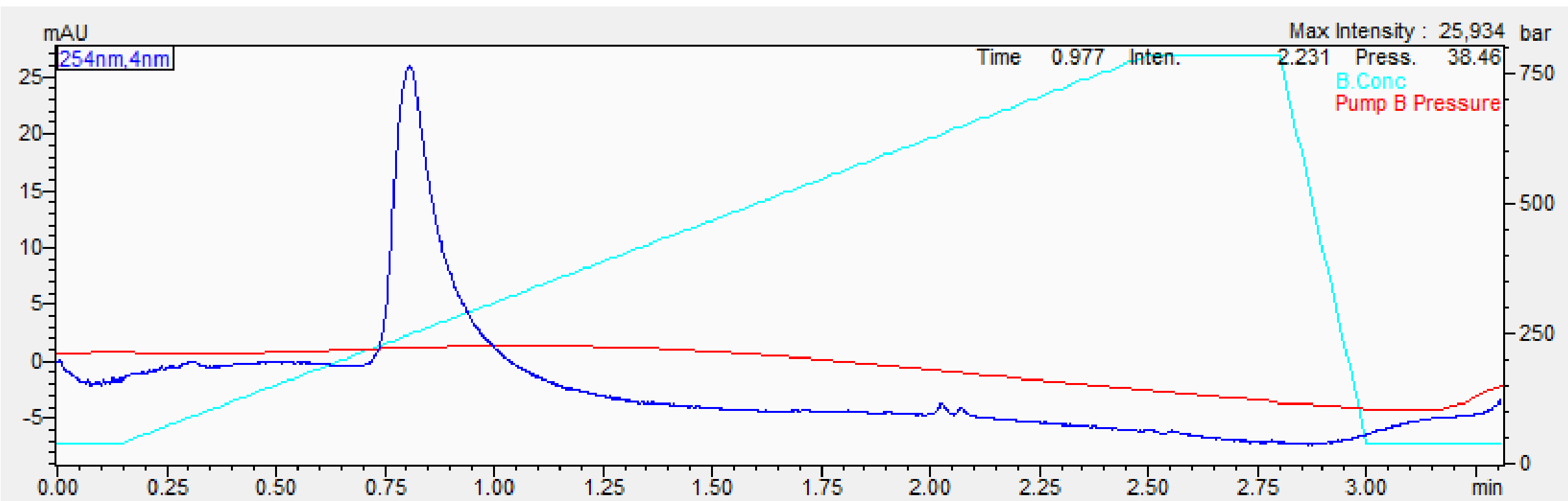

3.5. 10mer mp-PNA

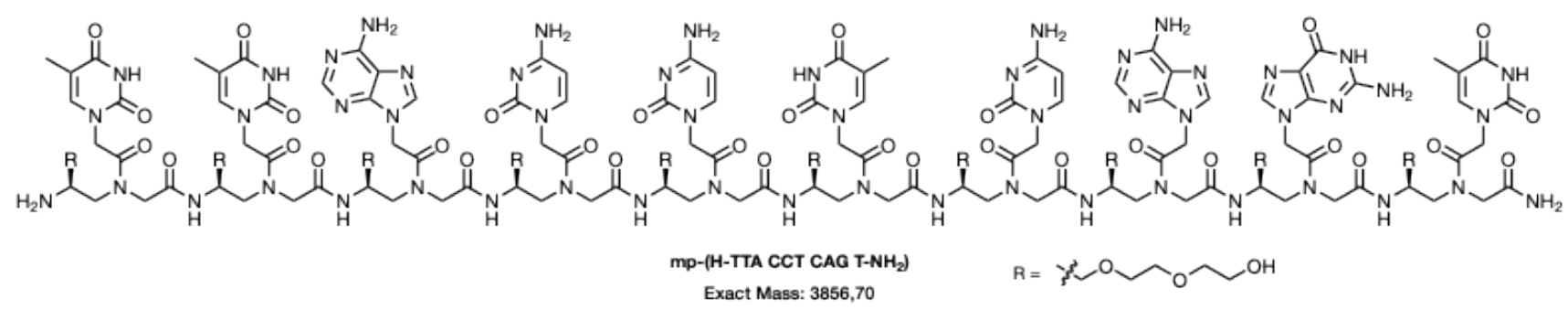

Sequence: mp-TTA CCT CAG T

Calculated mass: 3856.70 g/mol

Masses found: 965.7083 $[M+4H]^{4+}$, 772.7661 $[M+5H]^{5+}$, 644.1377 $[M+6H]^{6+}$

□ 3856.7771 g/mol

Chromatographic conditions: HILIC, basic (pH 8.5), 80-60% ACN in H_2O

TOF-MS: C18, acidic

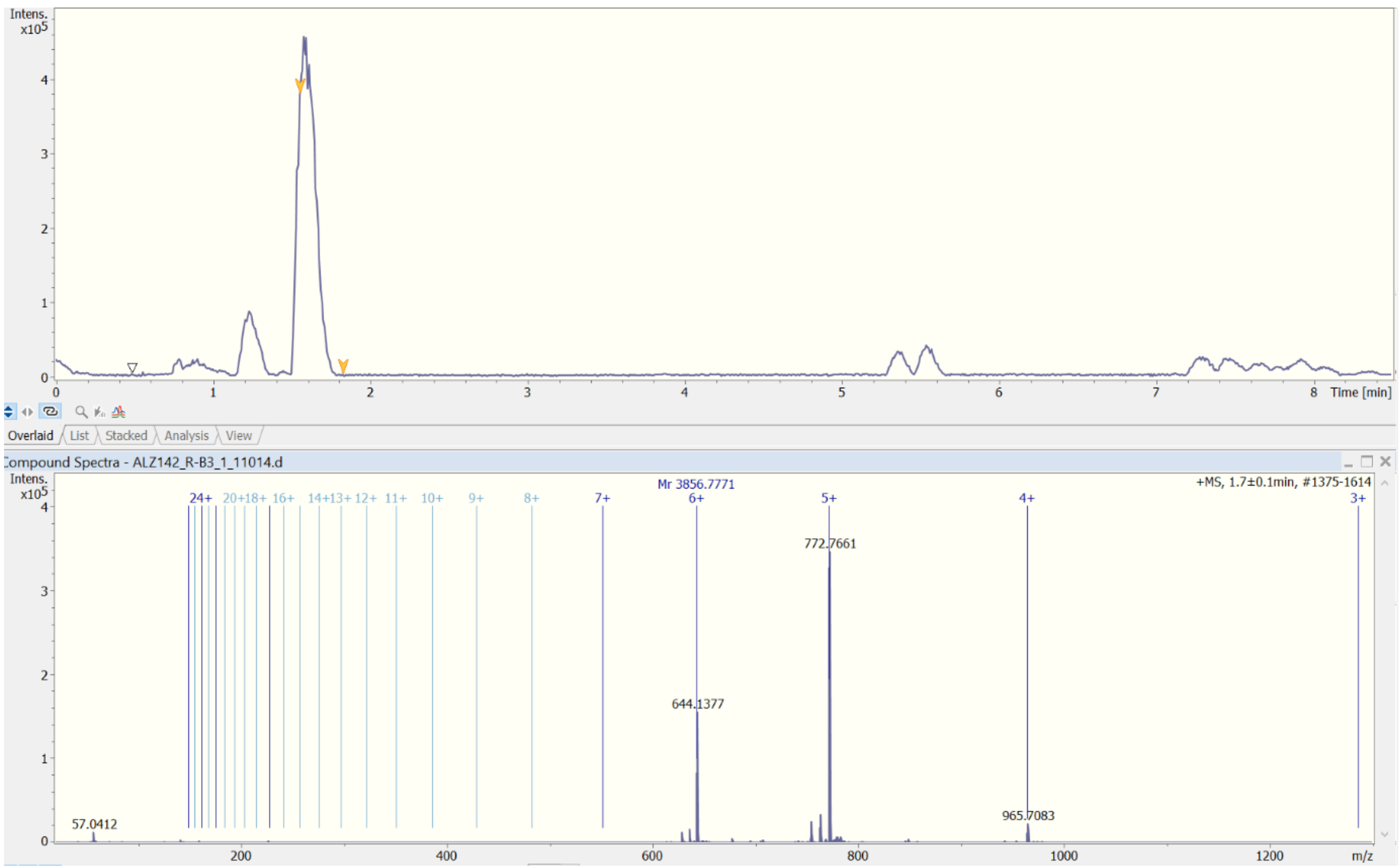

LC-MS: HILIC, basic (8.5)

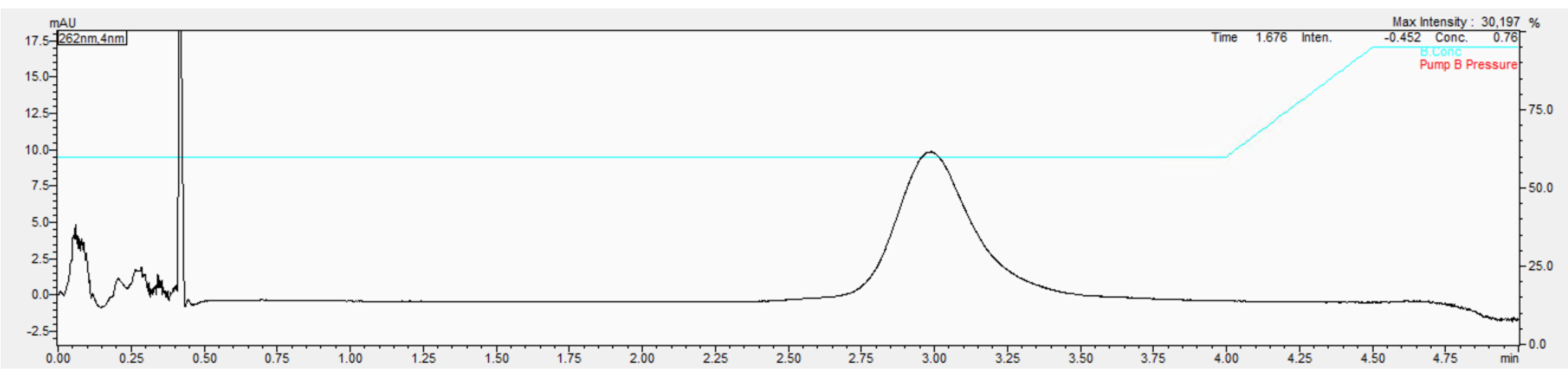

3.6. 12mer mp-PNA

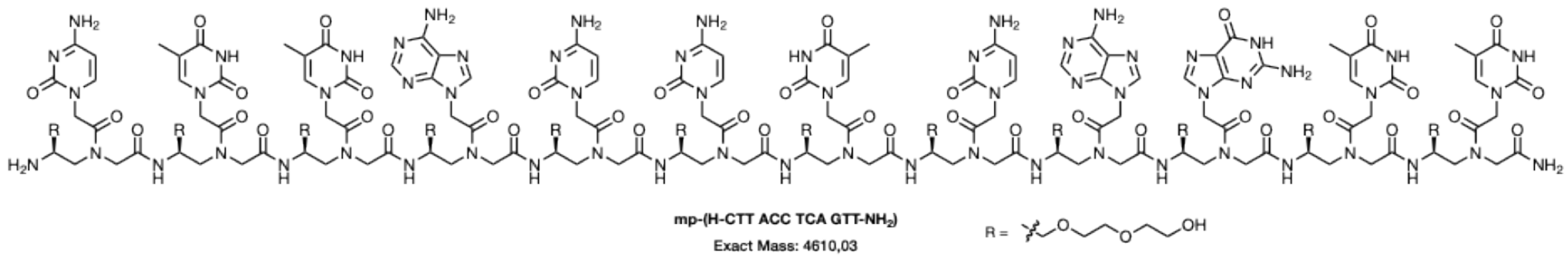

Sequence: mp-CTT ACC TCA GTT

Calculated mass: 4610.03 g/mol

Masses found: 1538.0162 $[M+3H]^{3+}$, 1154.0153 $[M+4H]^{4+}$, 923.4135 $[M+5H]^{5+}$

□ 4610.0286 g/mol

Chromatographic conditions: HILIC, basic (pH 8.5), 90-60% ACN in H_2O

TOF-MS: C18, acidic

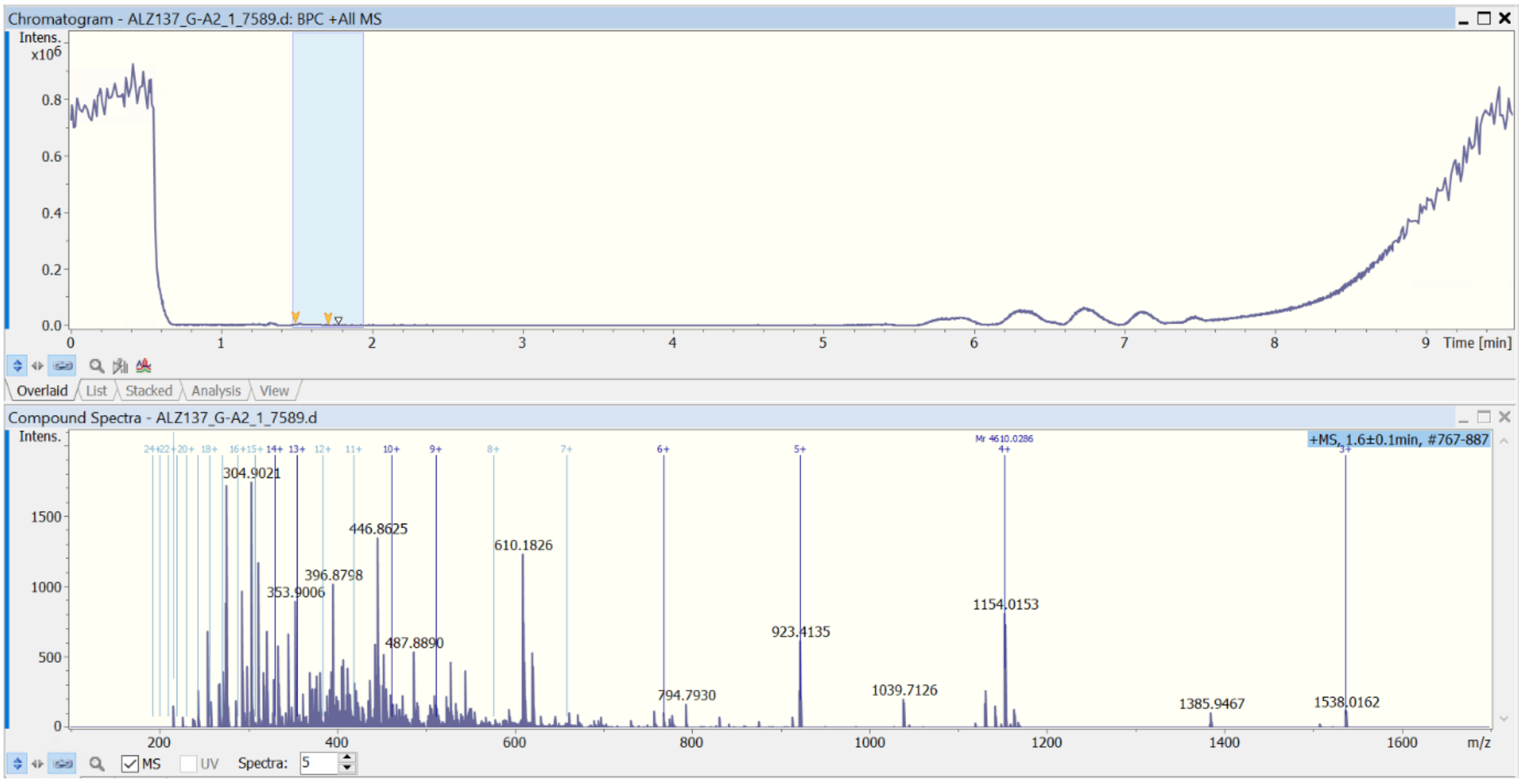

LC-MS: C18, acidic

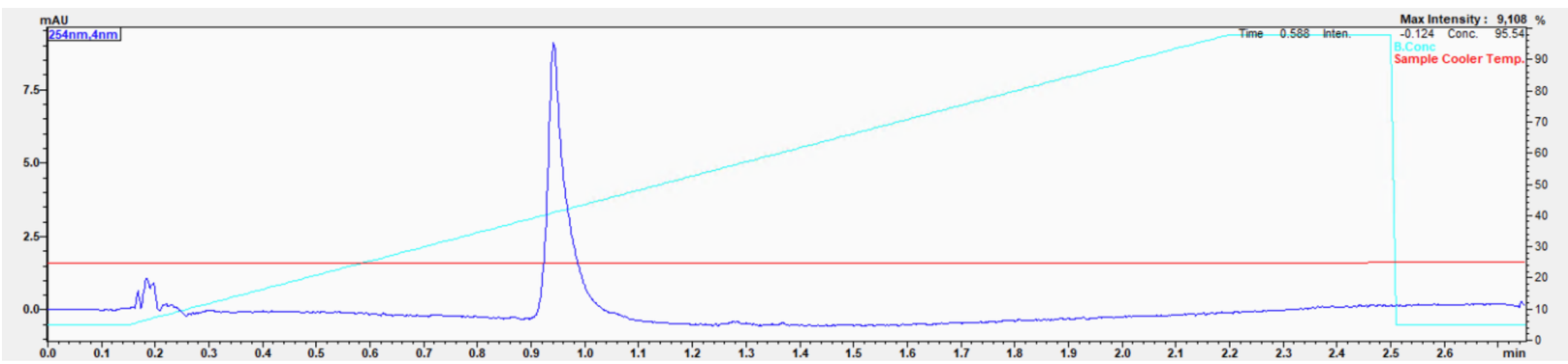

3.7. 14mer mp-PNA

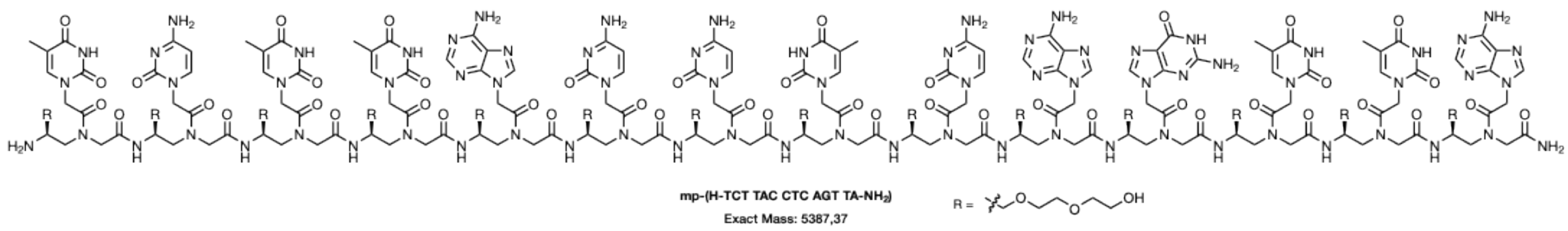

Sequence: mp-TCT TAC CTC AGT TA

Calculated mass: 5387.37 g/mol

Masses found: 1078.9088 $[M+5H]^{5+}$, 899.2585 $[M+6H]^{6+}$, 771.0793 $[M+7H]^{7+}$

□ 5387.4962 g/mol

Chromatographic conditions: HILIC, basic (pH 8.5), 90-45% ACN in H_2O

TOF-MS: C18, acidic

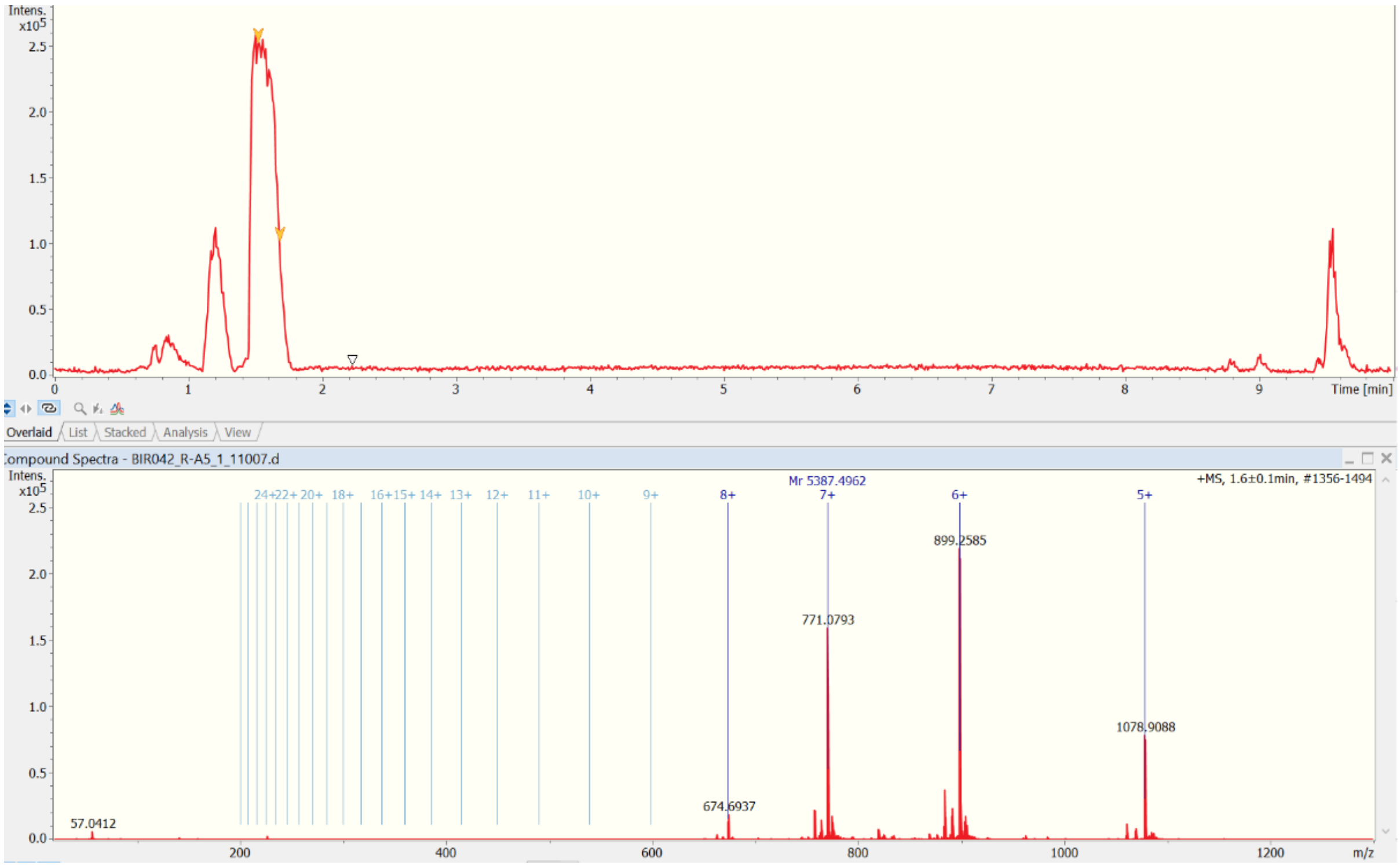

LC-MS: C18, basic

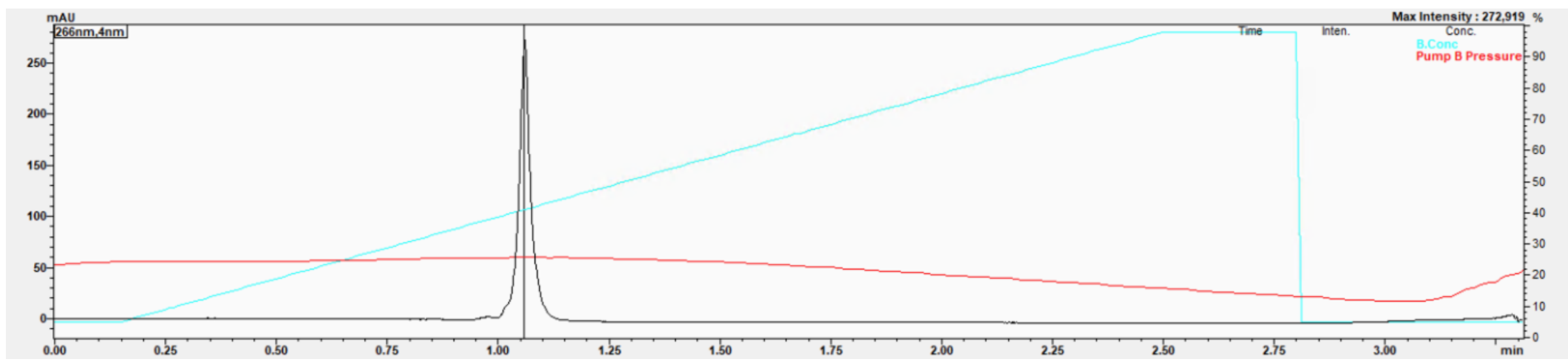

4. SPR Kinetic Data of RNAs, PNAs and mp-PNAs

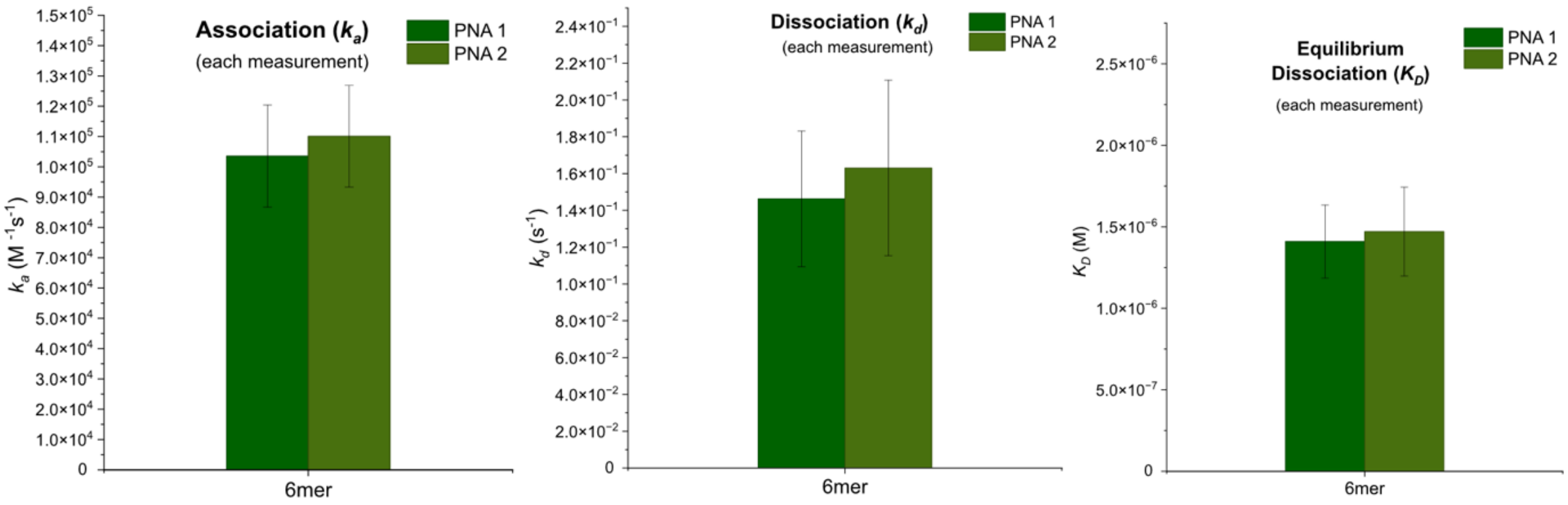

SI Figure 1: Association (k_a), dissociation (k_d) and equilibrium dissociation (K_D) rate constants for each measurement of 6mer PNA hybridization measured via SPR (n = 4).

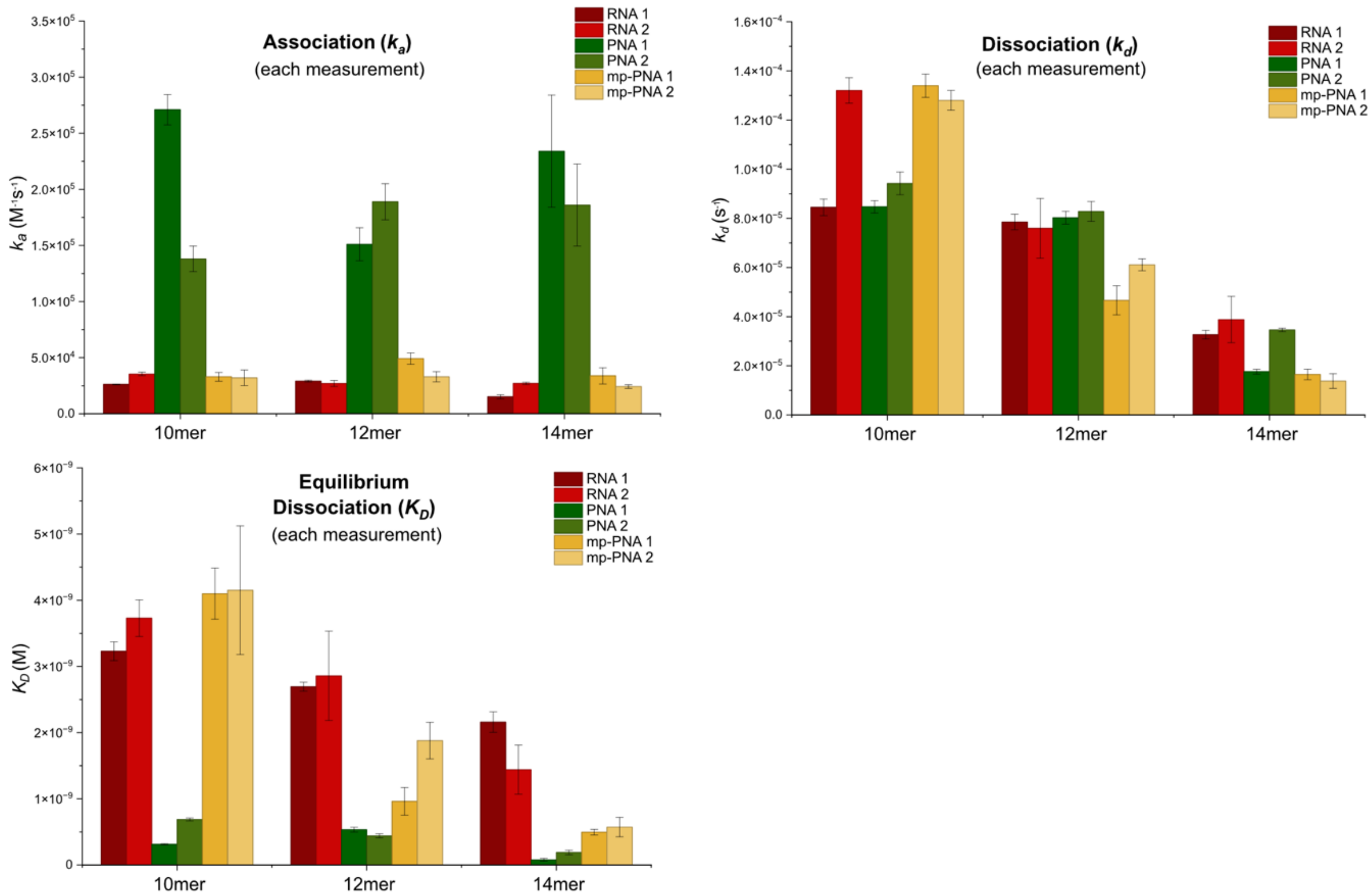

SI Figure 2: Association (k_a), dissociation (k_d) and equilibrium dissociation (K_D) rate constants for each measurement of 10, 12 and 14mer RNA, PNA and mp-PNA hybridization measured via SPR (n = 4).

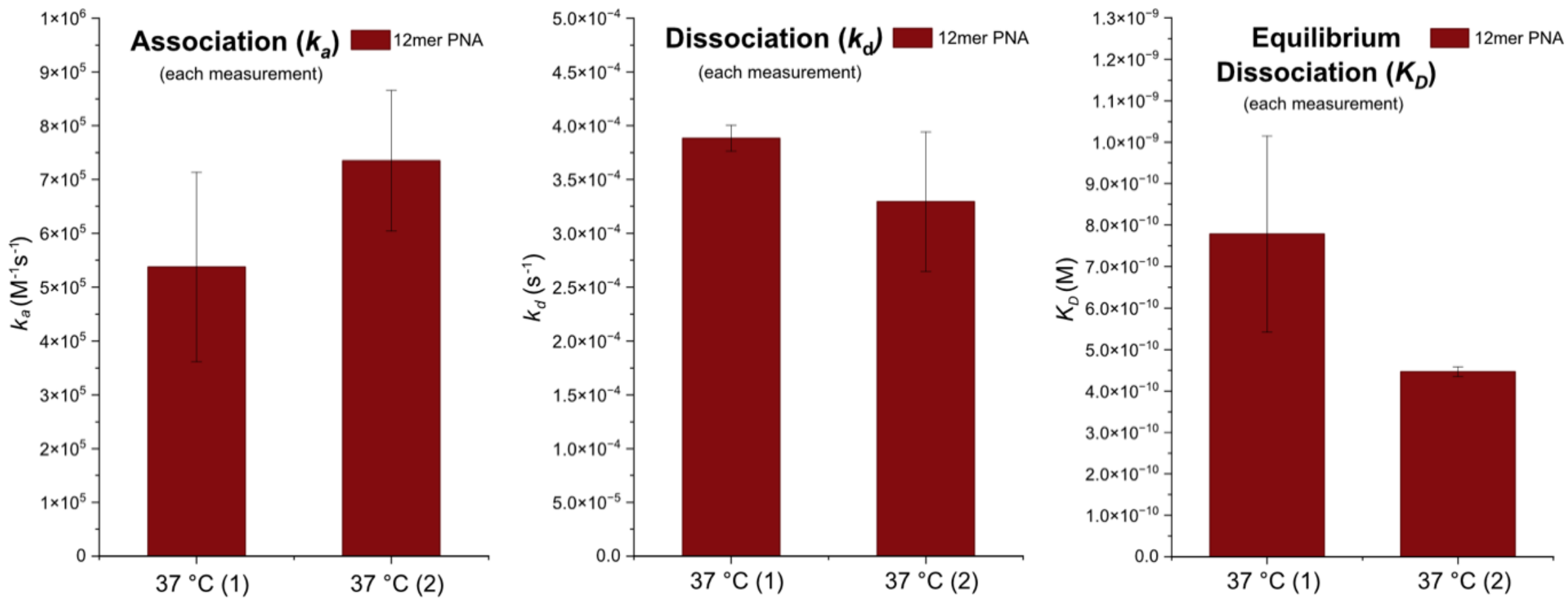

SI Figure 3: Association (k_a), dissociation (k_d) and equilibrium dissociation (K_D) rate constants for each measurement of 12mer PNA hybridization at 37 °C measured via SPR (n = 3-4).

Table 2: Association (k_a), dissociation (k_d) and equilibrium dissociation (K_D) rate constants for each measurement of 6, 10, 12 and 14mer RNA, PNA and mp-PNA hybridization measured via SPR (n =2-4).

		RNA 1	SD (±)	RNA 2	SD (±)	PNA 1	SD (±)	PNA 2	SD (±)	mp-PNA 1	SD (±)	mp-PNA 2	SD (±)
k_a ($M^{-1}s^{-1}$)	6mer					1.04×10^5	1.69×10^4	1.10×10^5	1.68×10^4				
	10mer	2.62×10^4	2.59×10^2	3.54×10^4	1.60×10^3	2.71×10^5	1.35×10^4	1.38×10^5	1.14×10^4	3.30×10^4	3.87×10^3	3.20×10^4	6.96×10^3
	12mer	2.91×10^4	7.94×10^2	2.70×10^4	2.71×10^3	1.51×10^5	1.46×10^4	1.89×10^5	1.60×10^4	4.92×10^4	5.13×10^3	3.30×10^4	4.49×10^3
	12mer 37 °C					5.37×10^5	1.76×10^5	7.35×10^5	1.31×10^5				
	14mer	1.52×10^4	1.81×10^3	2.71×10^4	9.98×10^2	2.34×10^5	5.01×10^4	1.86×10^5	3.65×10^4	3.38×10^4	7.17×10^3	2.43×10^4	1.68×10^3
k_d (s^{-1})	6mer					1.46×10^{-1}	3.69×10^{-2}	1.63×10^{-1}	4.78×10^{-2}				
	10mer	8.45×10^{-5}	3.35×10^{-6}	1.32×10^{-4}	5.18×10^{-6}	8.47×10^{-5}	2.53×10^{-6}	9.42×10^{-5}	4.60×10^{-6}	1.34×10^{-4}	4.71×10^{-6}	1.28×10^{-4}	4.03×10^{-6}
	12mer	7.85×10^{-5}	3.11×10^{-6}	7.59×10^{-5}	1.22×10^{-5}	8.03×10^{-5}	2.61×10^{-6}	8.28×10^{-5}	4.04×10^{-6}	4.66×10^{-5}	5.93×10^{-6}	6.11×10^{-5}	2.40×10^{-6}
	12mer 37 °C					3.88×10^{-4}	1.20×10^{-5}	3.29×10^{-4}	6.49×10^{-5}				
	14mer	3.27×10^{-5}	1.79×10^{-6}	3.88×10^{-5}	9.41×10^{-6}	1.76×10^{-5}	9.44×10^{-7}	3.46×10^{-5}	6.65×10^{-7}	1.65×10^{-5}	2.13×10^{-6}	1.38×10^{-5}	2.97×10^{-6}
K_D (M)	6mer					1.41×10^{-6}	2.24×10^{-7}	1.47×10^{-6}	2.72×10^{-7}				
	10mer	3.23×10^{-9}	1.40×10^{-10}	3.73×10^{-9}	2.77×10^{-10}	3.13×10^{-10}	7.87×10^{-12}	6.87×10^{-10}	2.27×10^{-11}	4.10×10^{-9}	3.87×10^{-10}	4.15×10^{-9}	9.72×10^{-10}
	12mer	2.69×10^{-9}	6.63×10^{-11}	2.86×10^{-9}	6.75×10^{-10}	5.34×10^{-10}	3.41×10^{-11}	4.40×10^{-10}	3.07×10^{-11}	9.62×10^{-10}	2.10×10^{-10}	1.88×10^{-9}	2.77×10^{-10}
	12mer 37 °C					7.79×10^{-10}	2.36×10^{-10}	4.47×10^{-10}	1.17×10^{-11}				
	14mer	2.16×10^{-9}	1.53×10^{-10}	1.44×10^{-9}	3.70×10^{-10}	7.84×10^{-11}	1.87×10^{-11}	1.90×10^{-10}	3.37×10^{-11}	4.96×10^{-10}	4.19×10^{-11}	5.72×10^{-10}	1.45×10^{-10}

5. SPR Sensorgrams of RNAs, PNAs and mp-PNAs

5.1. 10mer RNA

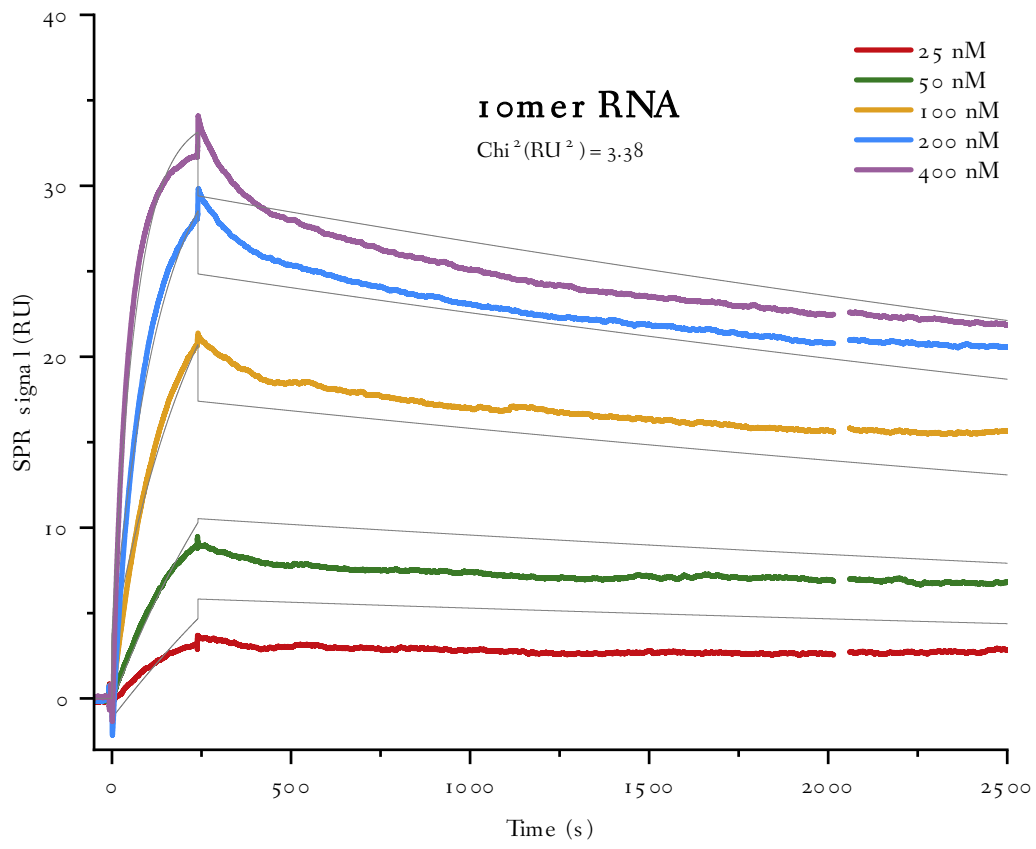

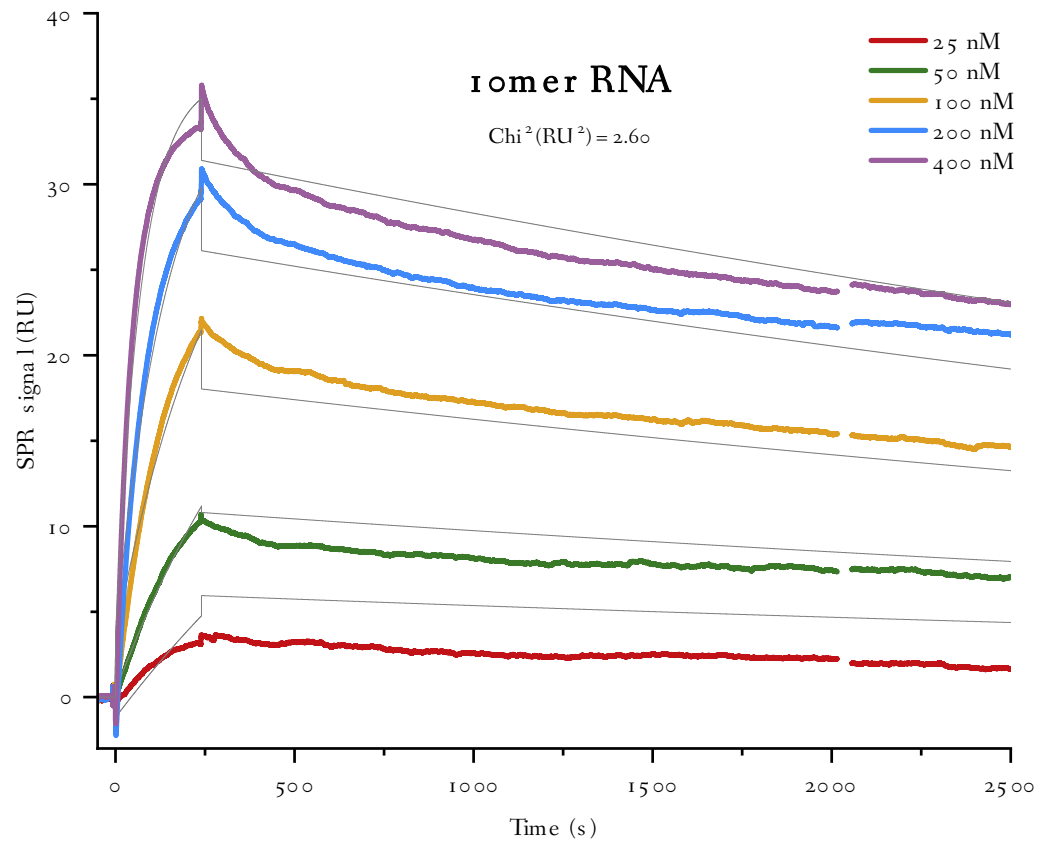

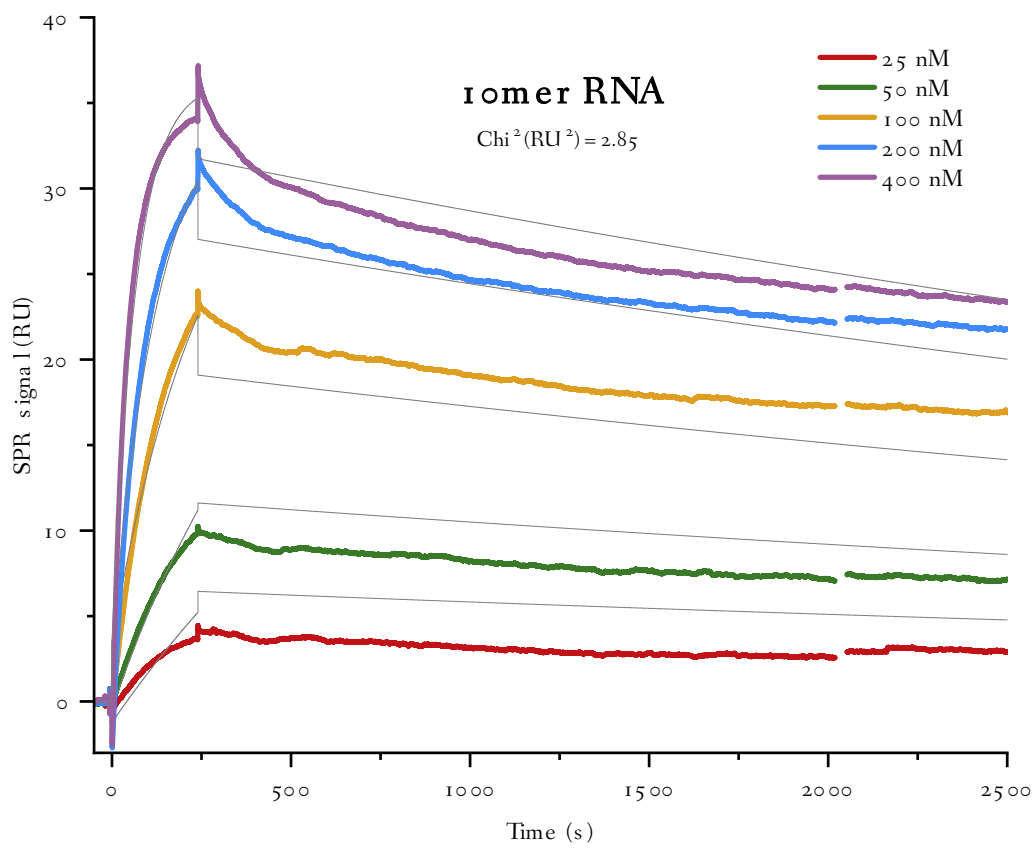

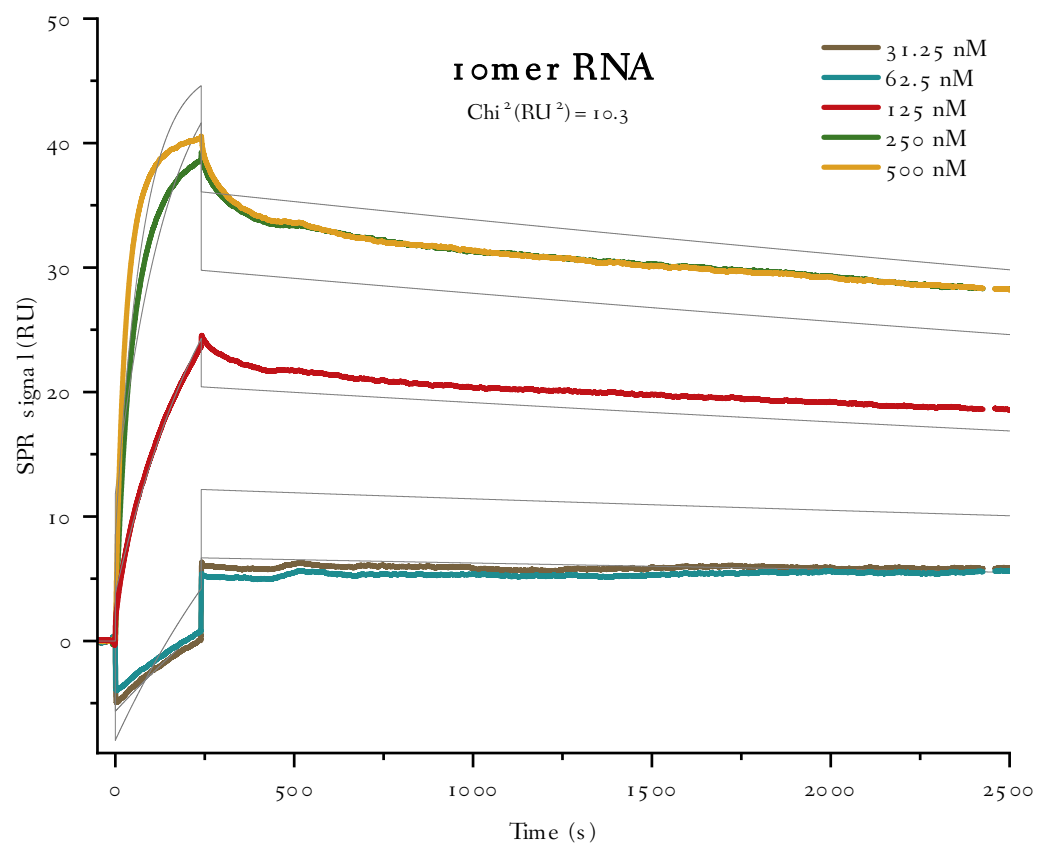

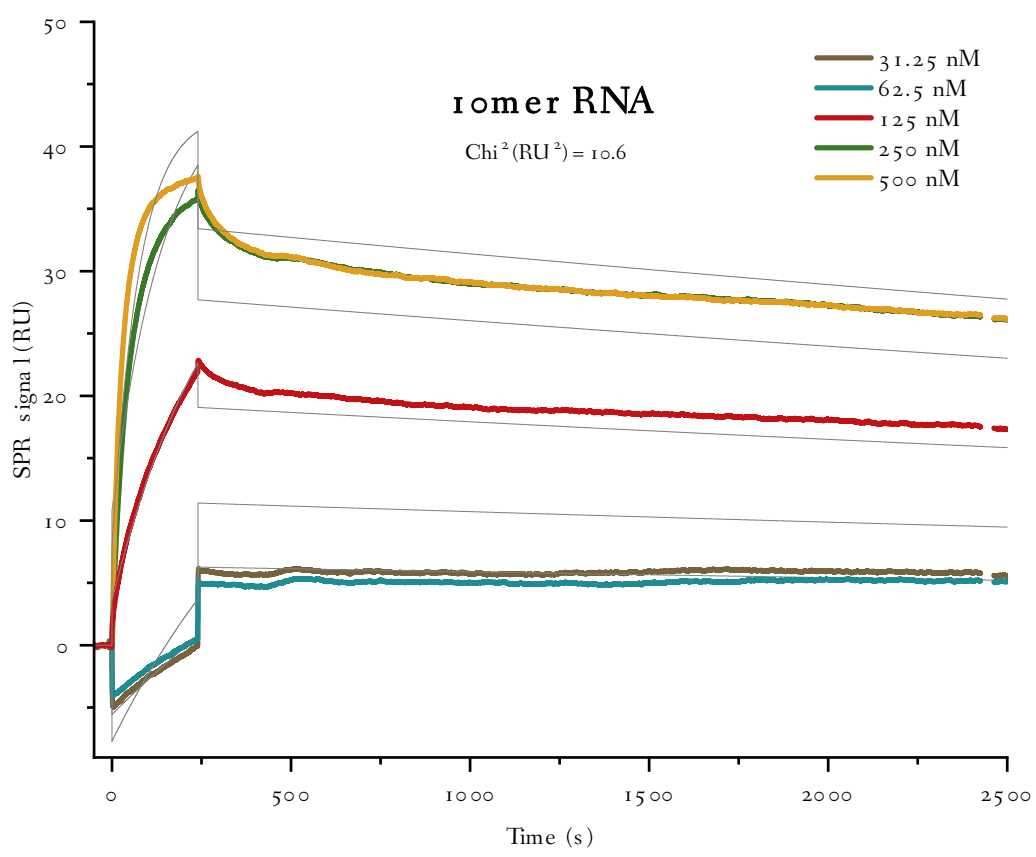

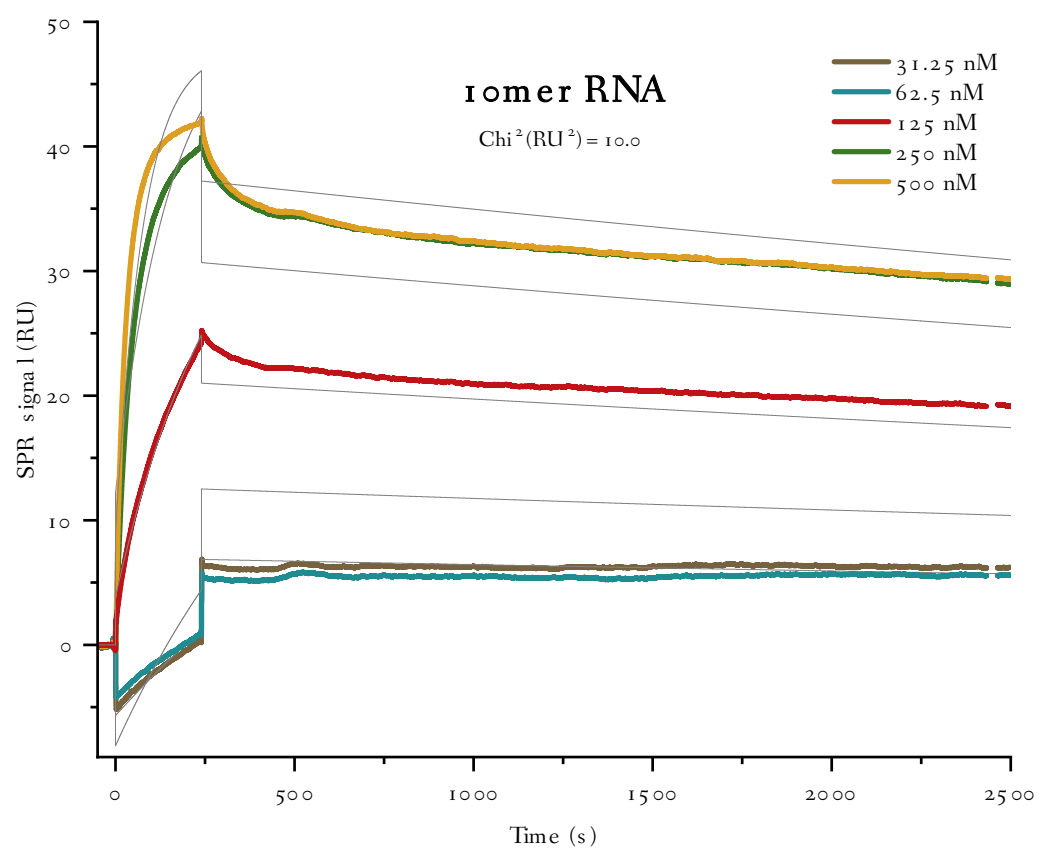

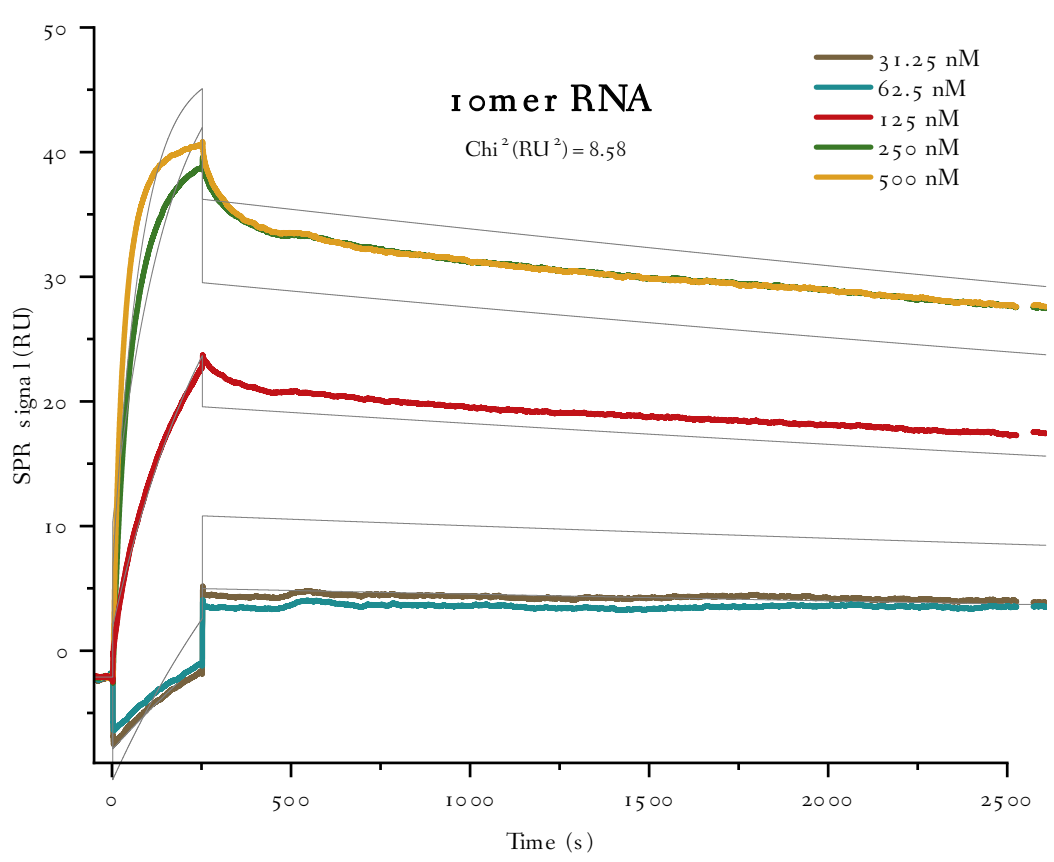

5.2. 12mer RNA

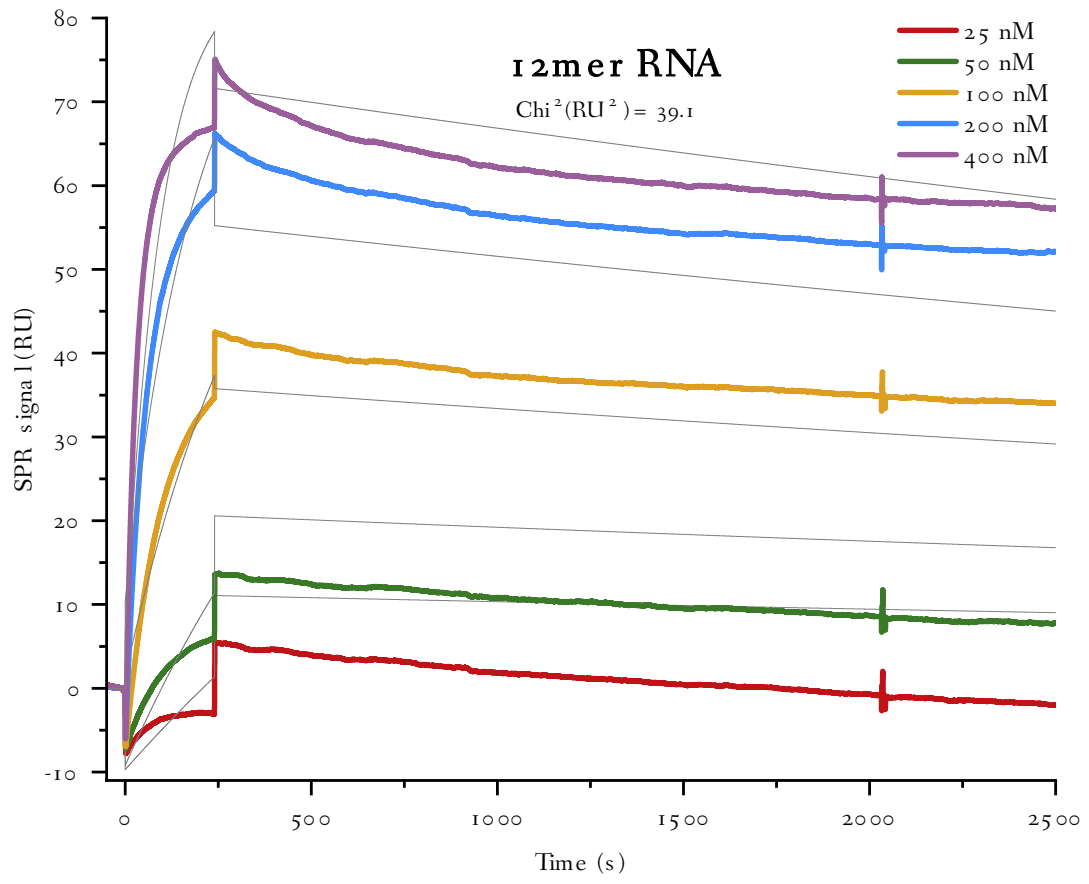

12mer RNA
Chi^2(RU^2) = 39.1
25 nM
50 nM
100 nM
200 nM
400 nM
SPR signal (RU)
Time (s)

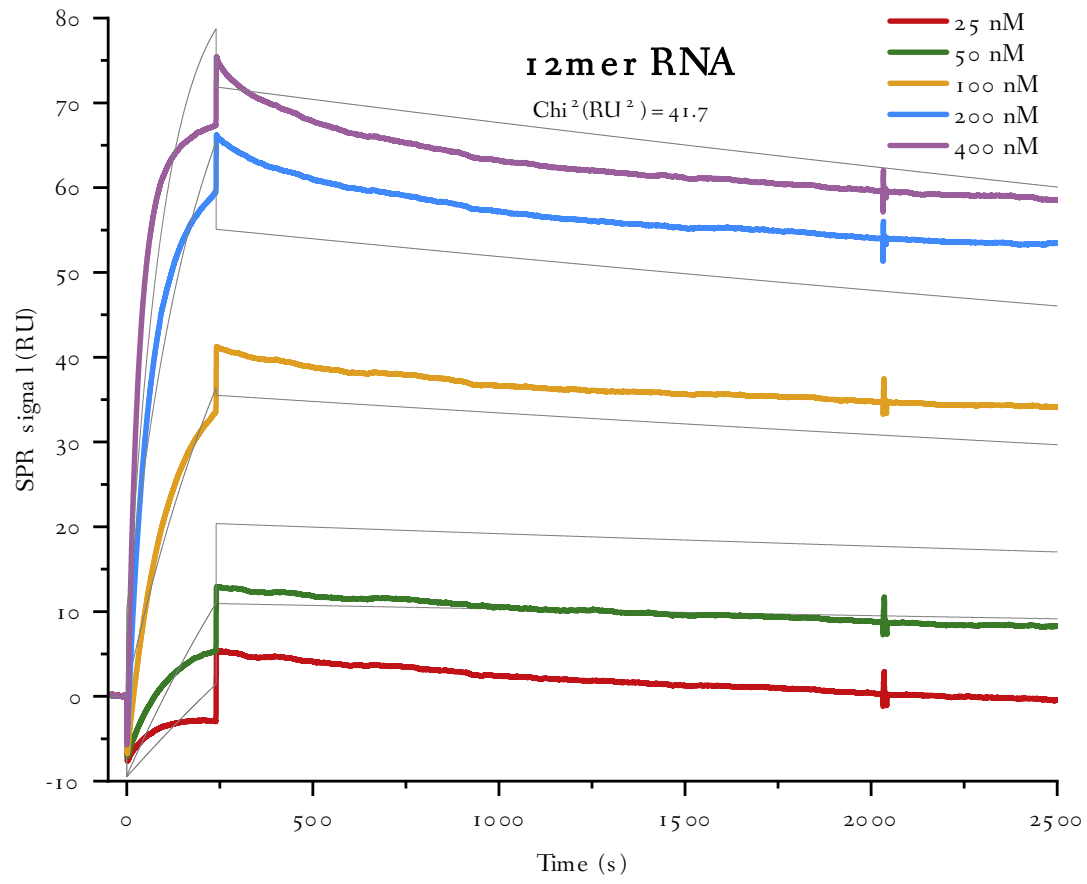

12mer RNA
Chi^2(RU^2) = 41.7
25 nM
50 nM
100 nM
200 nM
400 nM
SPR signal (RU)
Time (s)

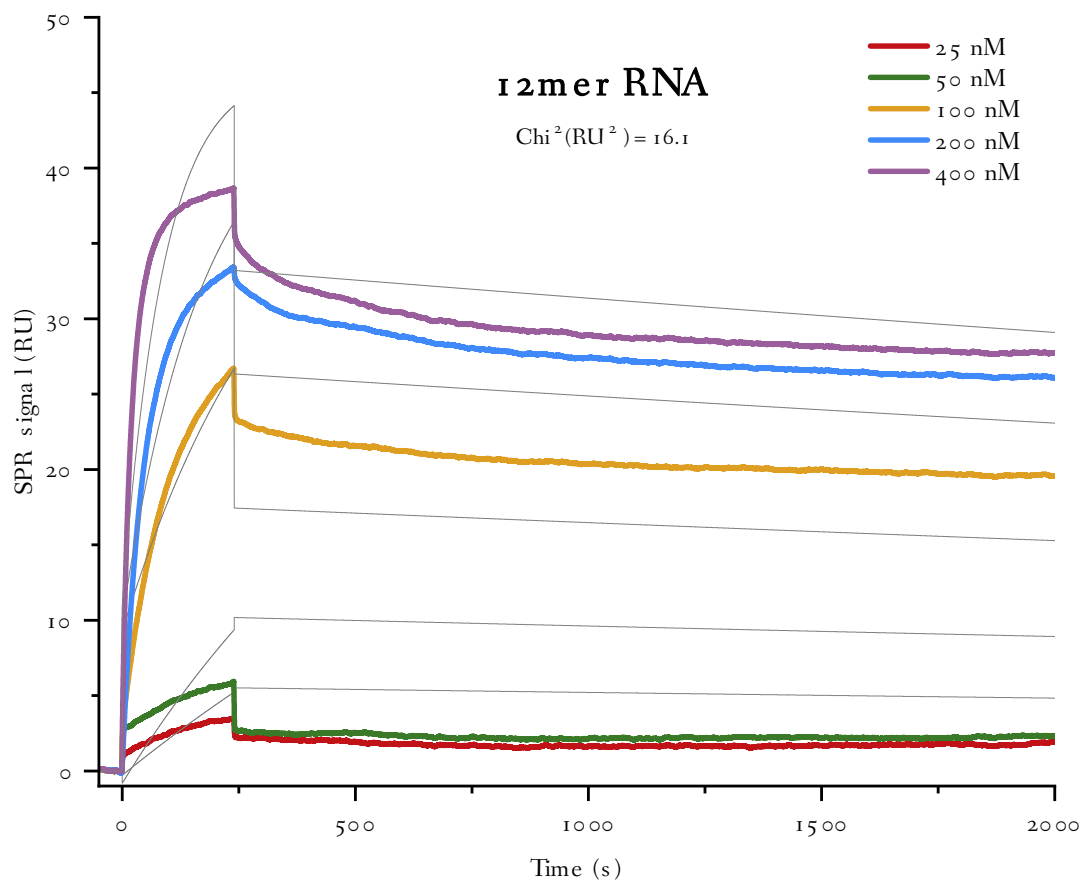

12mer RNA
Chi^2(RU^2) = 16.1
25 nM
50 nM
100 nM
200 nM
400 nM
SPR signal (RU)
Time (s)

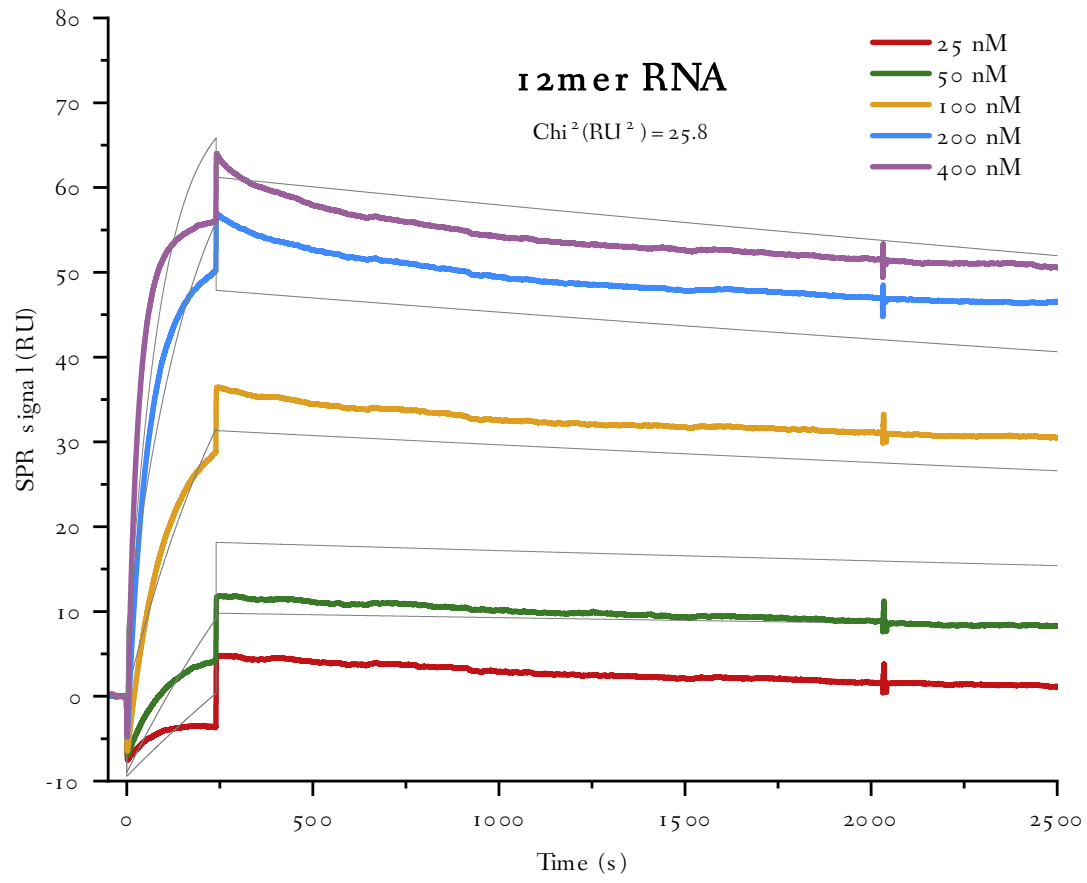

12mer RNA
Chi^2(RU^2) = 25.8
25 nM
50 nM
100 nM
200 nM
400 nM
SPR signal (RU)
Time (s)

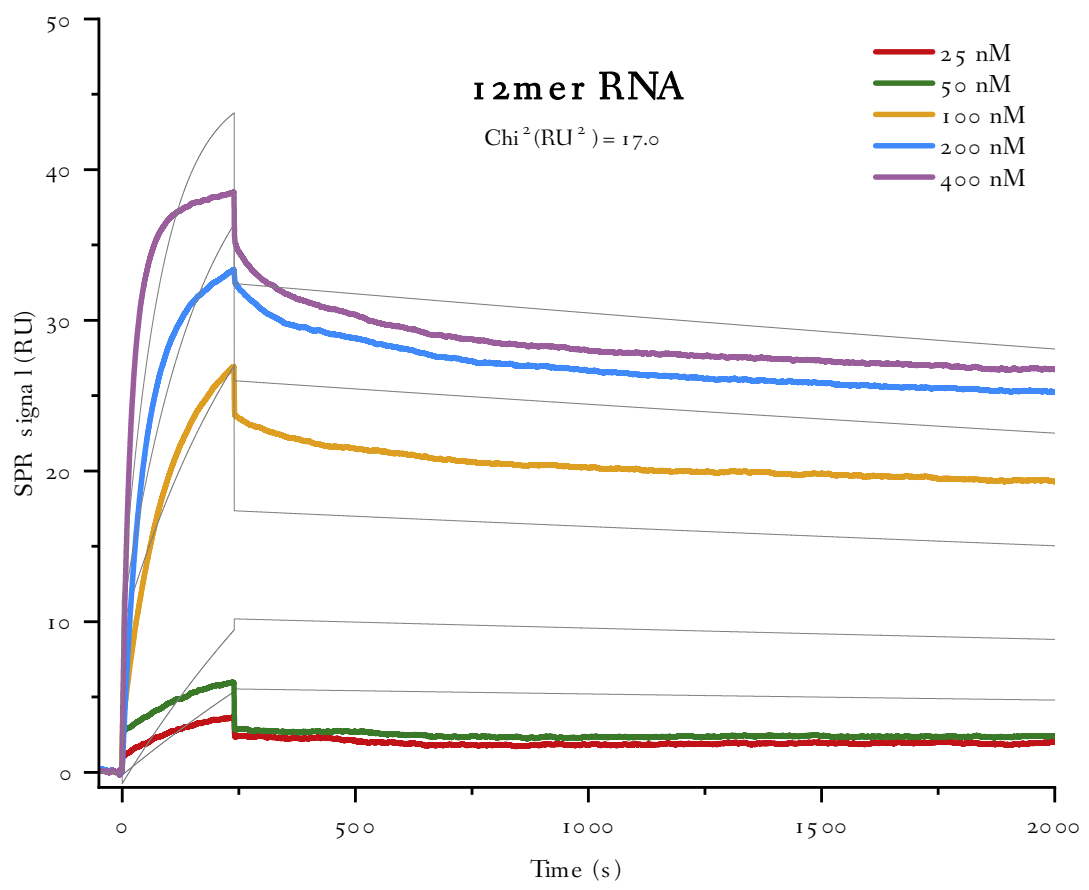

12mer RNA
Chi^2(RU^2) = 17.0
25 nM
50 nM
100 nM
200 nM
400 nM
SPR signal (RU)
Time (s)

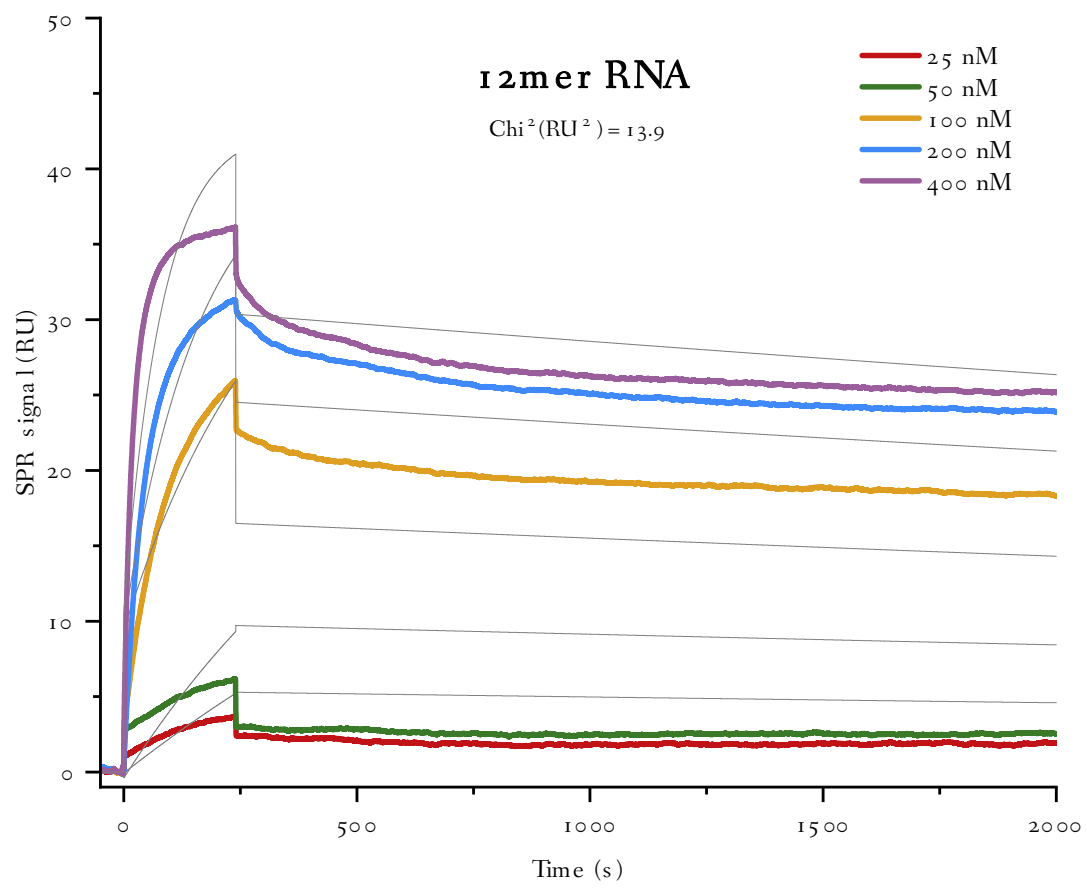

12mer RNA
Chi^2(RU^2) = 13.9
25 nM
50 nM
100 nM
200 nM
400 nM
SPR signal (RU)
Time (s)

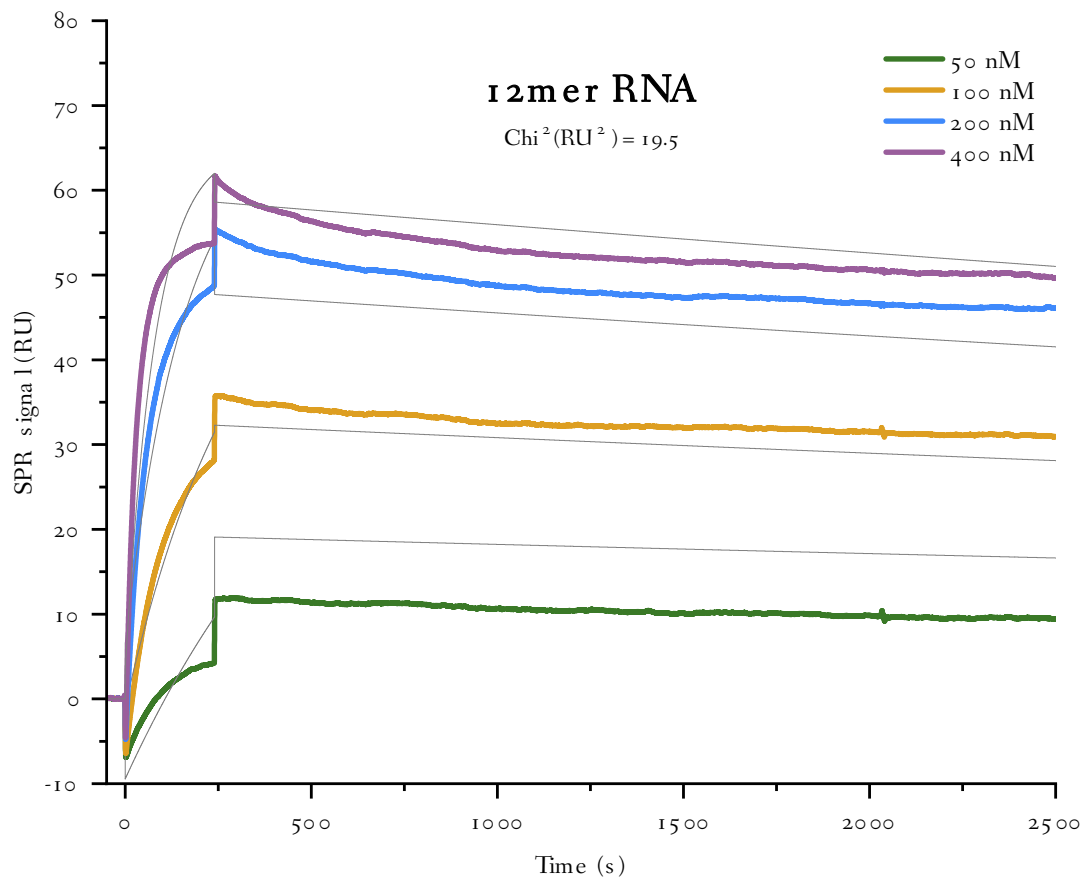

12mer RNA
Chi^2(RU^2) = 19.5
50 nM
100 nM
200 nM
400 nM
SPR signal (RU)
Time (s)

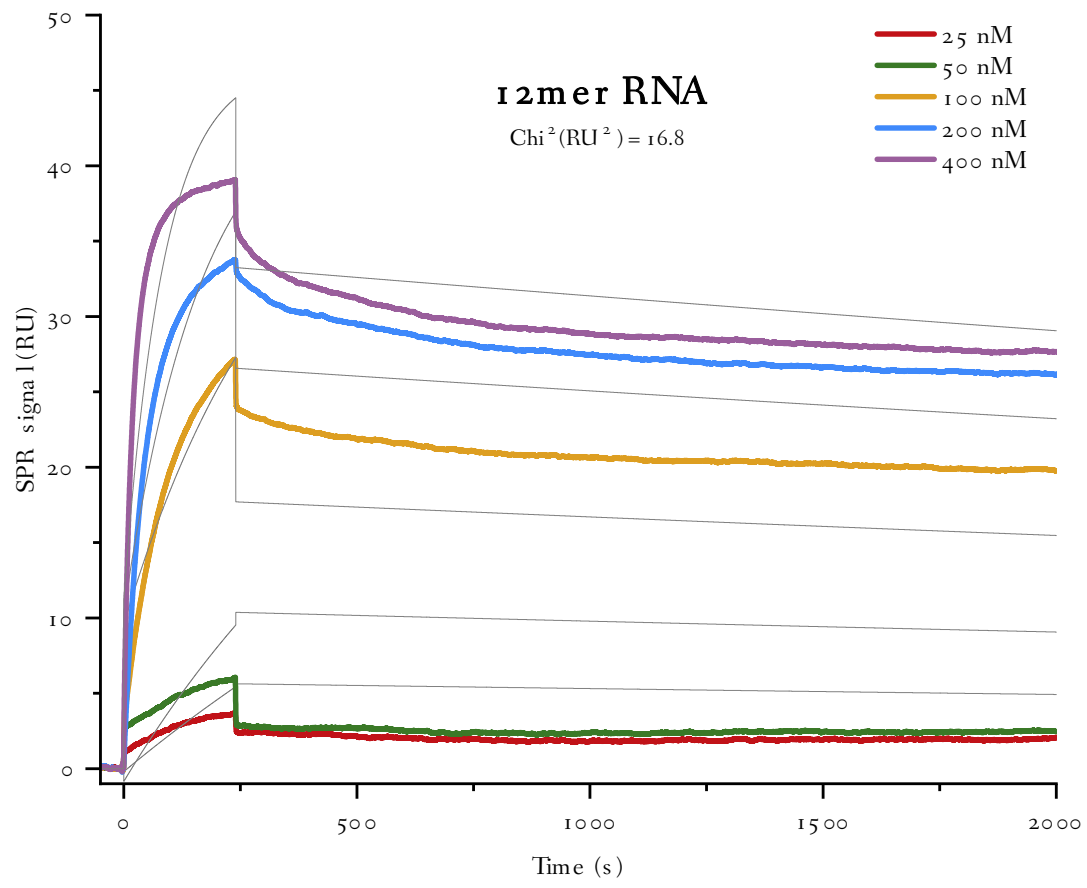

12mer RNA
Chi^2(RU^2) = 16.8
25 nM
50 nM
100 nM
200 nM
400 nM
SPR signal (RU)
Time (s)

5.3. 14mer RNA

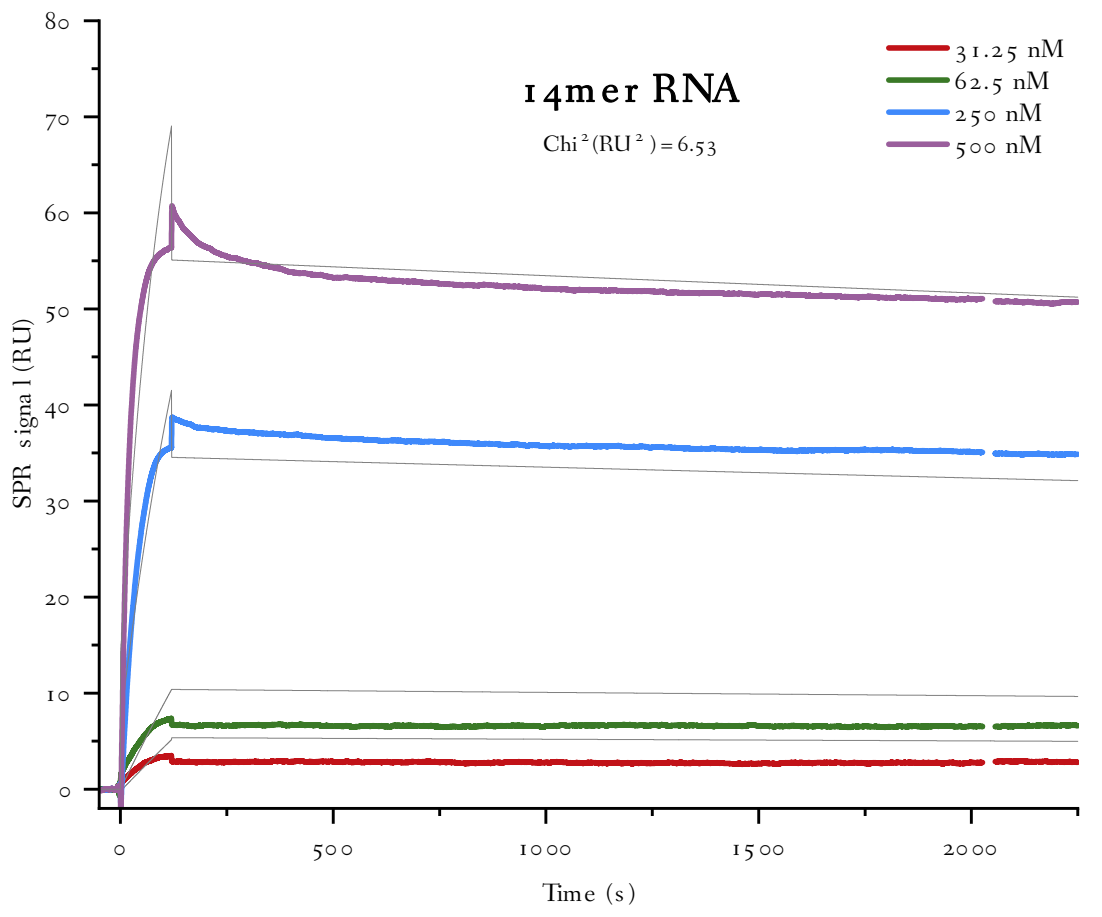
14mer RNA
Chi²(RU²) = 6.53
31.25 nM
62.5 nM
250 nM
500 nM
SPR signal (RU)
Time (s)

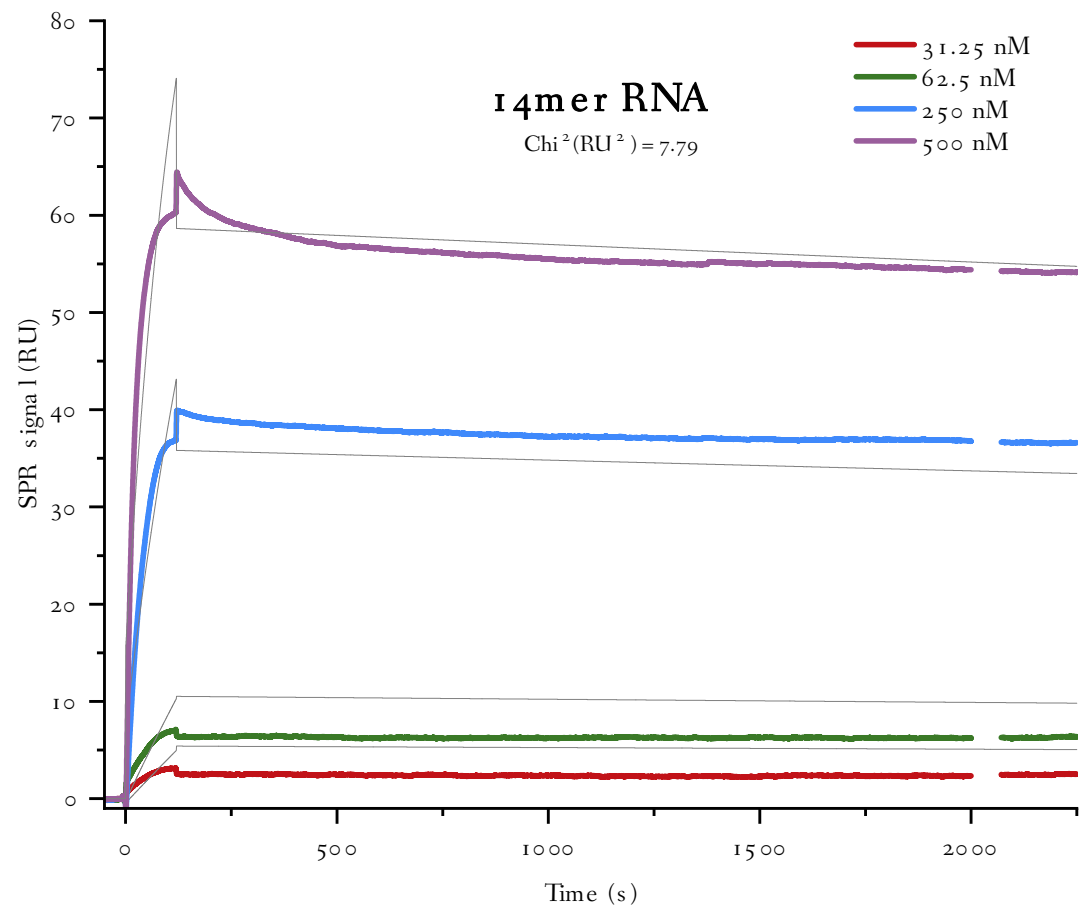
14mer RNA
Chi²(RU²) = 7.79
31.25 nM
62.5 nM
250 nM
500 nM
SPR signal (RU)
Time (s)

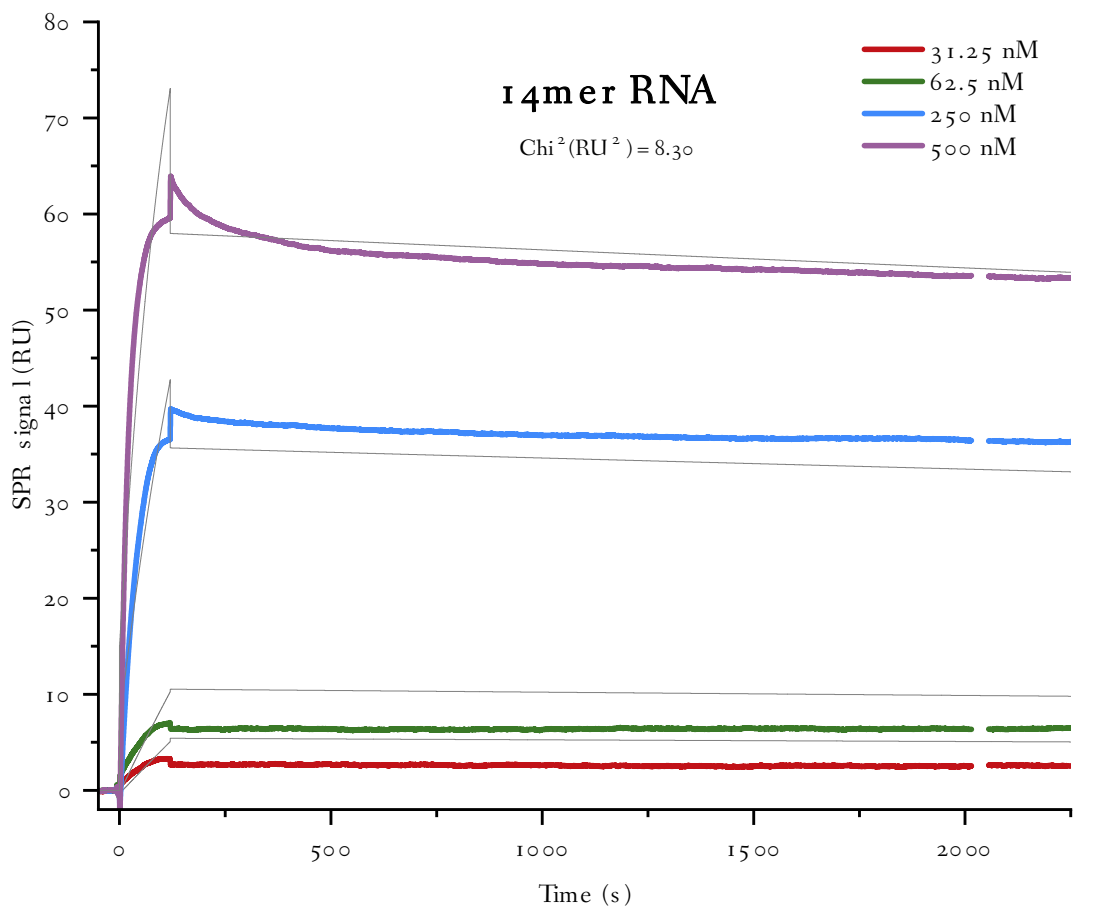
14mer RNA
Chi²(RU²) = 8.30
31.25 nM
62.5 nM
250 nM
500 nM
SPR signal (RU)
Time (s)

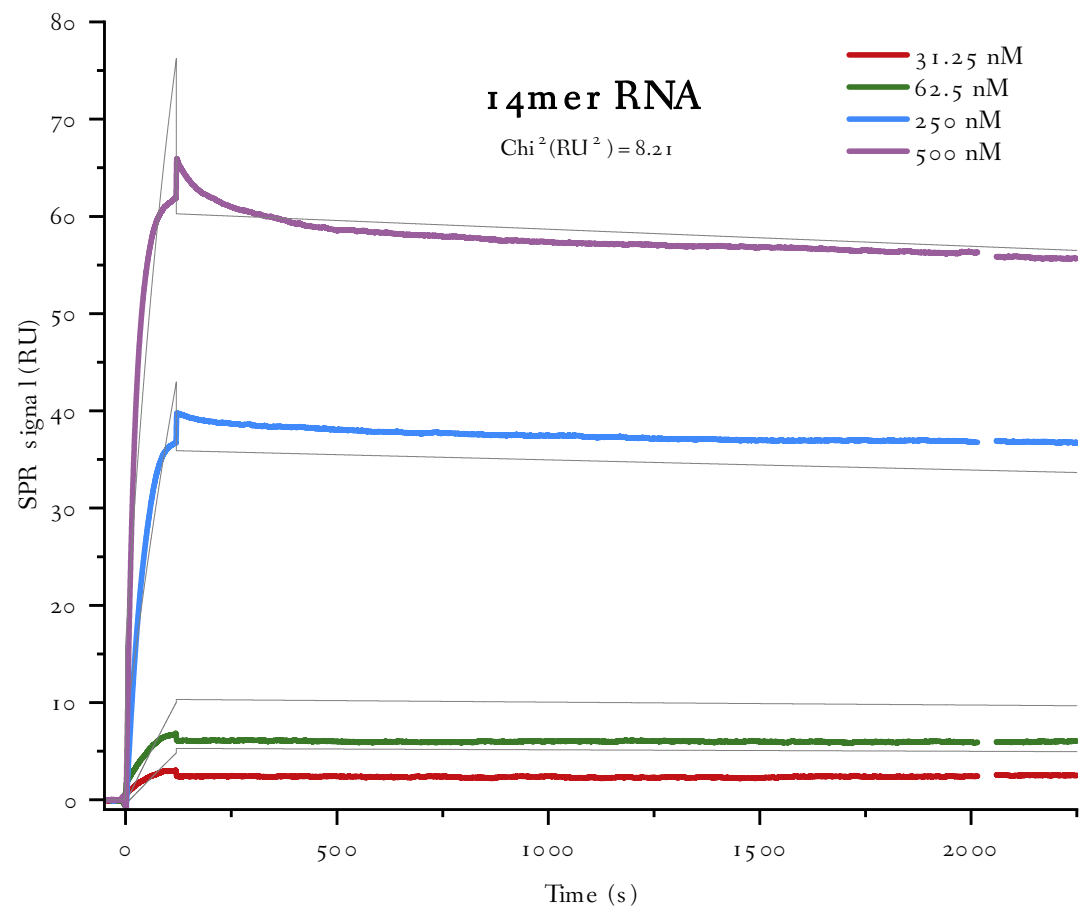
14mer RNA
Chi²(RU²) = 8.21
31.25 nM
62.5 nM
250 nM
500 nM
SPR signal (RU)
Time (s)

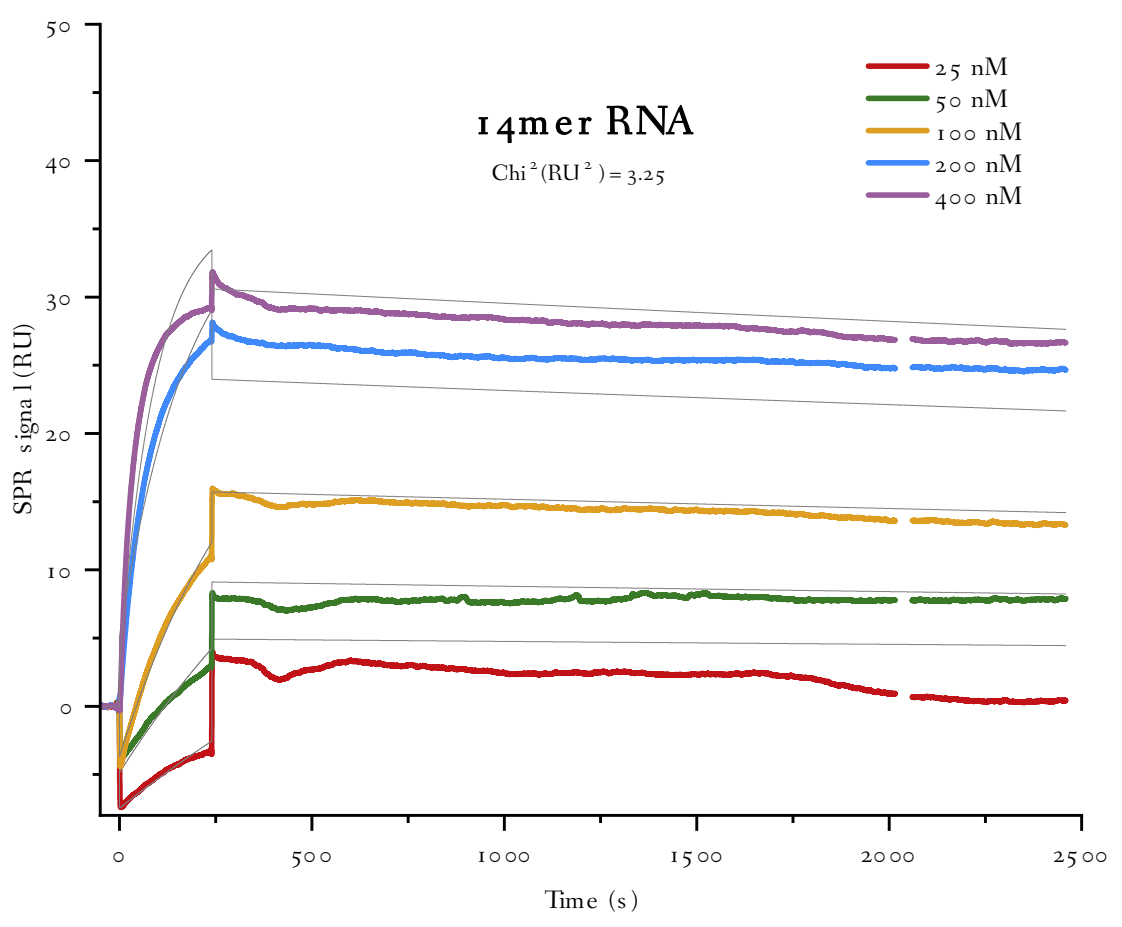
14mer RNA
Chi²(RU²) = 3.25
25 nM
50 nM
100 nM
200 nM
400 nM
SPR signal (RU)
Time (s)

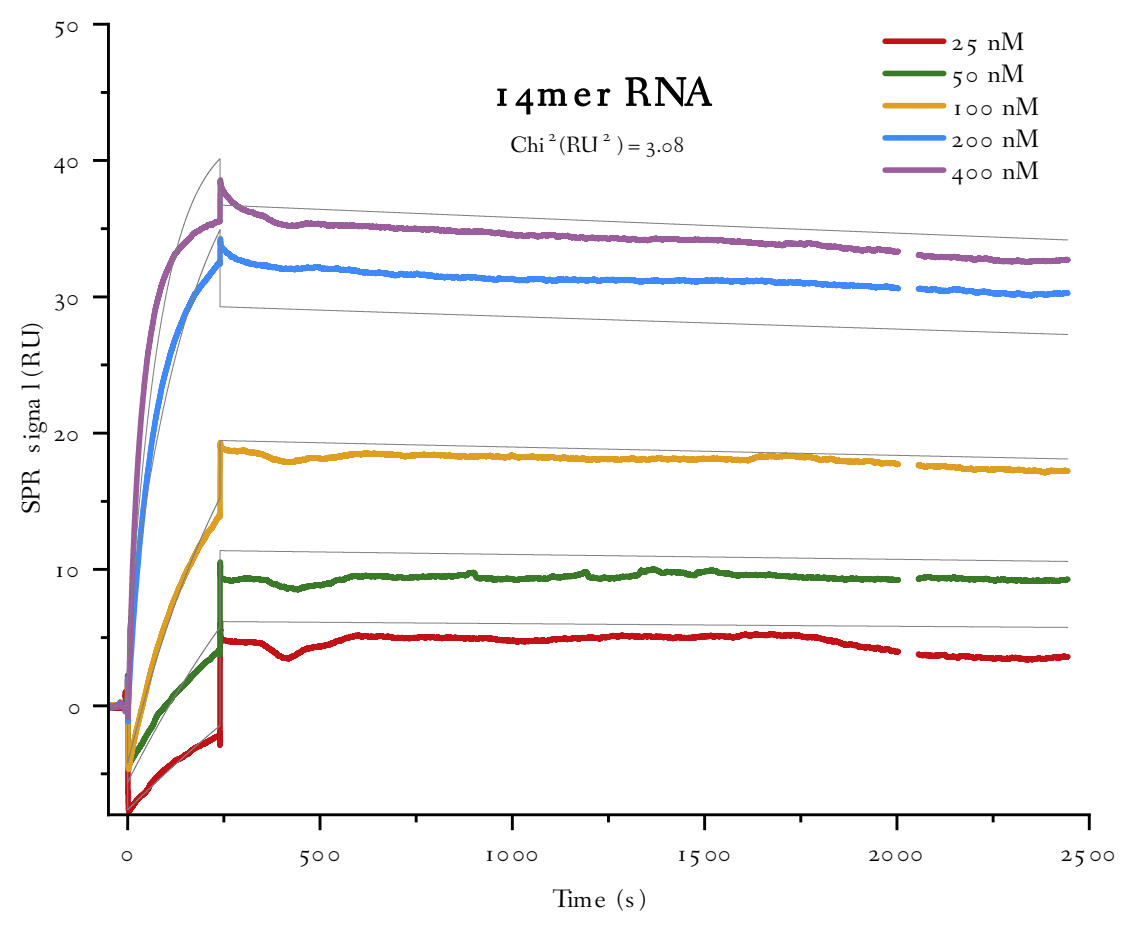
14mer RNA
Chi²(RU²) = 3.08
25 nM
50 nM
100 nM
200 nM
400 nM
SPR signal (RU)
Time (s)

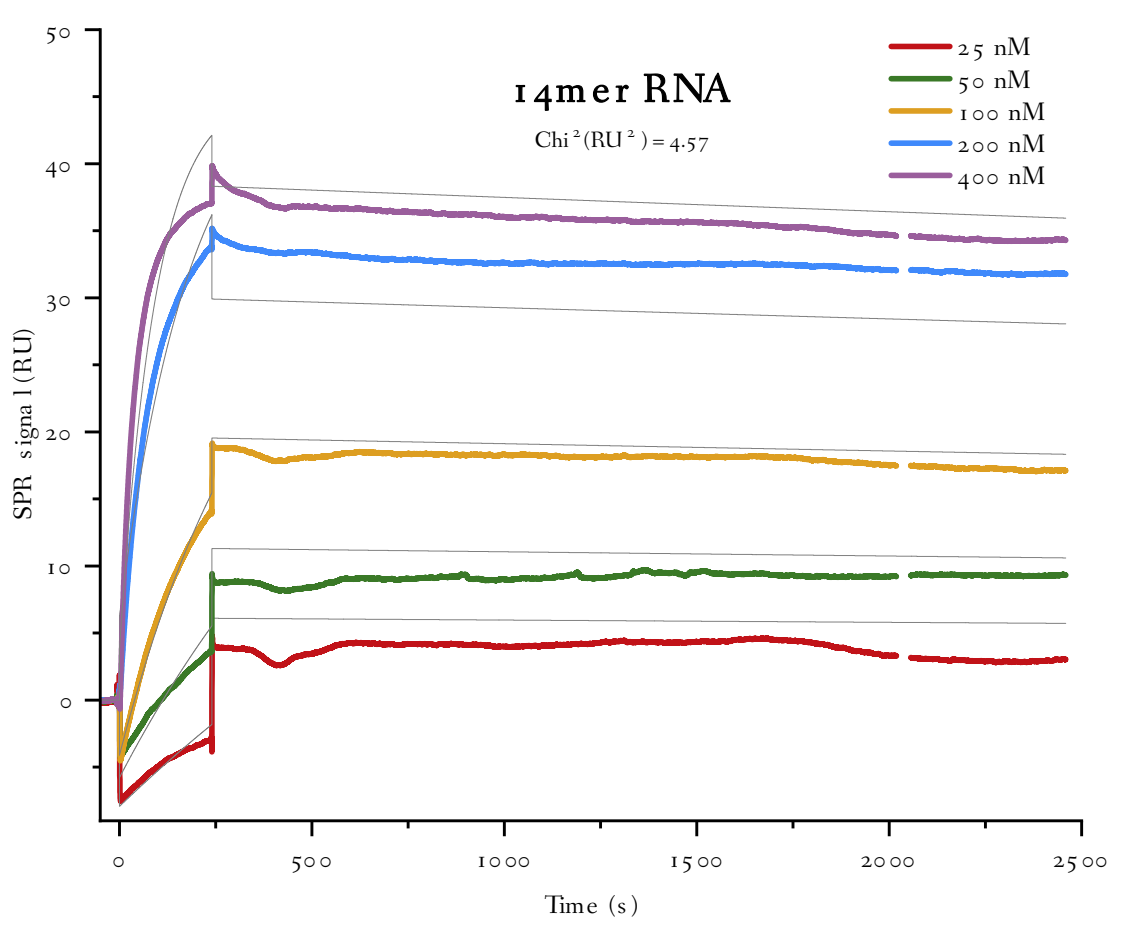
14mer RNA
Chi²(RU²) = 4.57
25 nM
50 nM
100 nM
200 nM
400 nM
SPR signal (RU)
Time (s)

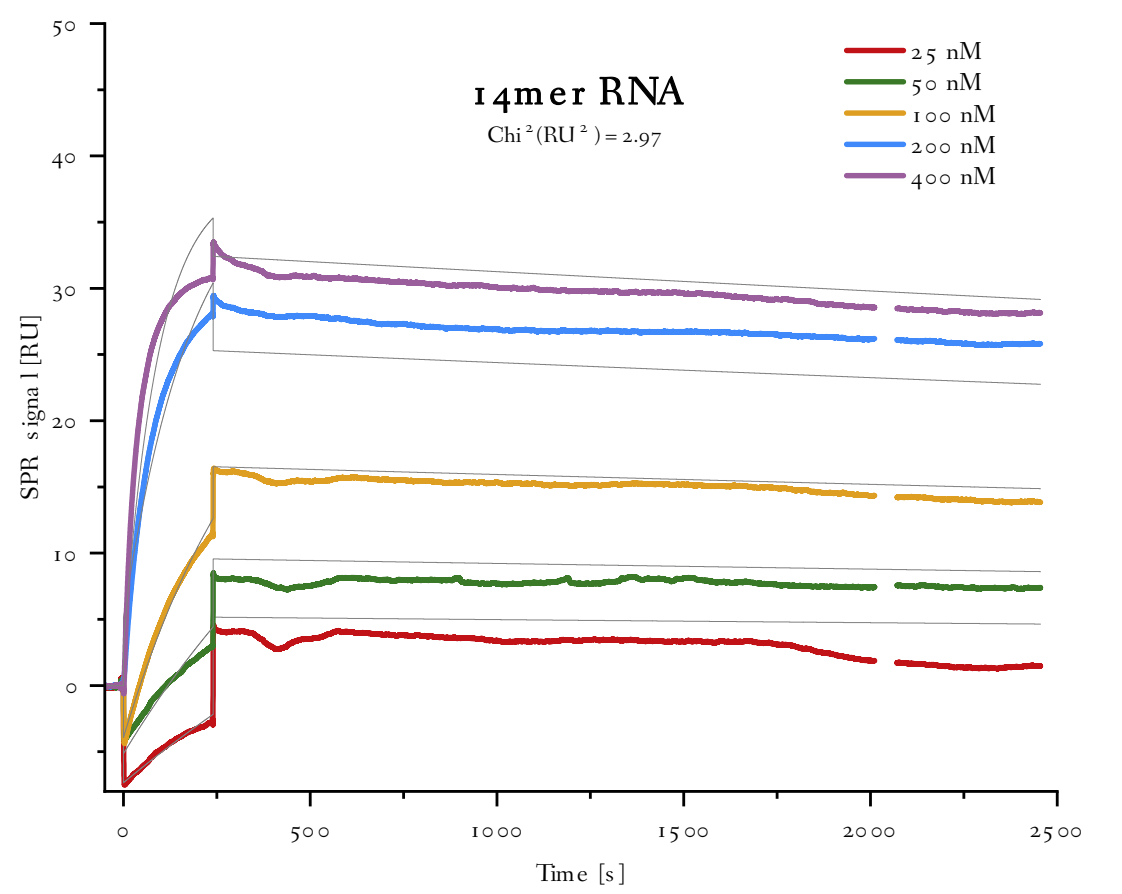
14mer RNA
Chi²(RU²) = 2.97
25 nM
50 nM
100 nM
200 nM
400 nM
SPR signal [RU]
Time [s]

5.4. 6mer PNA

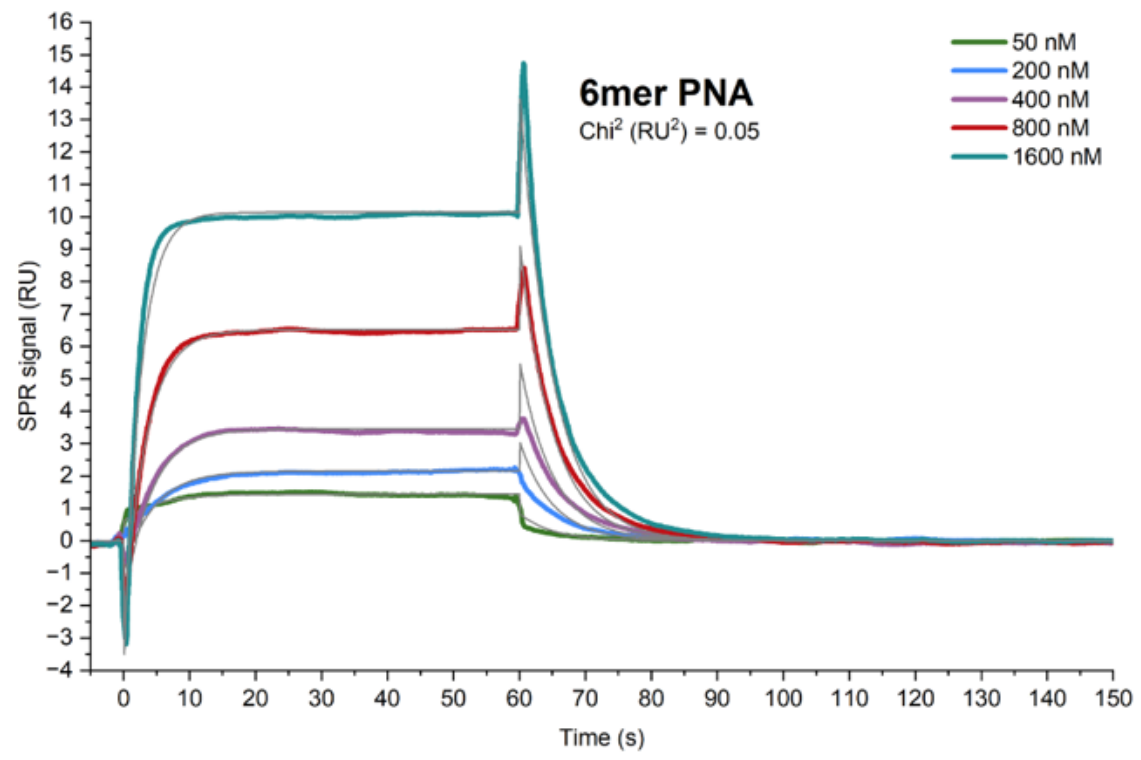
6mer PNA
Chi² (RU²) = 0.05
50 nM
200 nM
400 nM
800 nM
1600 nM
SPR signal (RU)
Time (s)

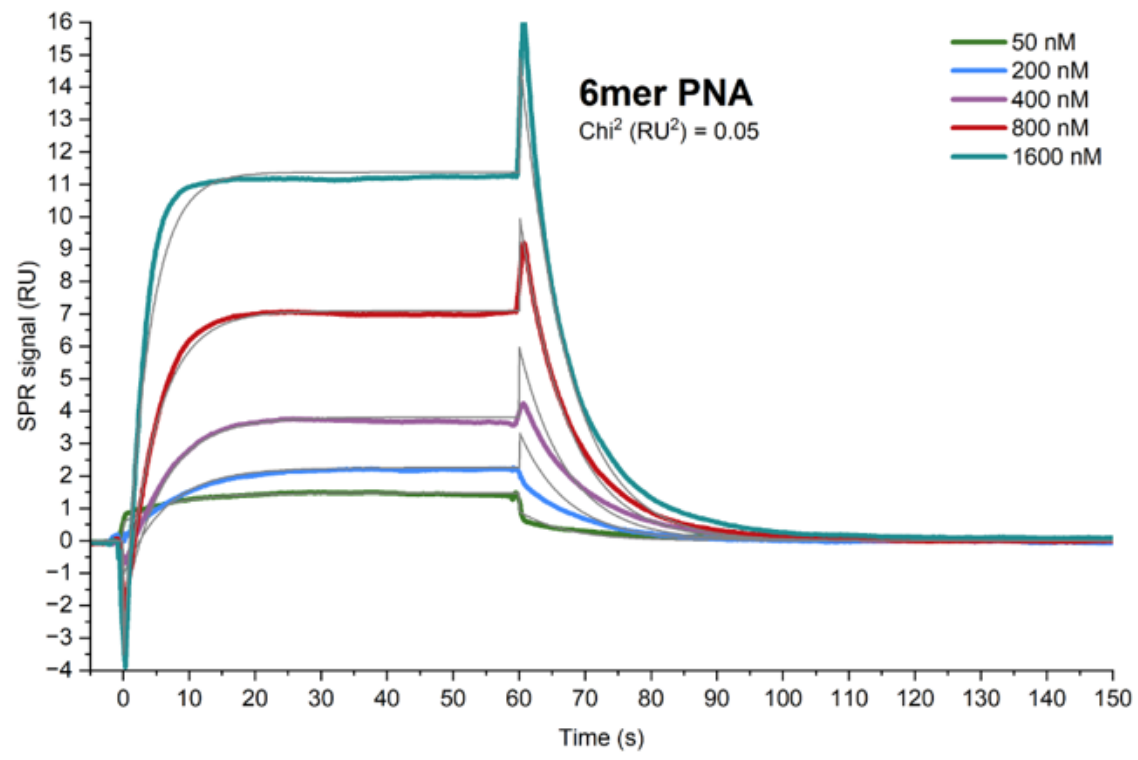
6mer PNA
Chi² (RU²) = 0.05
50 nM
200 nM
400 nM
800 nM
1600 nM
SPR signal (RU)
Time (s)

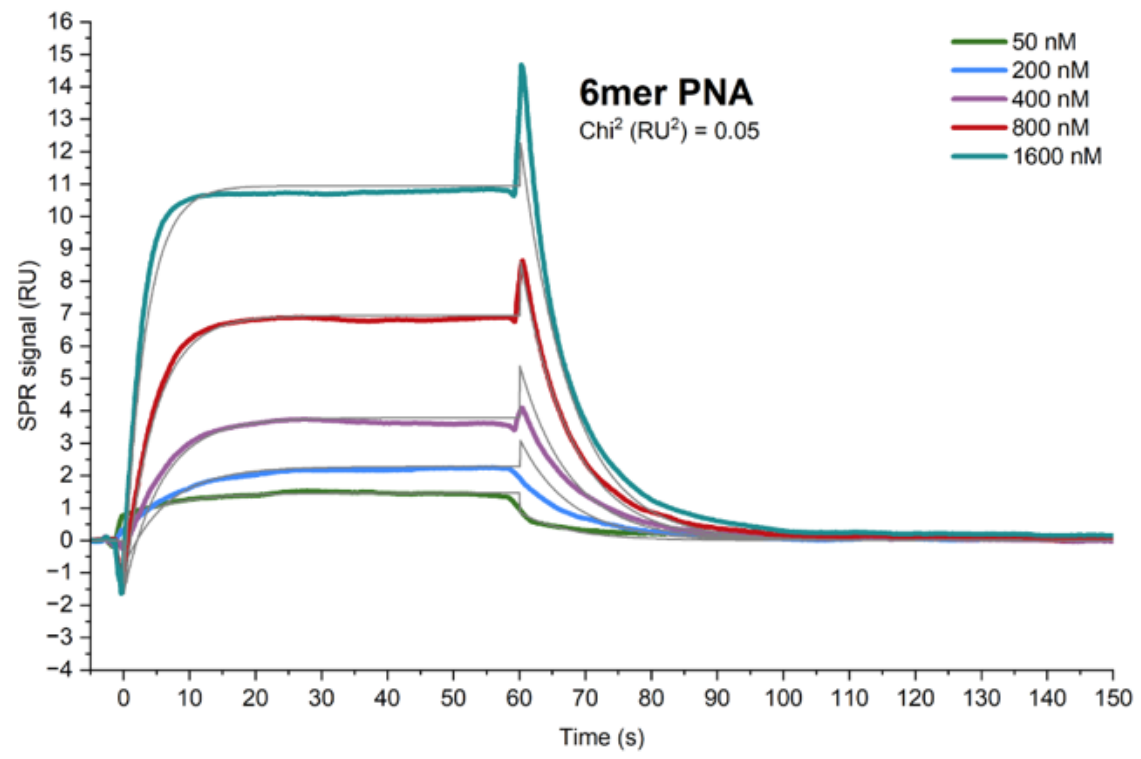
6mer PNA
Chi² (RU²) = 0.05
50 nM
200 nM
400 nM
800 nM
1600 nM
SPR signal (RU)
Time (s)

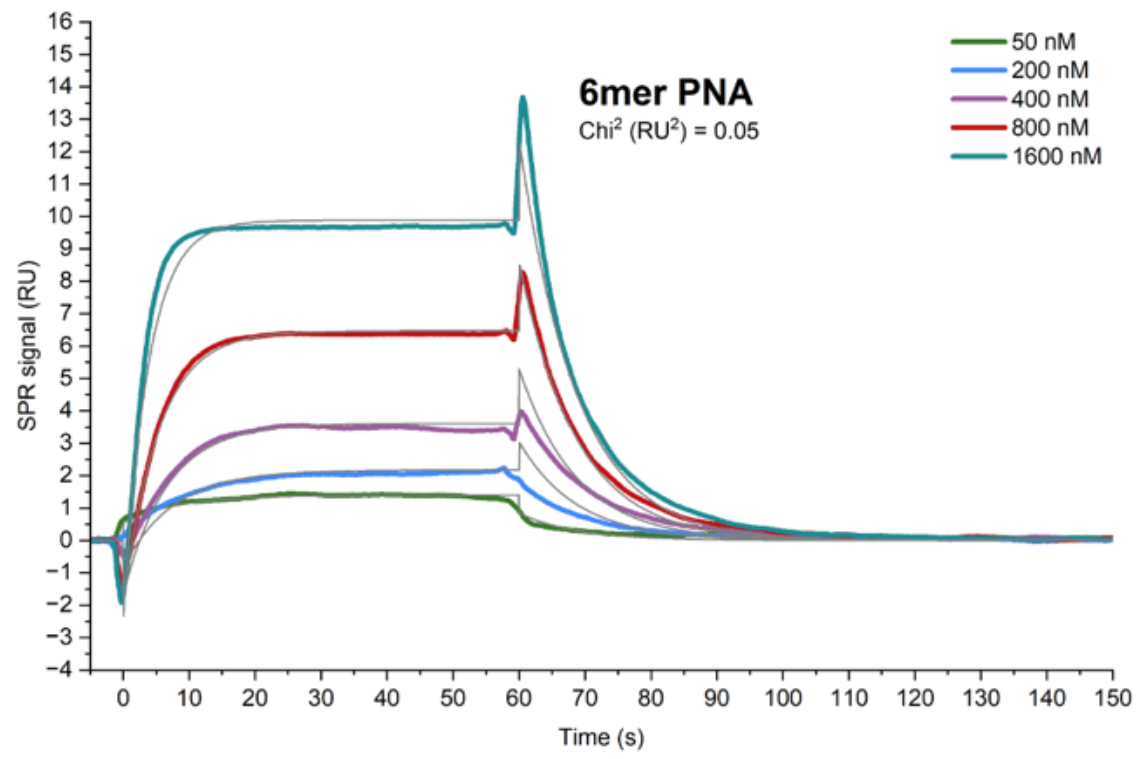
6mer PNA
Chi² (RU²) = 0.05
50 nM
200 nM
400 nM
800 nM
1600 nM
SPR signal (RU)
Time (s)

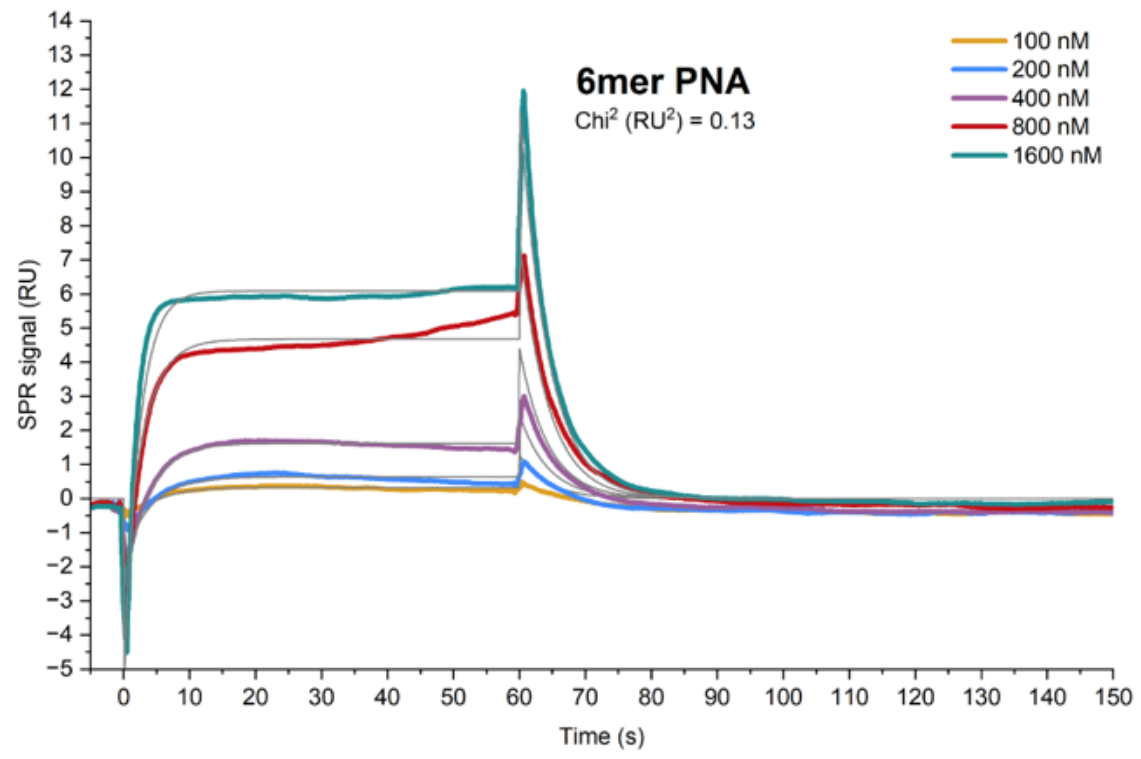
6mer PNA
Chi² (RU²) = 0.13
100 nM
200 nM
400 nM
800 nM
1600 nM
SPR signal (RU)
Time (s)

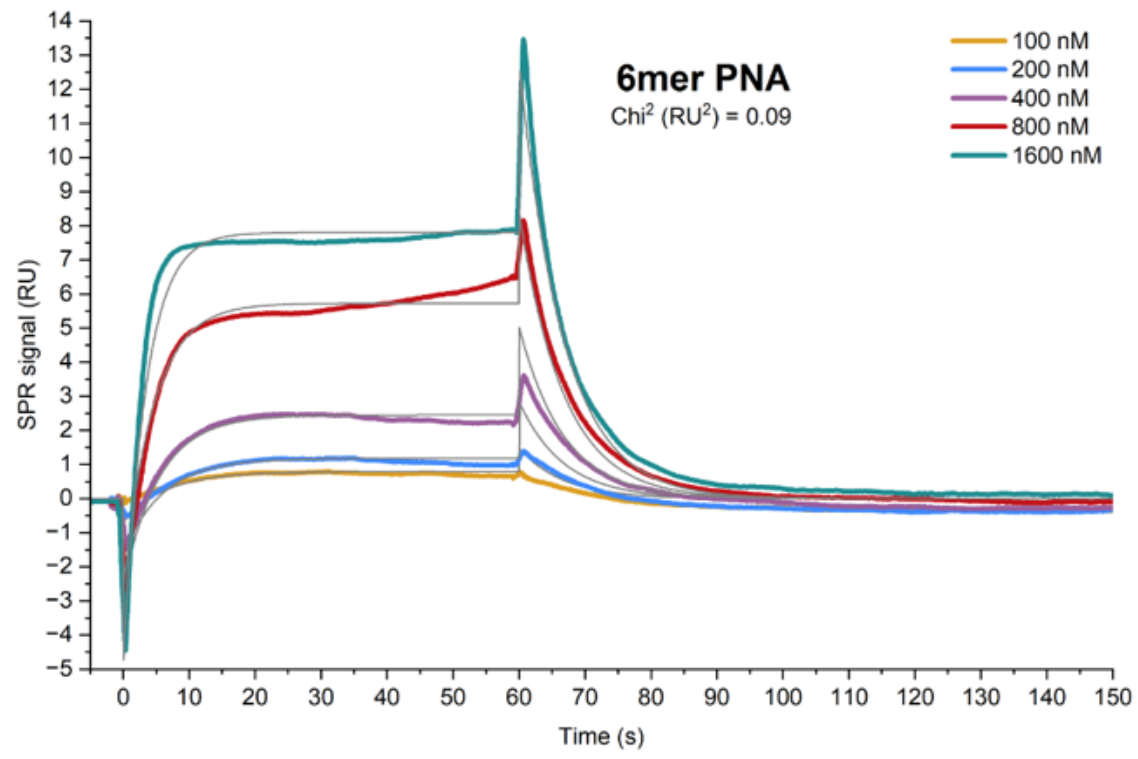
6mer PNA
Chi² (RU²) = 0.09
100 nM
200 nM
400 nM
800 nM
1600 nM
SPR signal (RU)
Time (s)

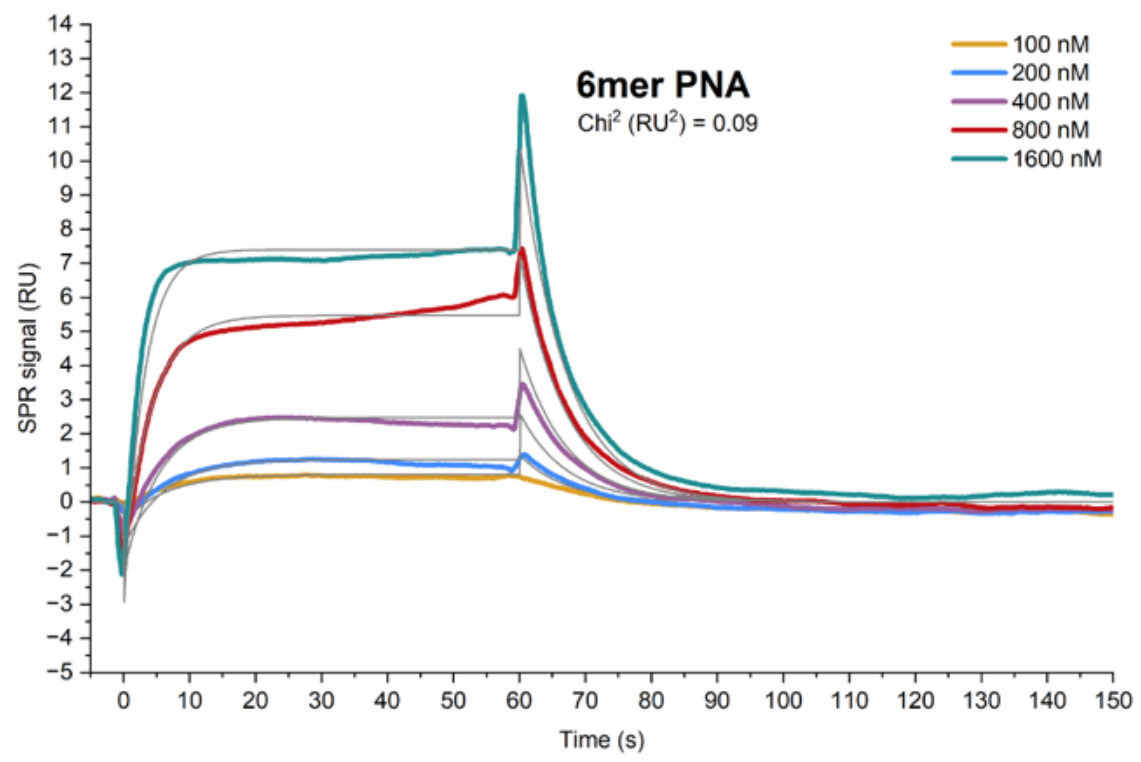
6mer PNA
Chi² (RU²) = 0.09
100 nM
200 nM
400 nM
800 nM
1600 nM
SPR signal (RU)
Time (s)

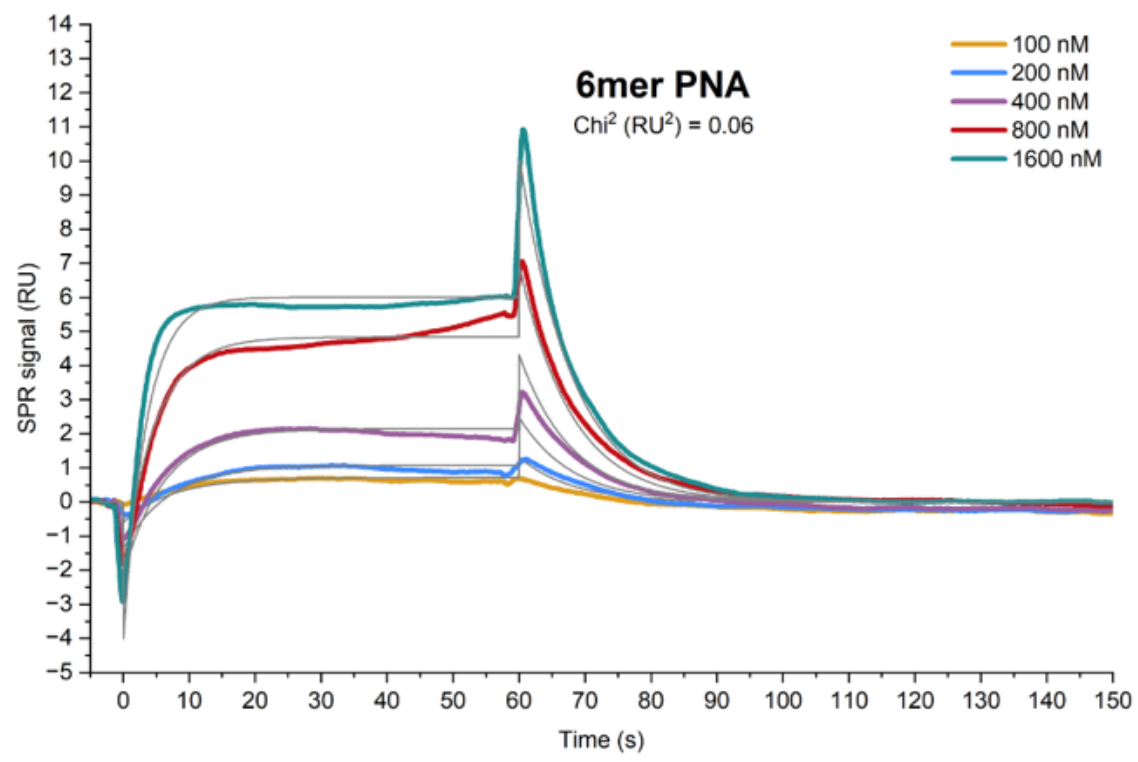
6mer PNA
Chi² (RU²) = 0.06
100 nM
200 nM
400 nM
800 nM
1600 nM
SPR signal (RU)
Time (s)

5.5. 10mer PNA

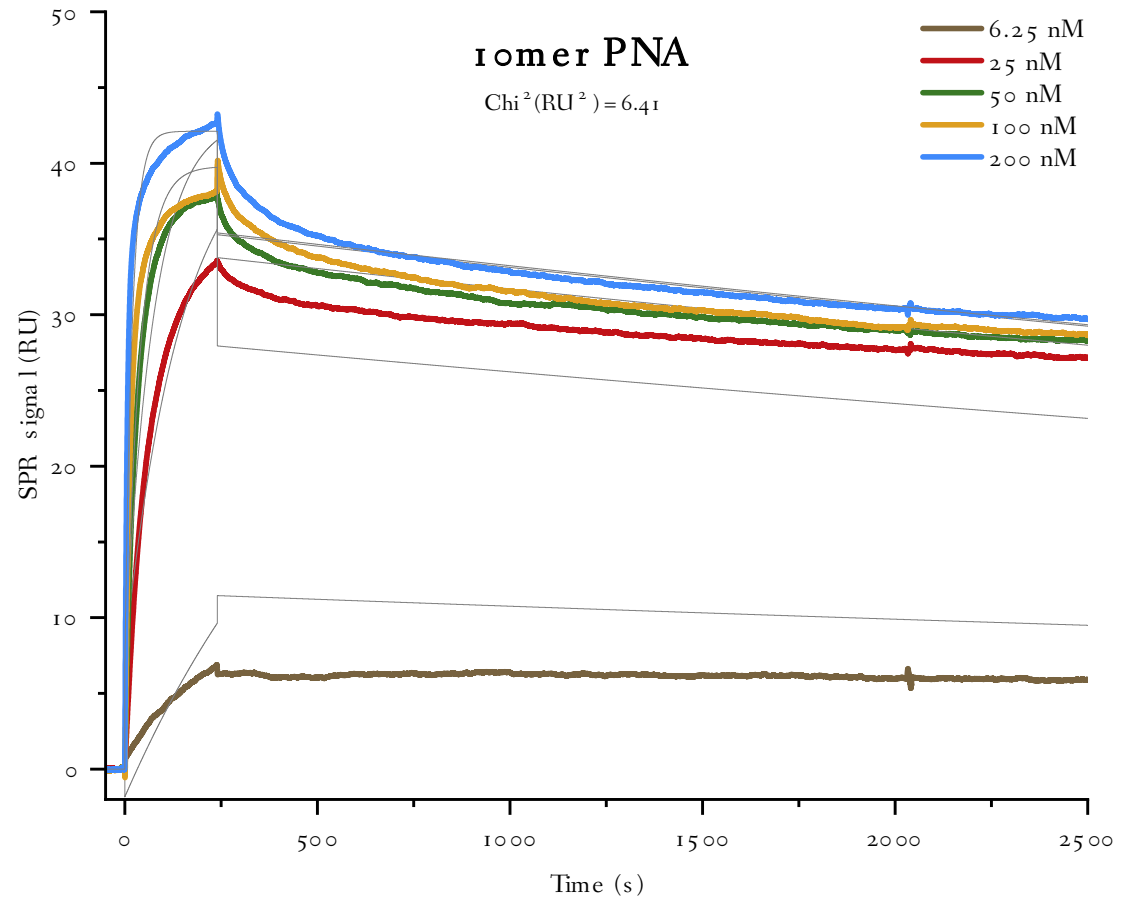

10mer PNA
$Chi^2 (RU^2) = 6.41$
6.25 nM
25 nM
50 nM
100 nM
200 nM
SPR signal (RU)
Time (s)

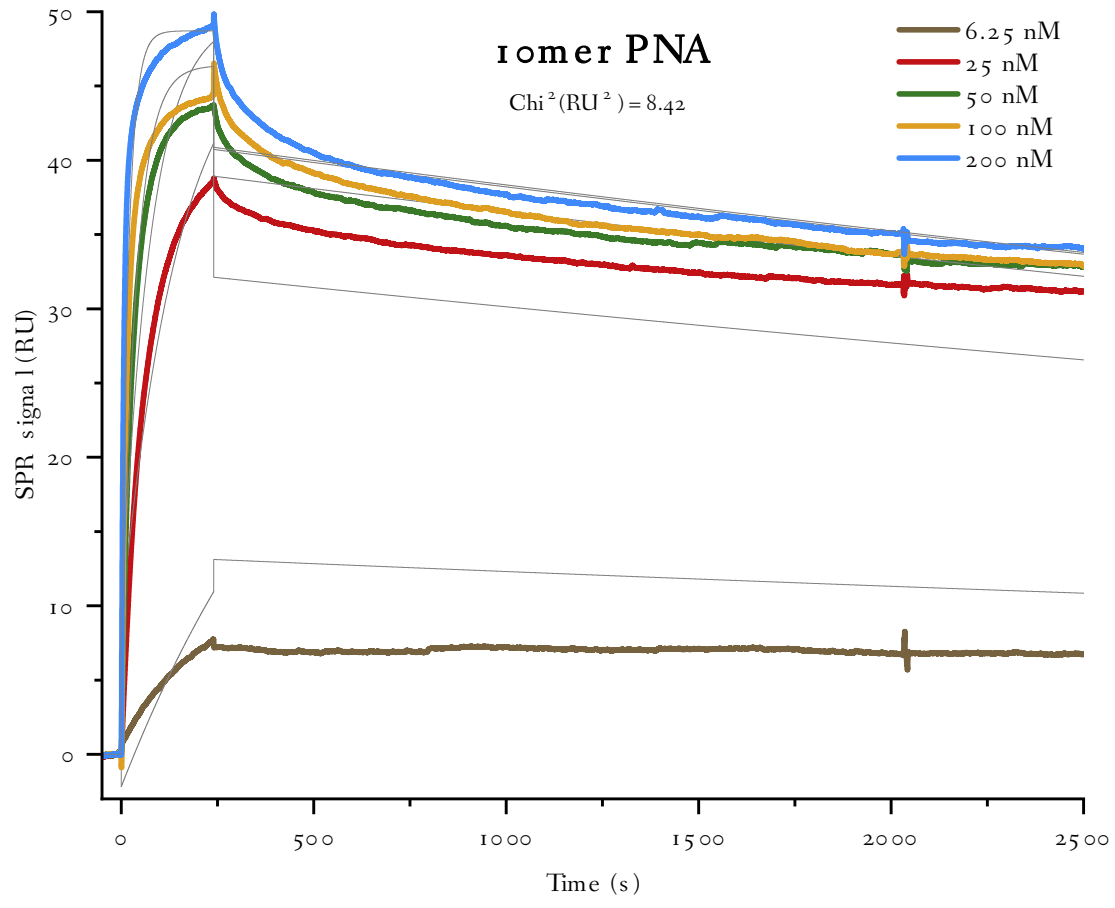

10mer PNA
$Chi^2 (RU^2) = 8.42$
6.25 nM
25 nM
50 nM
100 nM
200 nM
SPR signal (RU)
Time (s)

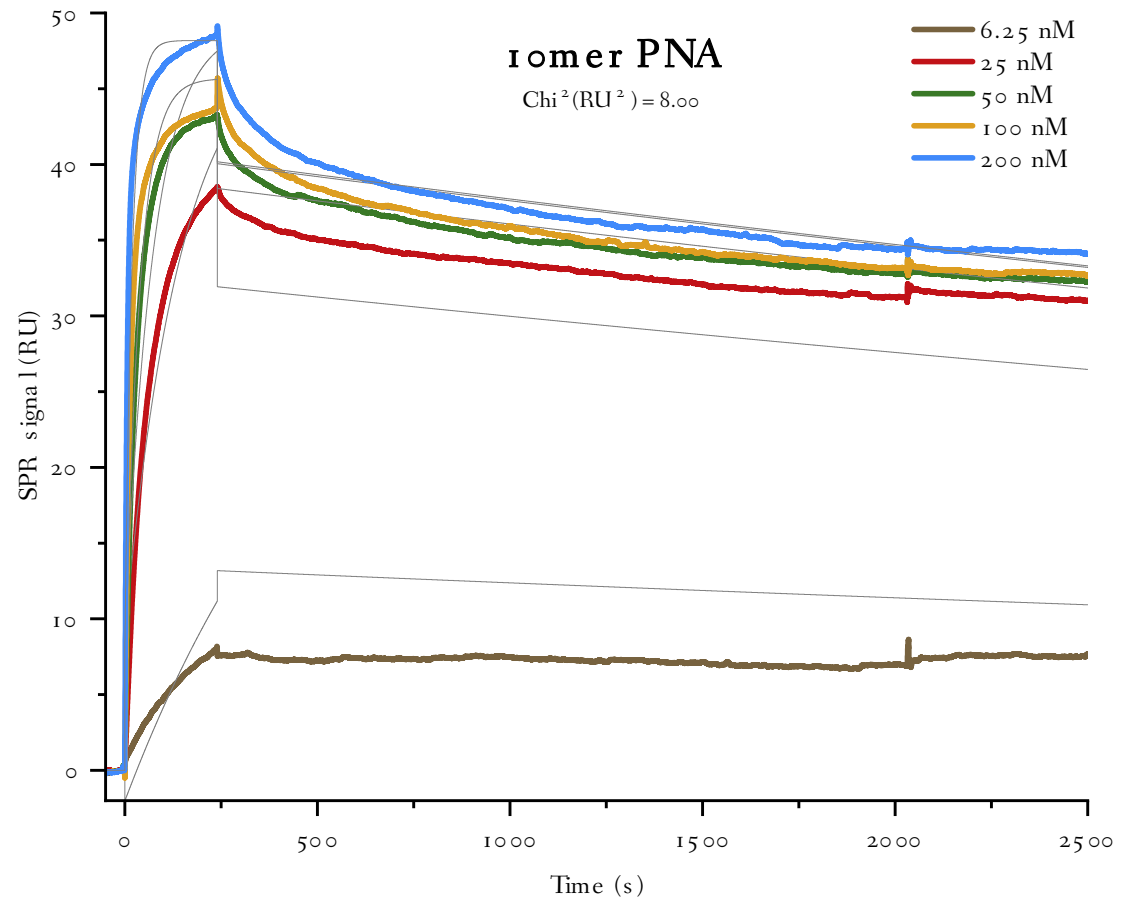

10mer PNA
$Chi^2 (RU^2) = 8.00$
6.25 nM
25 nM
50 nM
100 nM
200 nM
SPR signal (RU)
Time (s)

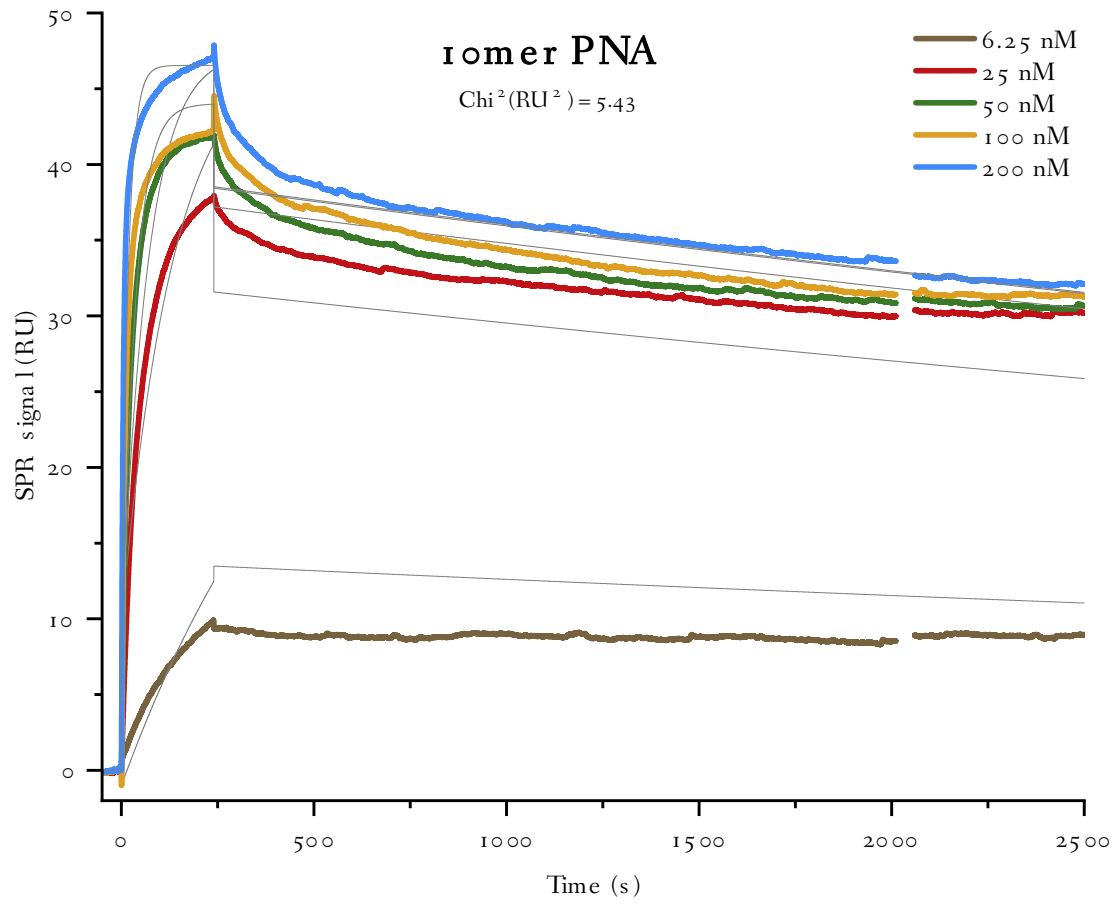

10mer PNA
$Chi^2 (RU^2) = 5.43$
6.25 nM
25 nM
50 nM
100 nM
200 nM
SPR signal (RU)
Time (s)

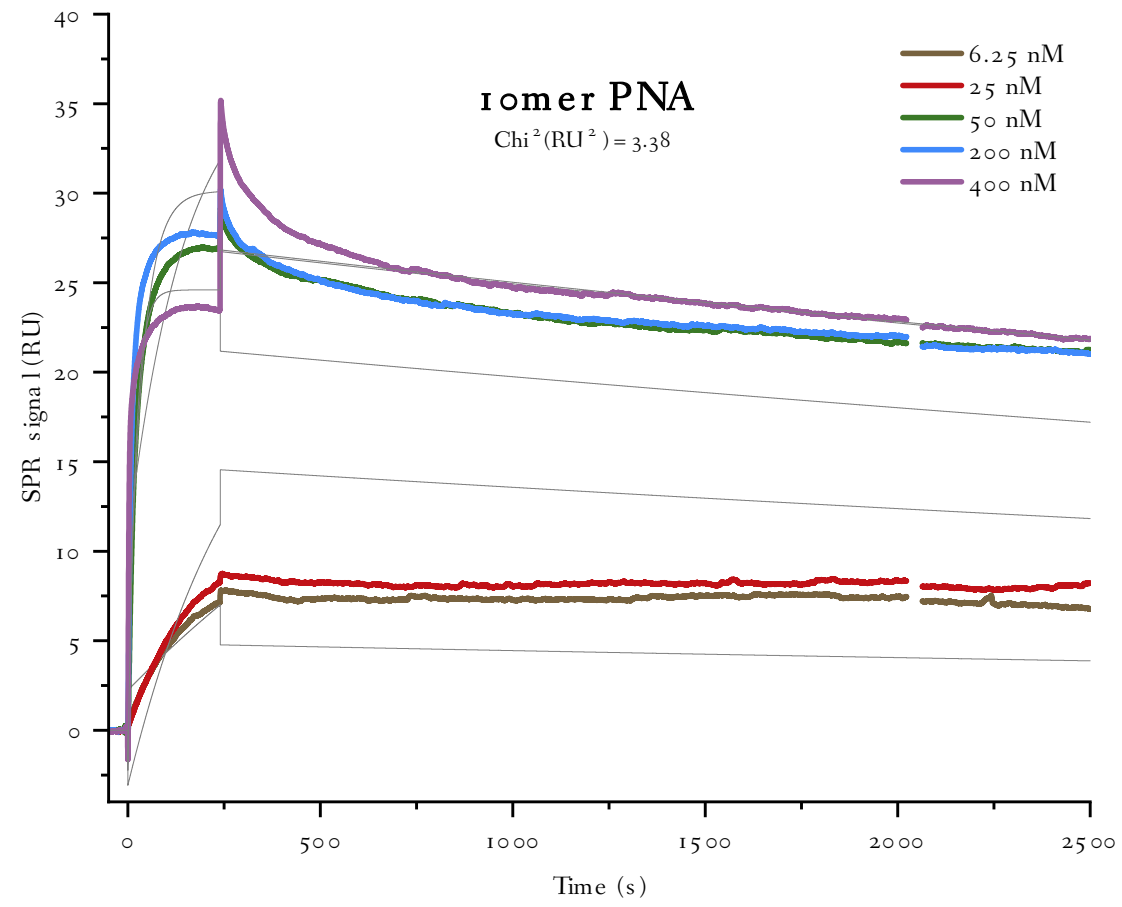

10mer PNA
$Chi^2 (RU^2) = 3.38$
6.25 nM
25 nM
50 nM
200 nM
400 nM
SPR signal (RU)
Time (s)

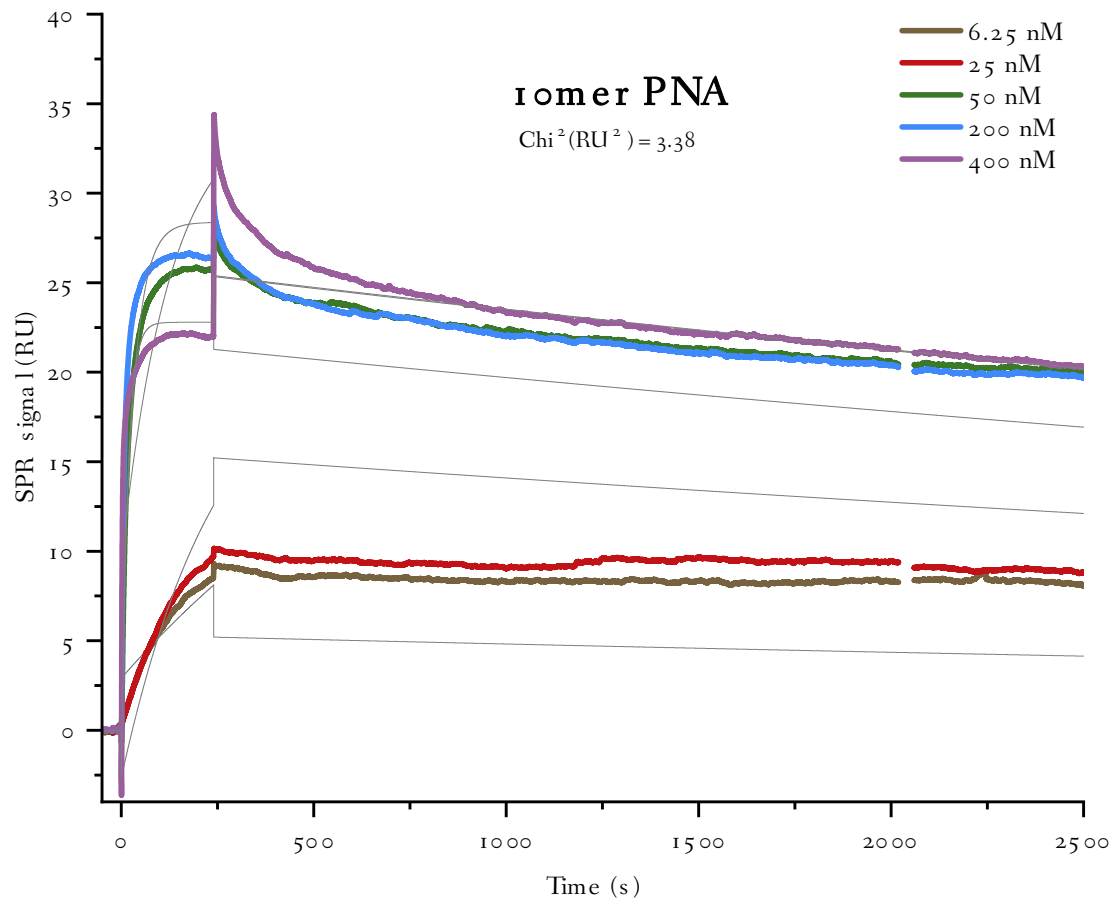

10mer PNA
$Chi^2 (RU^2) = 3.38$
6.25 nM
25 nM
50 nM
200 nM
400 nM
SPR signal (RU)
Time (s)

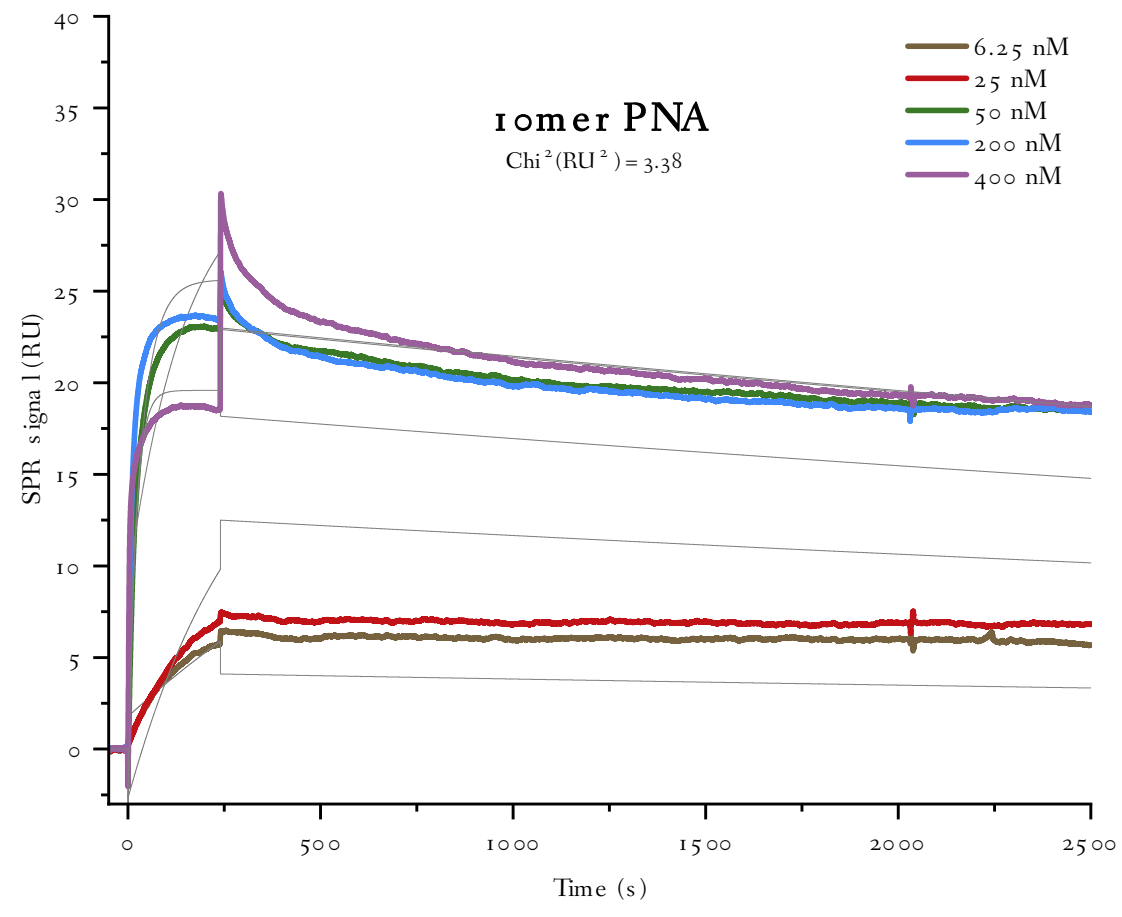

10mer PNA
$Chi^2 (RU^2) = 3.38$
6.25 nM
25 nM
50 nM
200 nM
400 nM
SPR signal (RU)
Time (s)

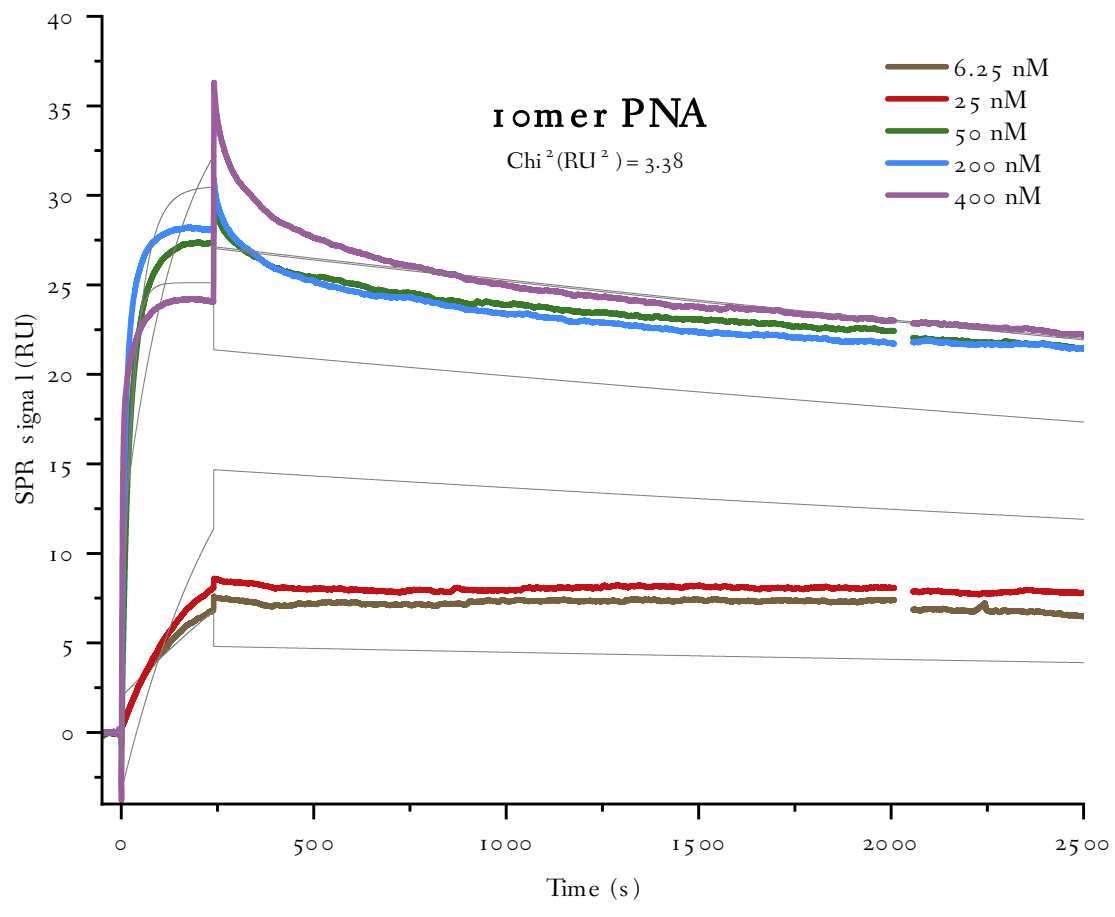

10mer PNA
$Chi^2 (RU^2) = 3.38$
6.25 nM
25 nM
50 nM
200 nM
400 nM
SPR signal (RU)
Time (s)

5.6. 12mer PNA

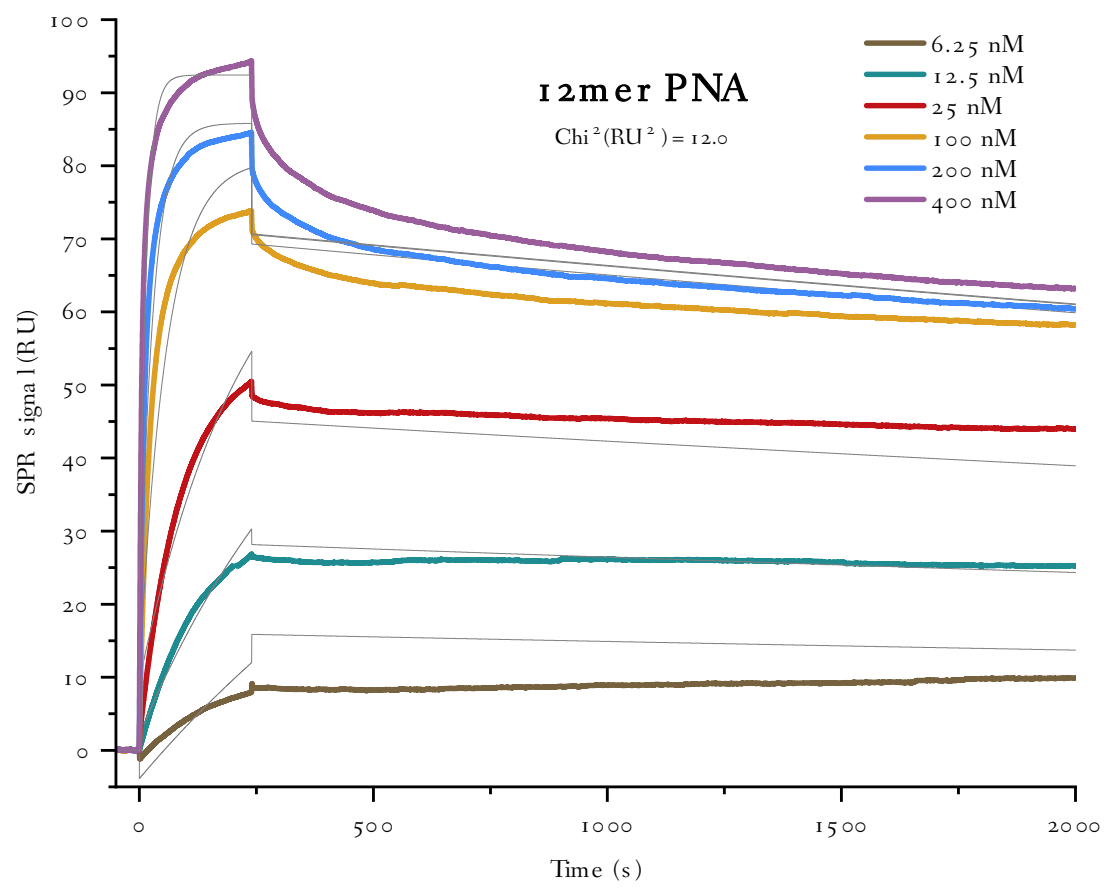
12mer PNA
Chi^2(RU^2) = 12.0
6.25 nM
12.5 nM
25 nM
100 nM
200 nM
400 nM
SPR signal (RU)
Time (s)

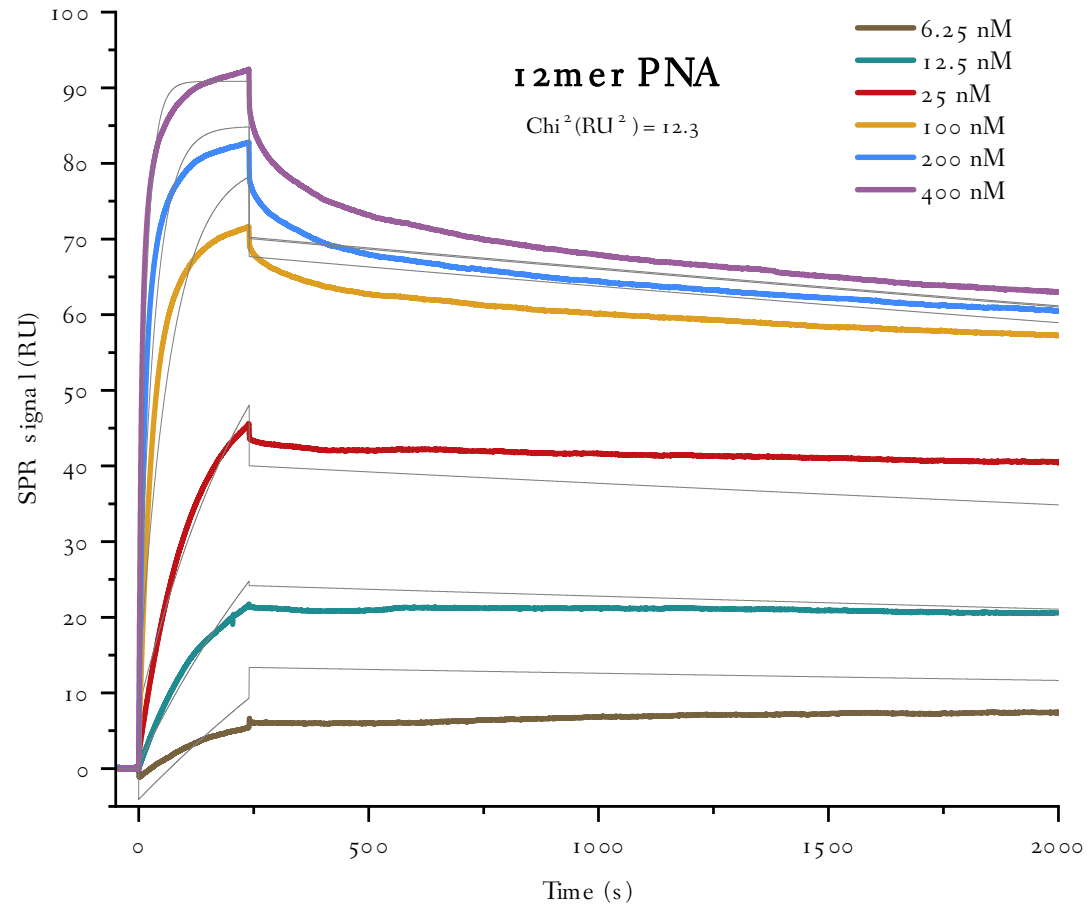
12mer PNA
Chi^2(RU^2) = 12.3
6.25 nM
12.5 nM
25 nM
100 nM
200 nM
400 nM
SPR signal (RU)
Time (s)

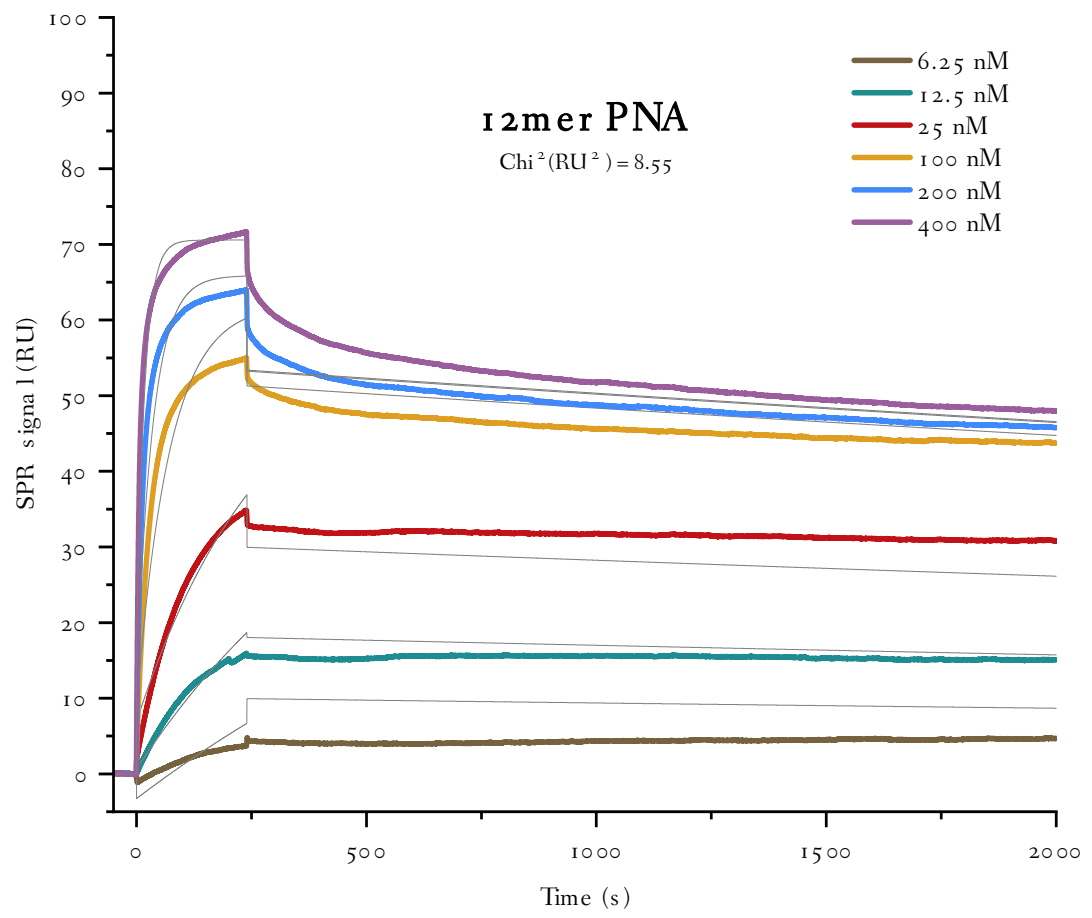
12mer PNA
Chi^2(RU^2) = 8.55
6.25 nM
12.5 nM
25 nM
100 nM
200 nM
400 nM
SPR signal (RU)
Time (s)

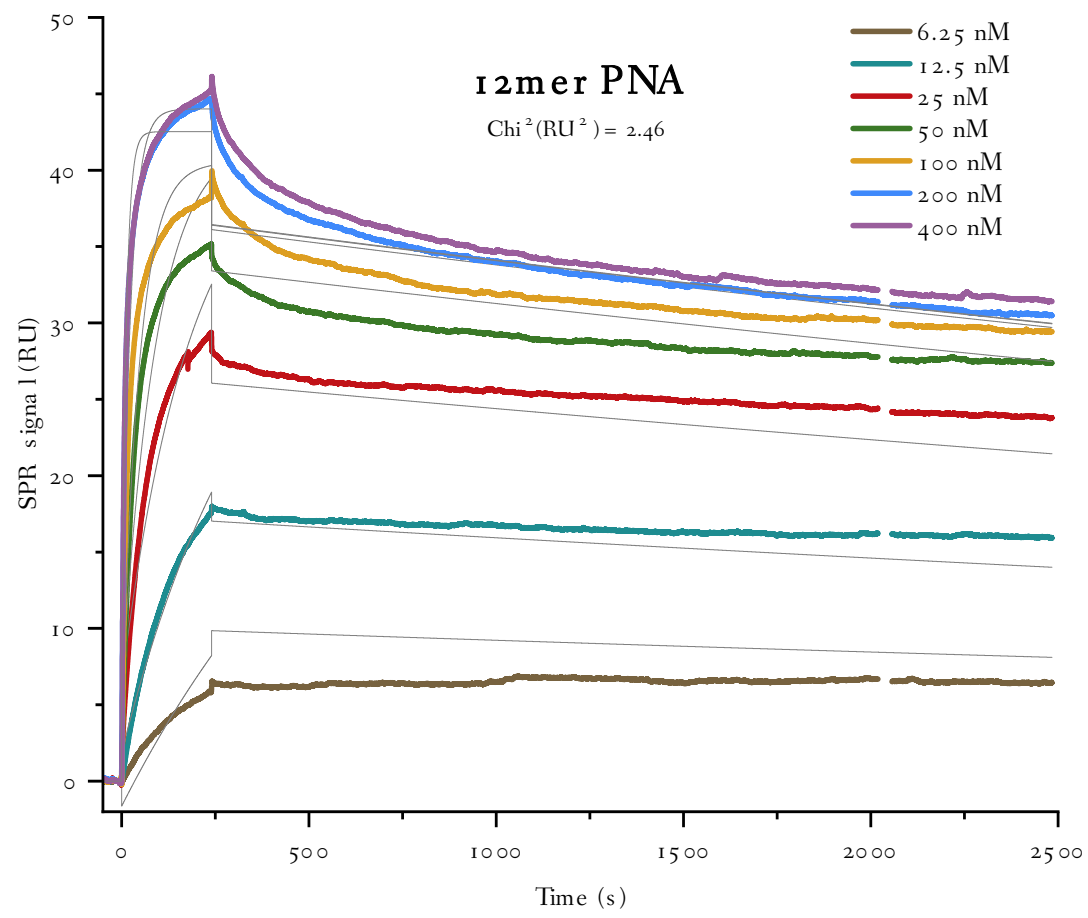
12mer PNA
Chi^2(RU^2) = 2.46
6.25 nM
12.5 nM
25 nM
50 nM
100 nM
200 nM
400 nM
SPR signal (RU)
Time (s)

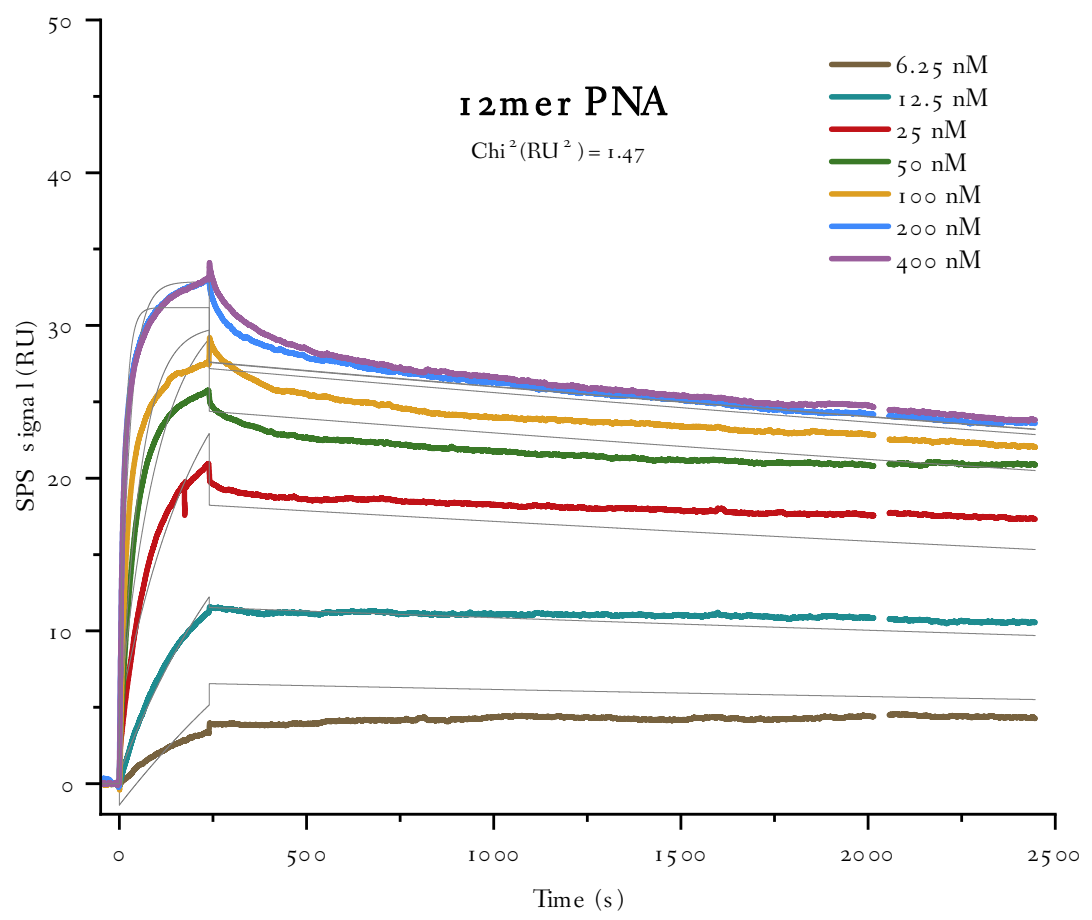
12mer PNA
Chi^2(RU^2) = 1.47
6.25 nM
12.5 nM
25 nM
50 nM
100 nM
200 nM
400 nM
SPS signal (RU)
Time (s)

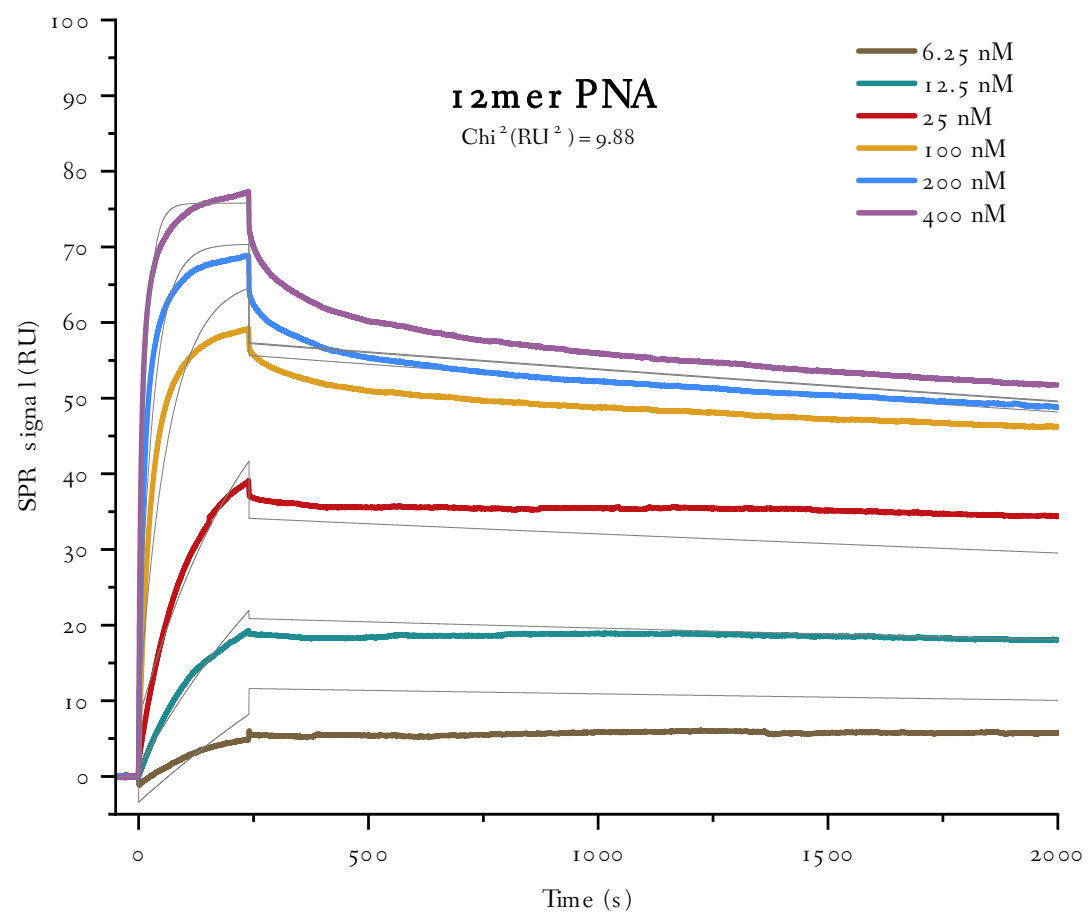
12mer PNA
Chi^2(RU^2) = 9.88
6.25 nM
12.5 nM
25 nM
100 nM
200 nM
400 nM
SPR signal (RU)
Time (s)

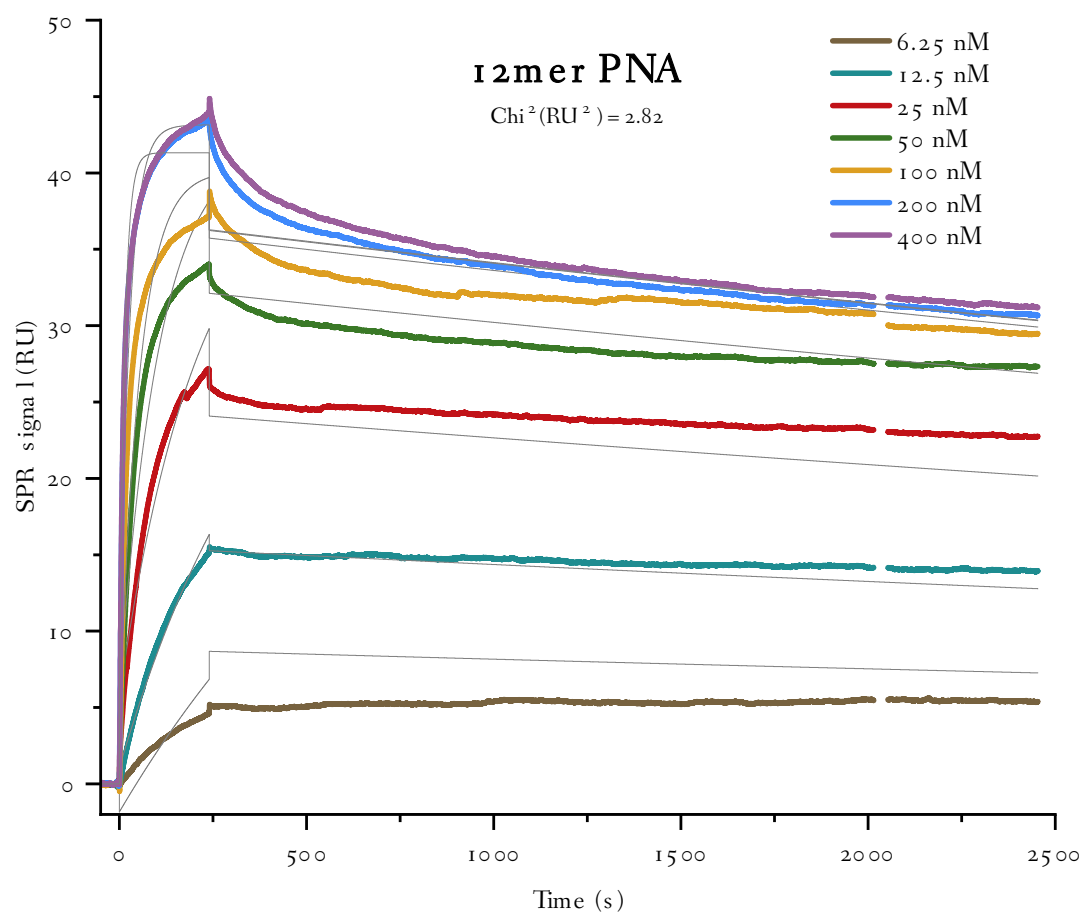
12mer PNA
Chi^2(RU^2) = 2.82
6.25 nM
12.5 nM
25 nM
50 nM
100 nM
200 nM
400 nM
SPR signal (RU)
Time (s)

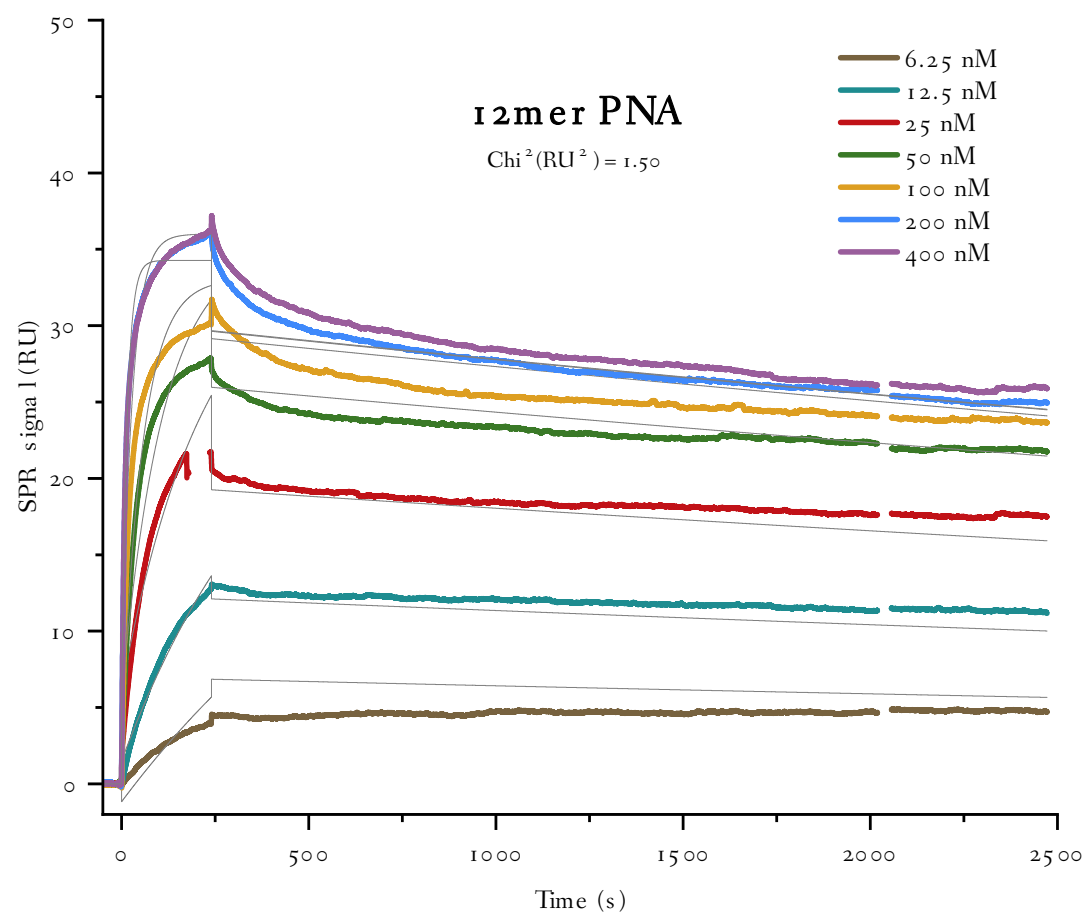
12mer PNA
Chi^2(RU^2) = 1.50
6.25 nM
12.5 nM
25 nM
50 nM
100 nM
200 nM
400 nM
SPR signal (RU)
Time (s)

5.7. 14mer PNA

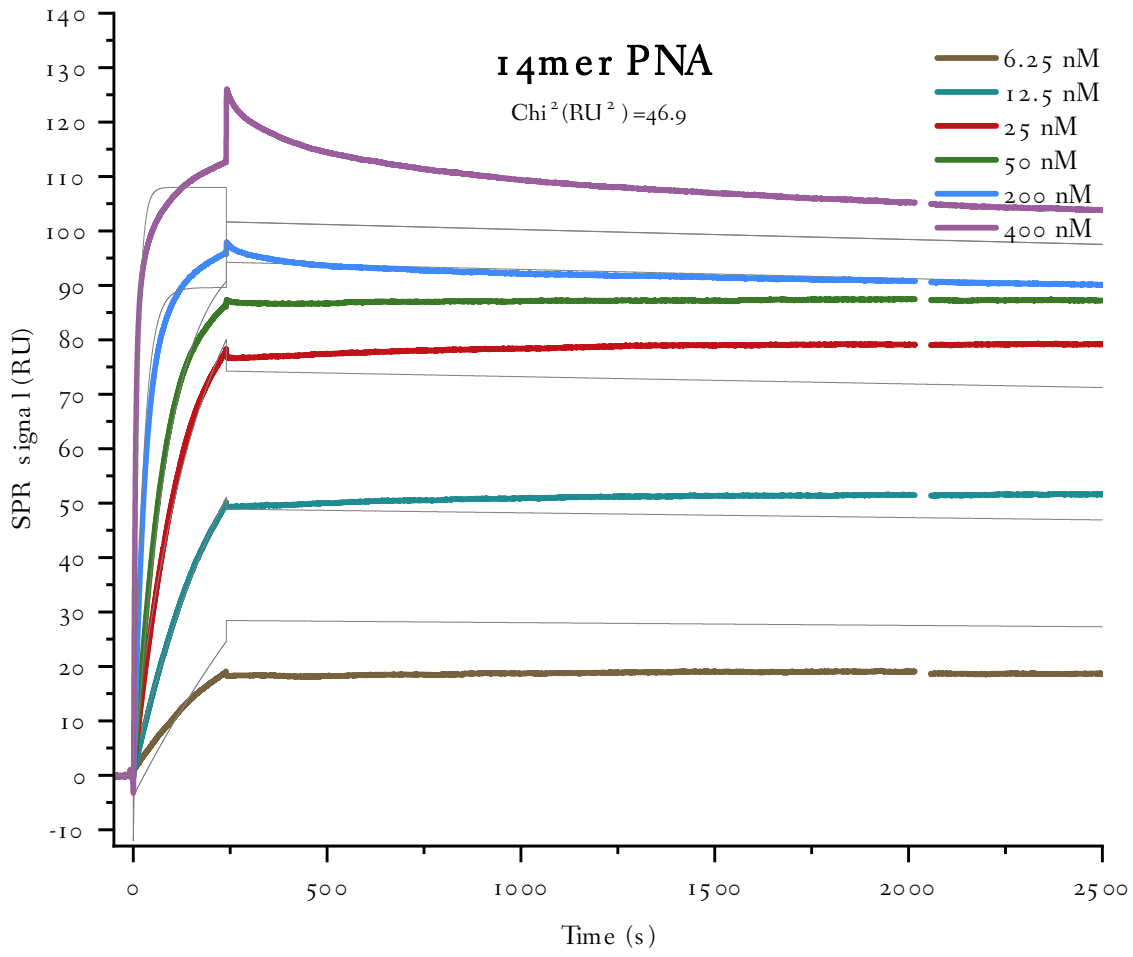

14mer PNA
$Chi^2 (RU^2) = 46.9$
6.25 nM
12.5 nM
25 nM
50 nM
200 nM
400 nM
SPR signal (RU)
Time (s)
140
130
120
110
100
90
80
70
60
50
40
30
20
10
0
-10
0
500
1000
1500
2000
2500

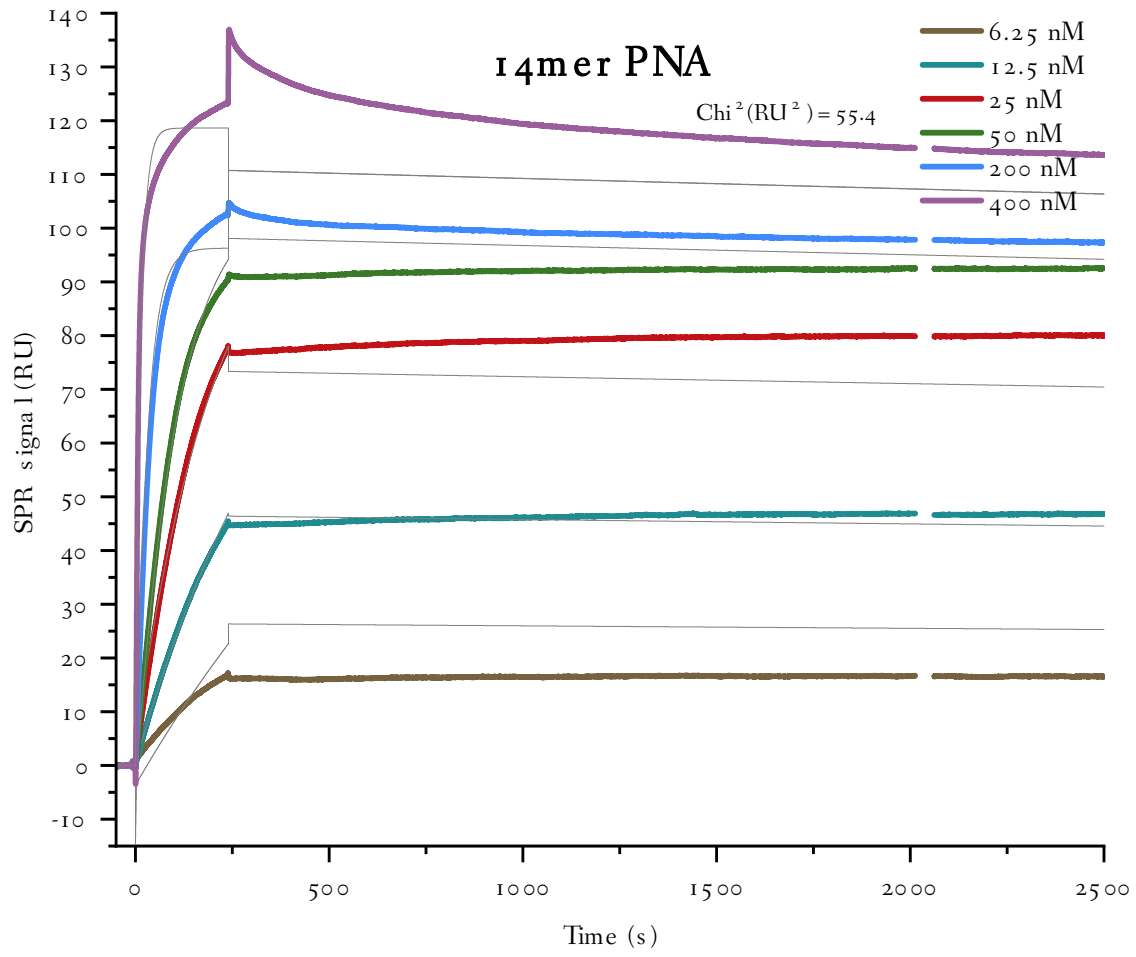

14mer PNA
$Chi^2 (RU^2) = 55.4$
6.25 nM
12.5 nM
25 nM
50 nM
200 nM
400 nM
SPR signal (RU)
Time (s)
140
130
120
110
100
90
80
70
60
50
40
30
20
10
0
-10
0
500
1000
1500
2000
2500

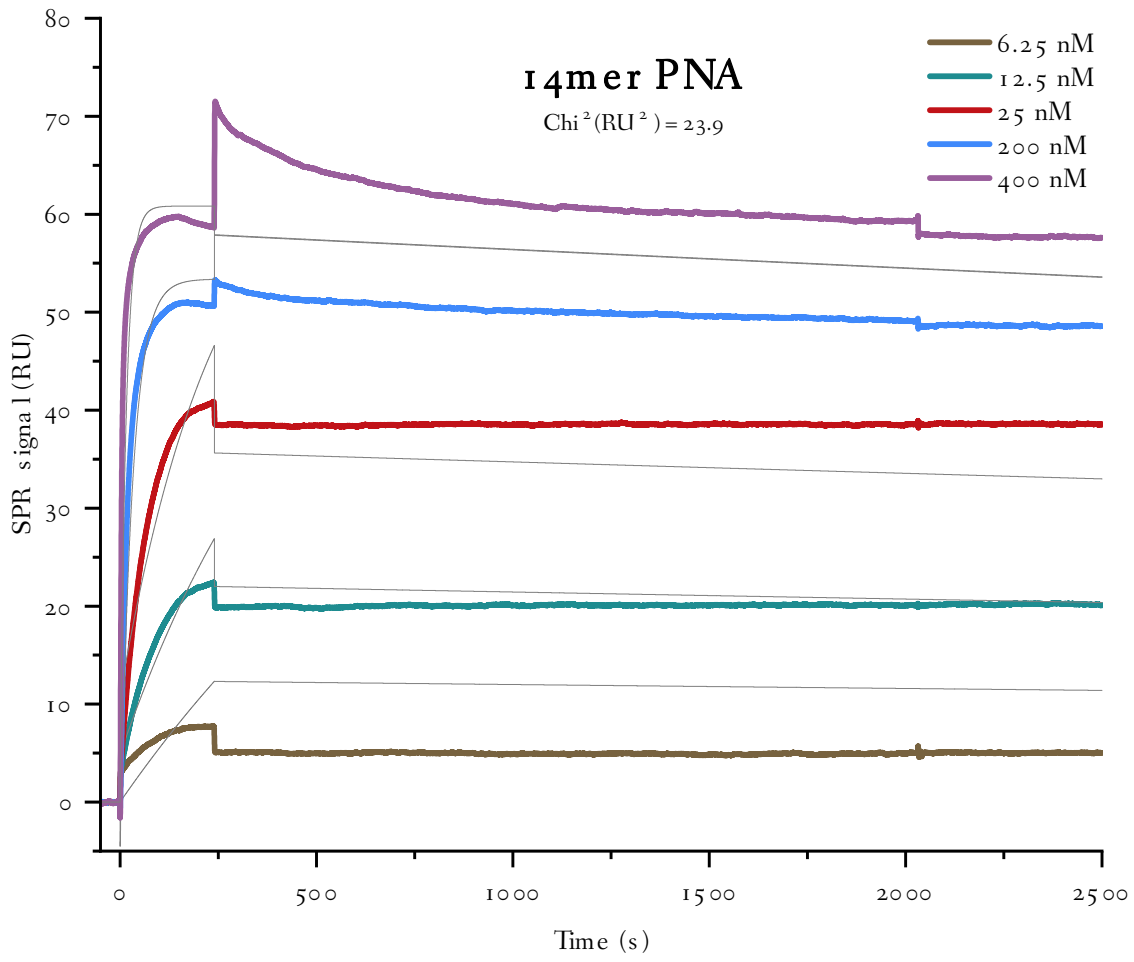

14mer PNA
$Chi^2 (RU^2) = 23.9$
6.25 nM
12.5 nM
25 nM
200 nM
400 nM
SPR signal (RU)
Time (s)
80
70
60
50
40
30
20
10
0
0
500
1000
1500
2000
2500

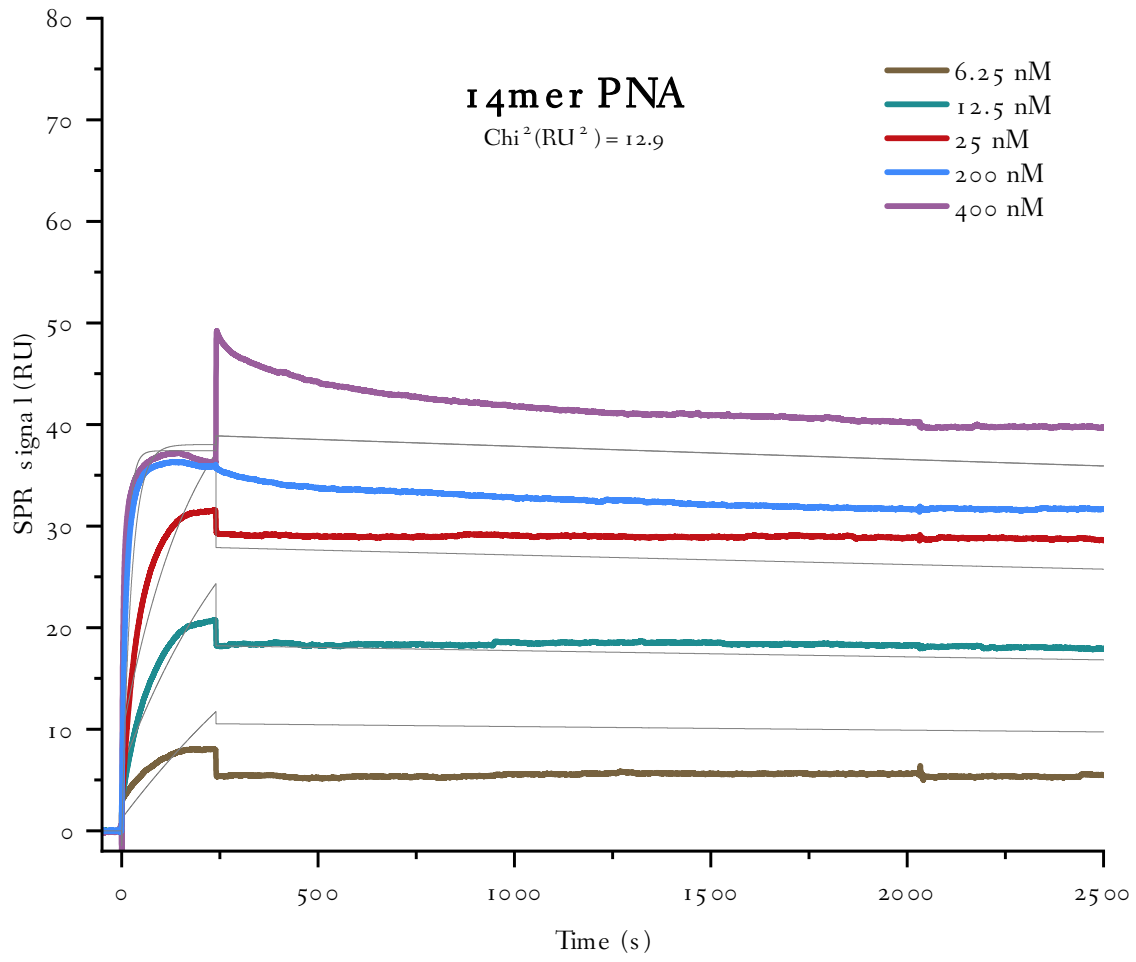

14mer PNA
$Chi^2 (RU^2) = 12.9$
6.25 nM
12.5 nM
25 nM
200 nM
400 nM
SPR signal (RU)
Time (s)
80
70
60
50
40
30
20
10
0
0
500
1000
1500
2000
2500

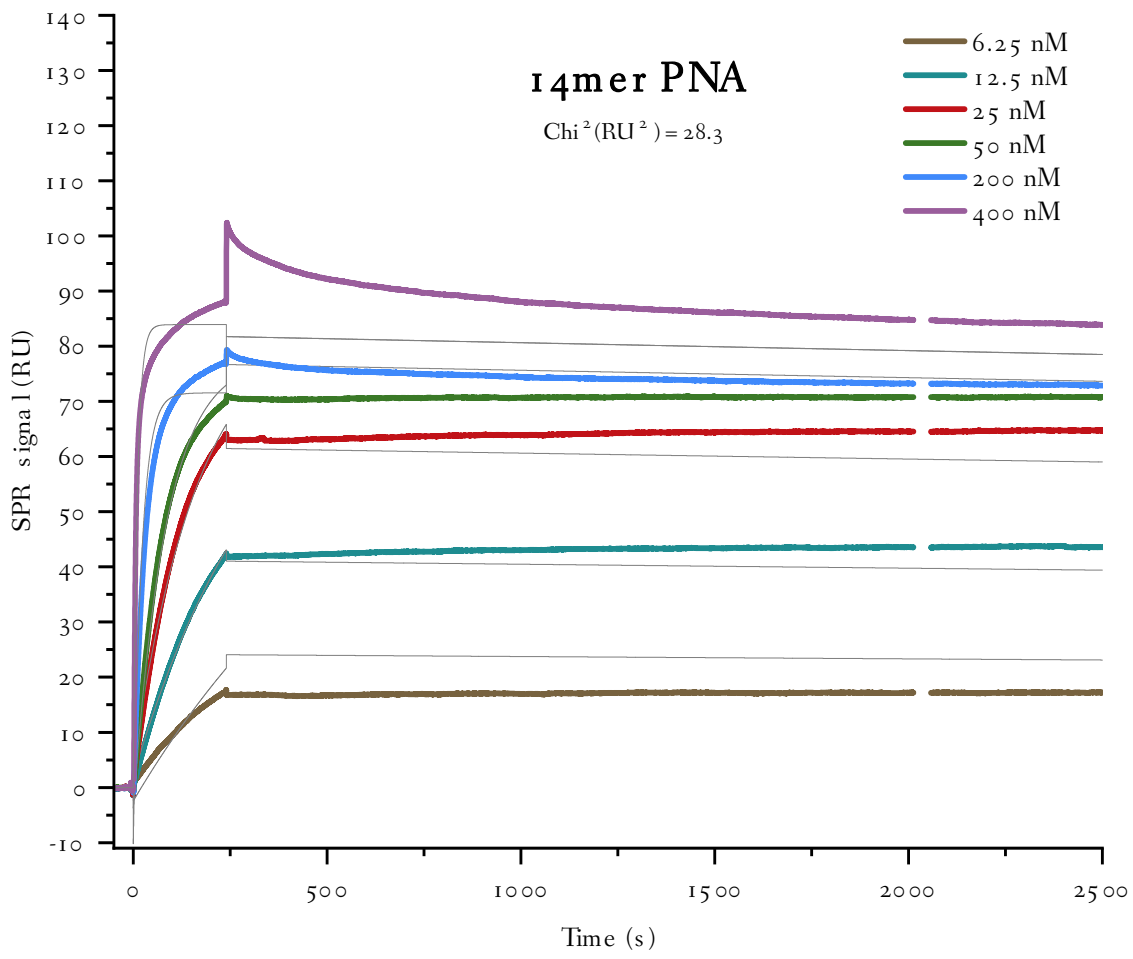

14mer PNA
$Chi^2 (RU^2) = 28.3$
6.25 nM
12.5 nM
25 nM
50 nM
200 nM
400 nM
SPR signal (RU)
Time (s)
140
130
120
110
100
90
80
70
60
50
40
30
20
10
0
-10
0
500
1000
1500
2000
2500

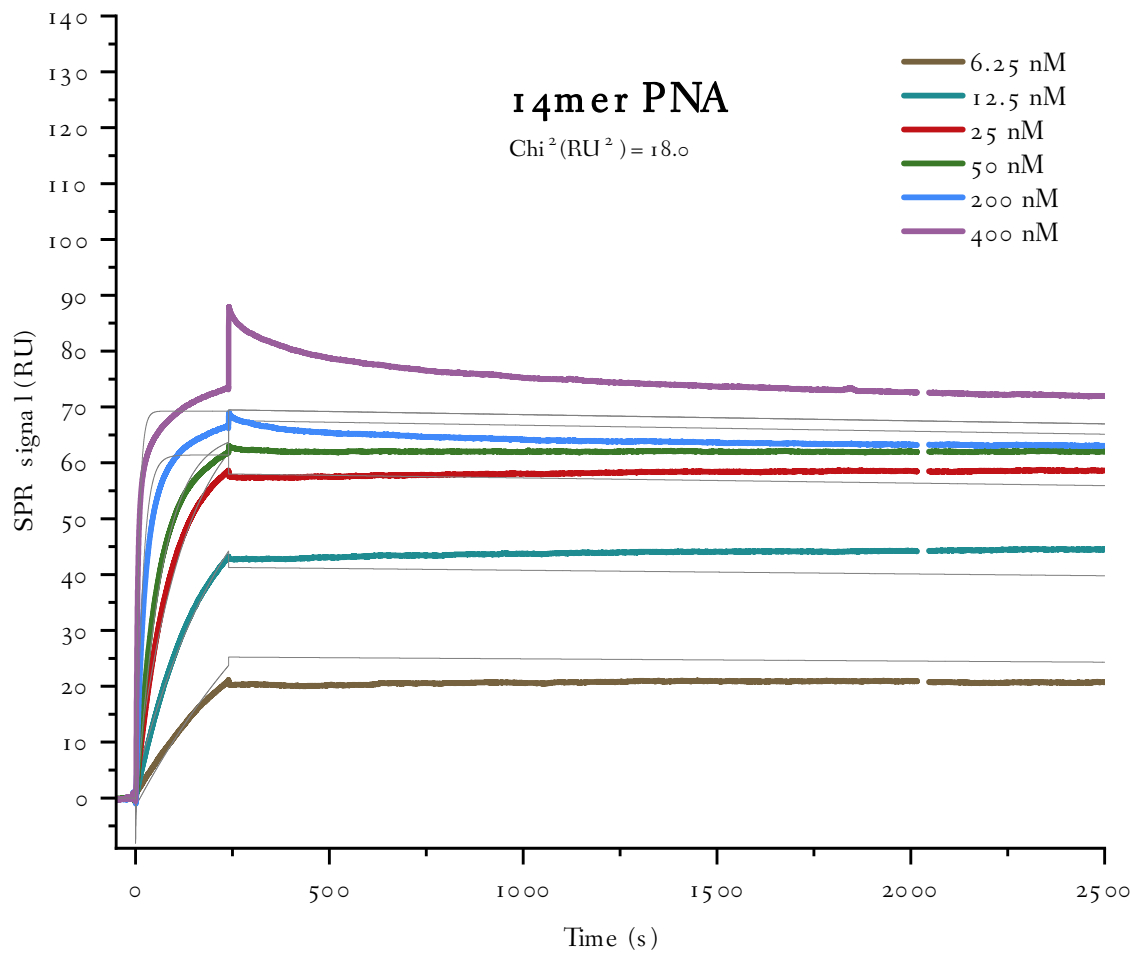

14mer PNA
$Chi^2 (RU^2) = 18.0$
6.25 nM
12.5 nM
25 nM
50 nM
200 nM
400 nM
SPR signal (RU)
Time (s)
140
130
120
110
100
90
80
70
60
50
40
30
20
10
0
0
500
1000
1500
2000
2500

5.8. 10mer mp-PNA

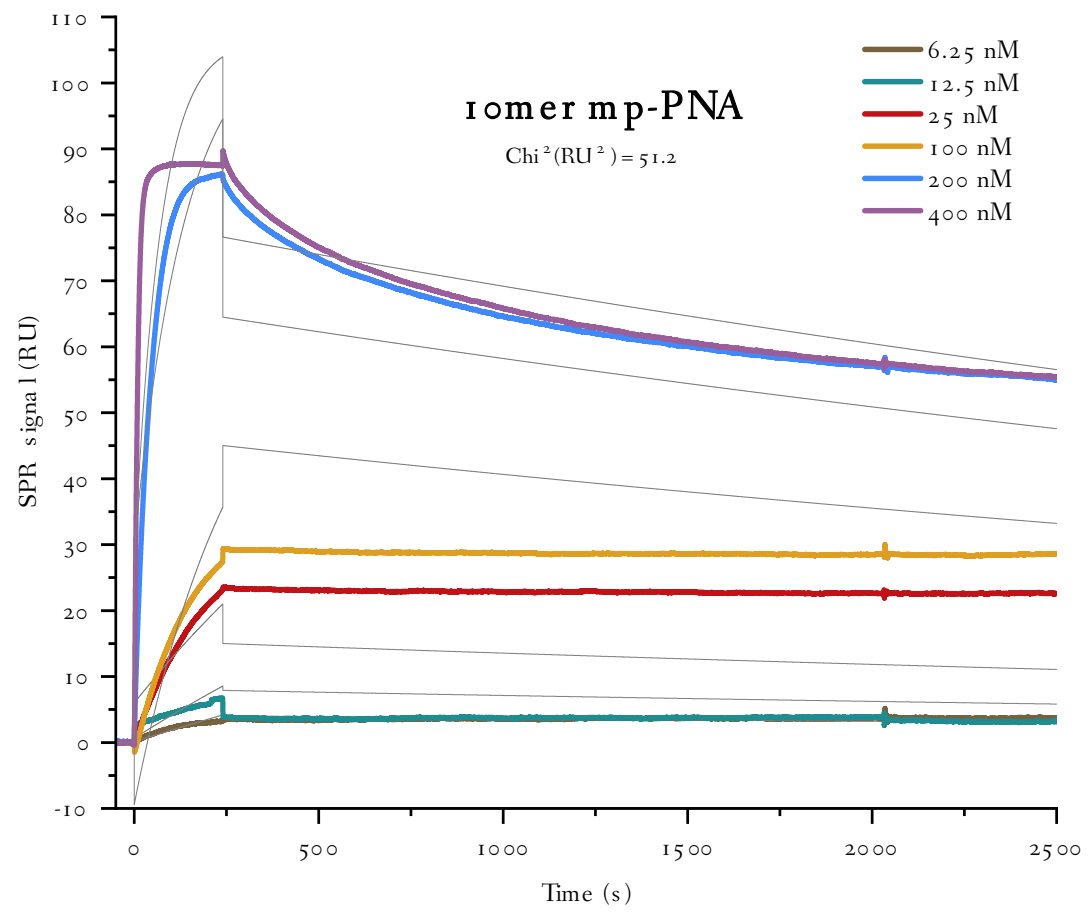
10mer mp-PNA
Chi^2(RU^2) = 51.2
6.25 nM
12.5 nM
25 nM
100 nM
200 nM
400 nM
SPR signal (RU)
Time (s)

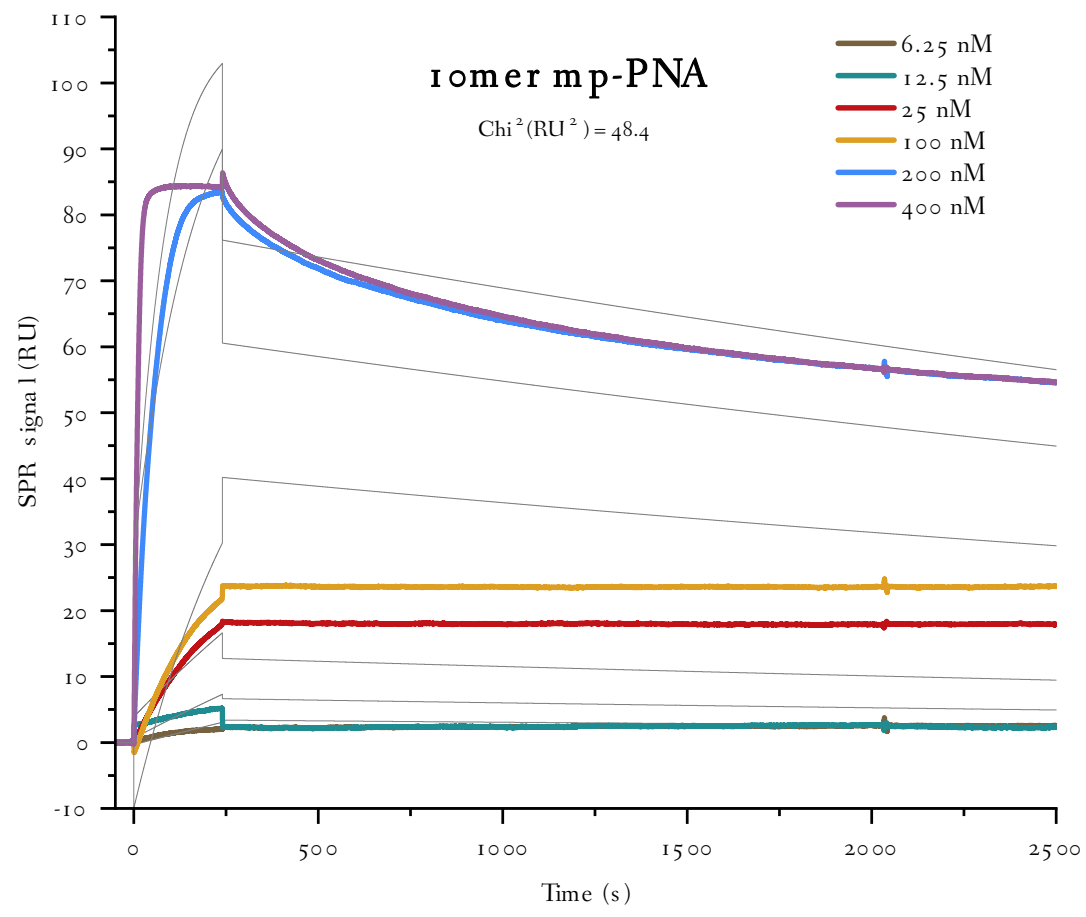
10mer mp-PNA
Chi^2(RU^2) = 48.4
6.25 nM
12.5 nM
25 nM
100 nM
200 nM
400 nM
SPR signal (RU)
Time (s)

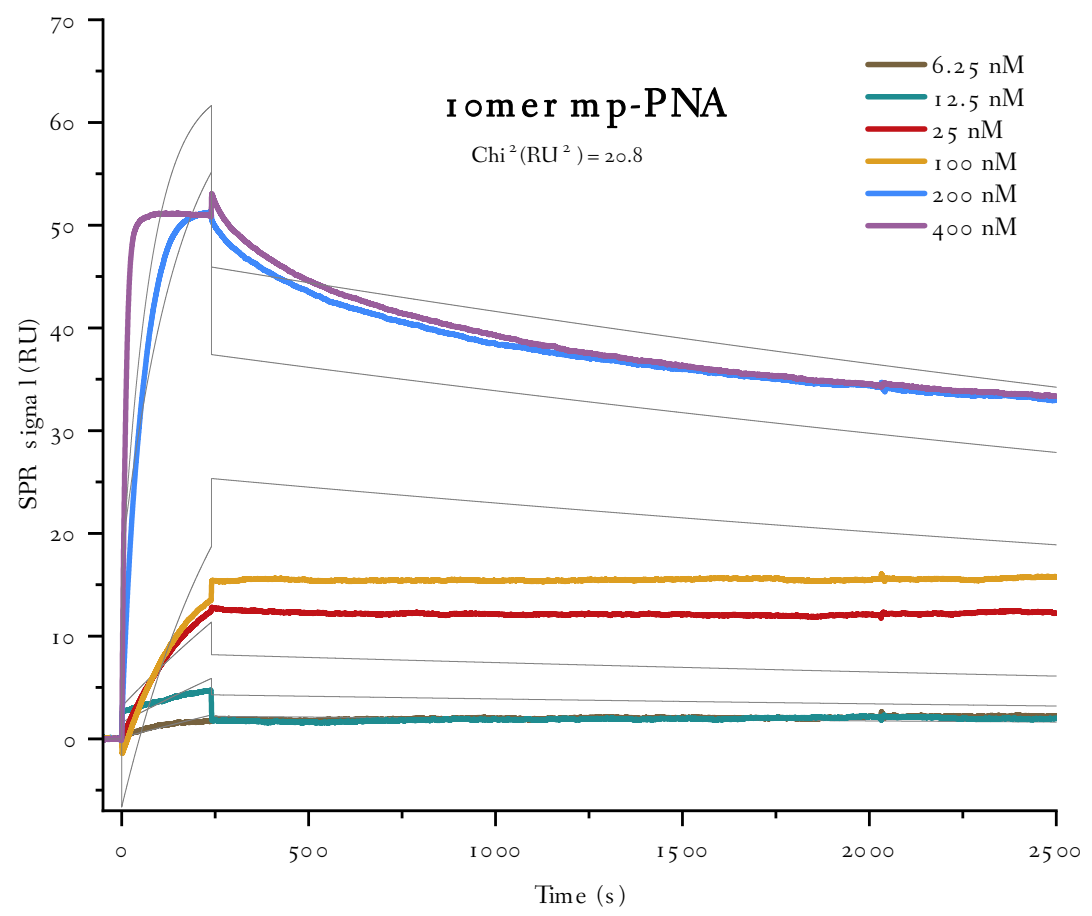
10mer mp-PNA
Chi^2(RU^2) = 20.8
6.25 nM
12.5 nM
25 nM
100 nM
200 nM
400 nM
SPR signal (RU)
Time (s)

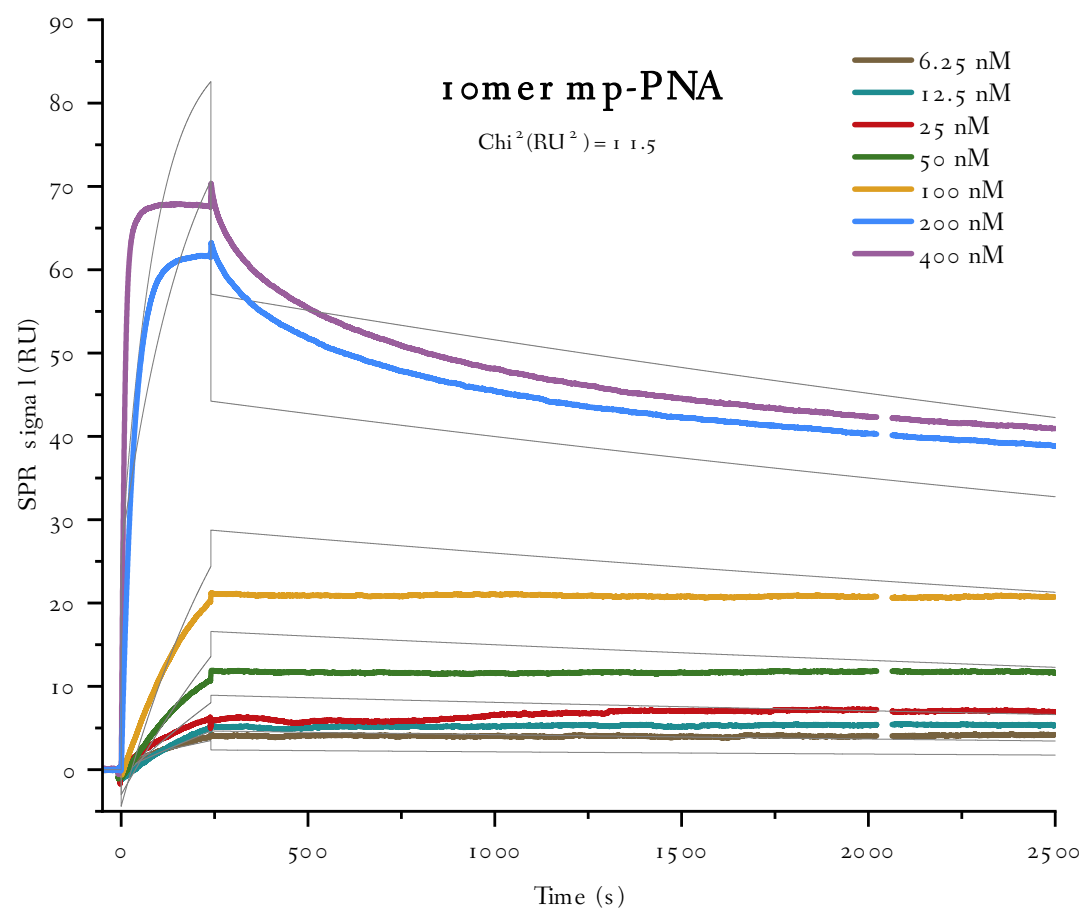
10mer mp-PNA
Chi^2(RU^2) = 11.5
6.25 nM
12.5 nM
25 nM
50 nM
100 nM
200 nM
400 nM
SPR signal (RU)
Time (s)

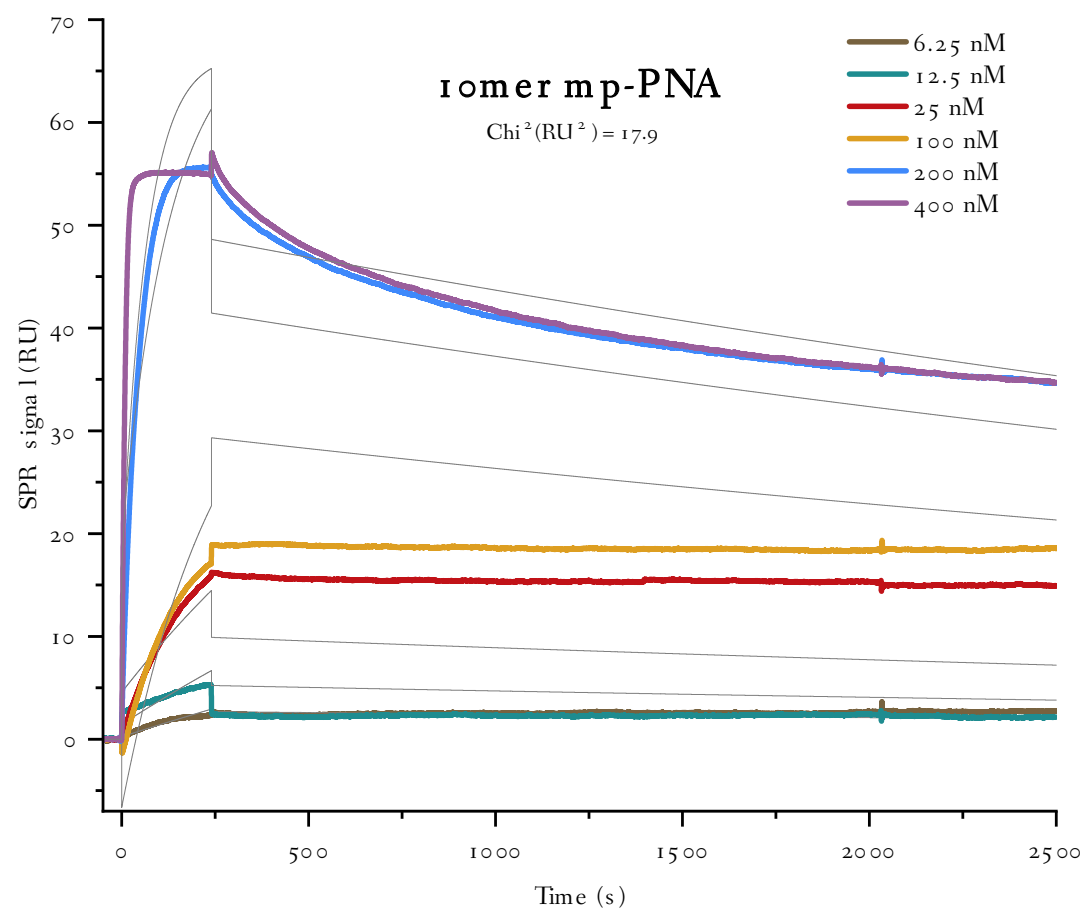
10mer mp-PNA
Chi^2(RU^2) = 17.9
6.25 nM
12.5 nM
25 nM
100 nM
200 nM
400 nM
SPR signal (RU)
Time (s)

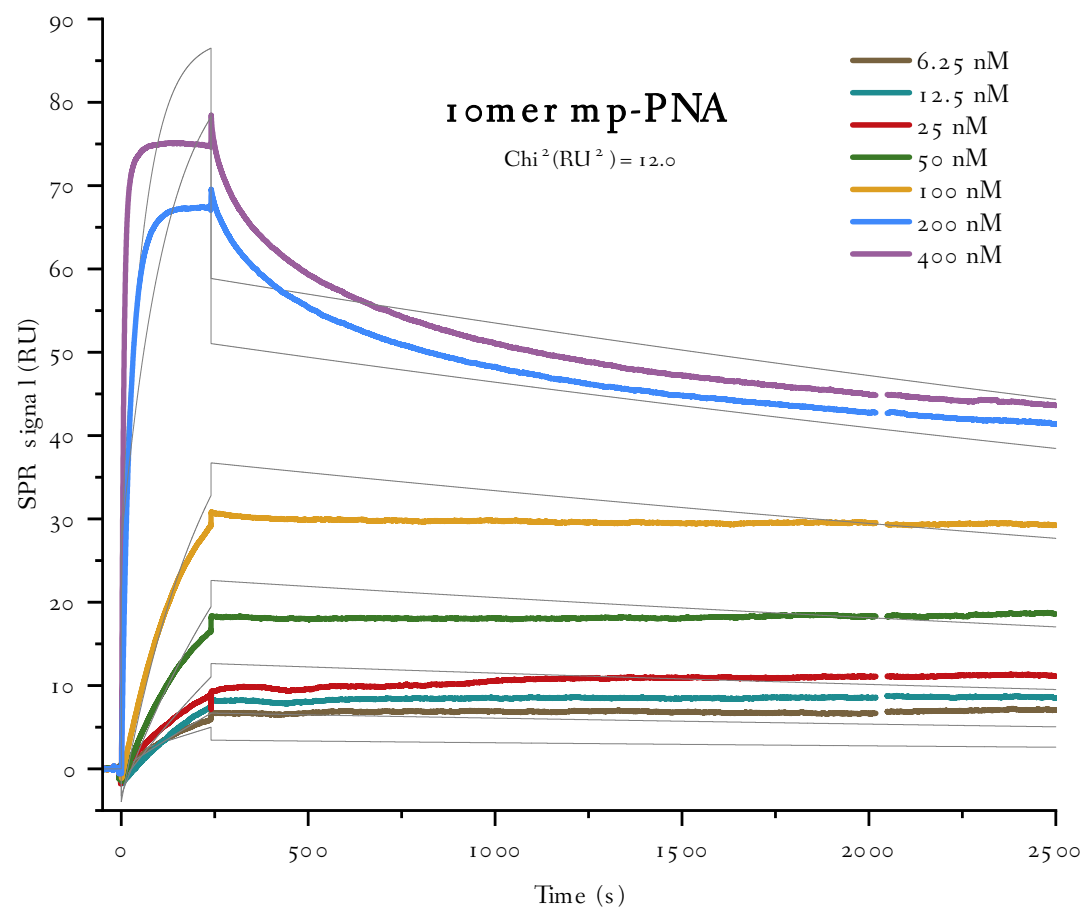
10mer mp-PNA
Chi^2(RU^2) = 12.0
6.25 nM
12.5 nM
25 nM
50 nM
100 nM
200 nM
400 nM
SPR signal (RU)
Time (s)

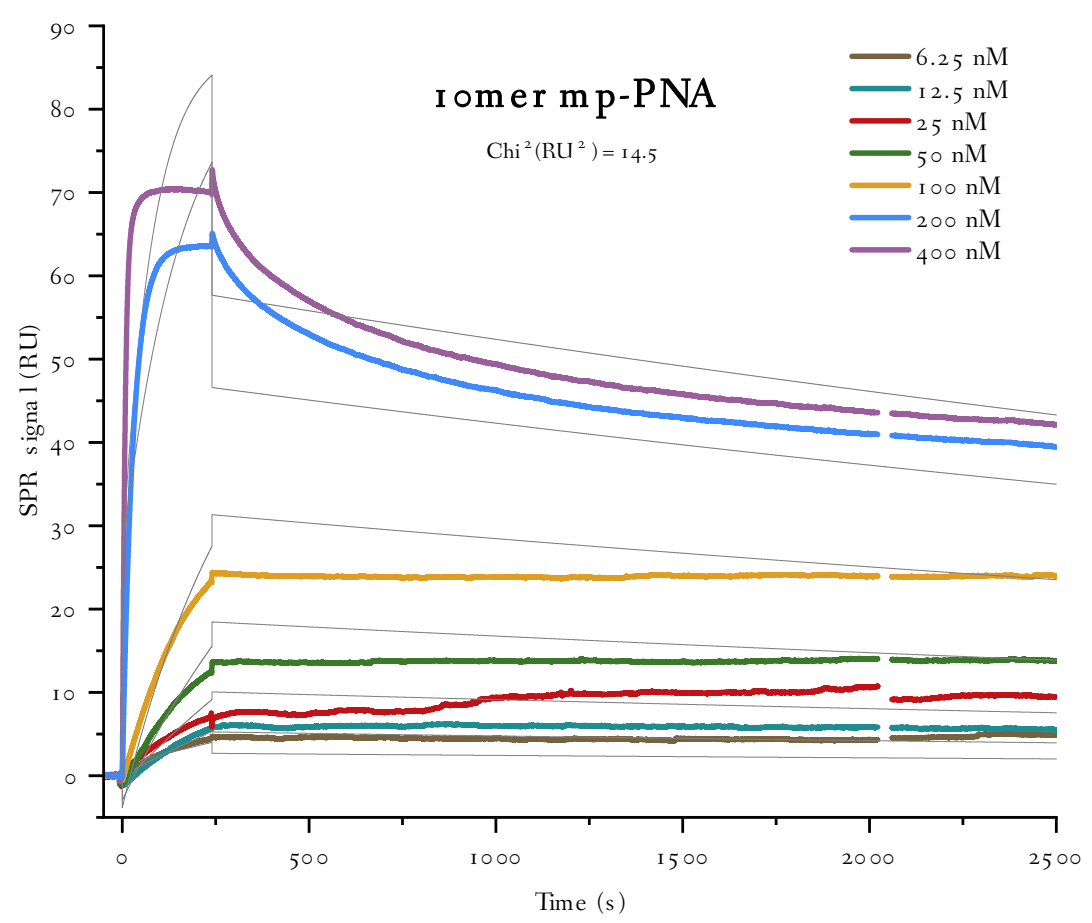
10mer mp-PNA
Chi^2(RU^2) = 14.5
6.25 nM
12.5 nM
25 nM
50 nM
100 nM
200 nM
400 nM
SPR signal (RU)
Time (s)

5.9. 12mer mp-PNA

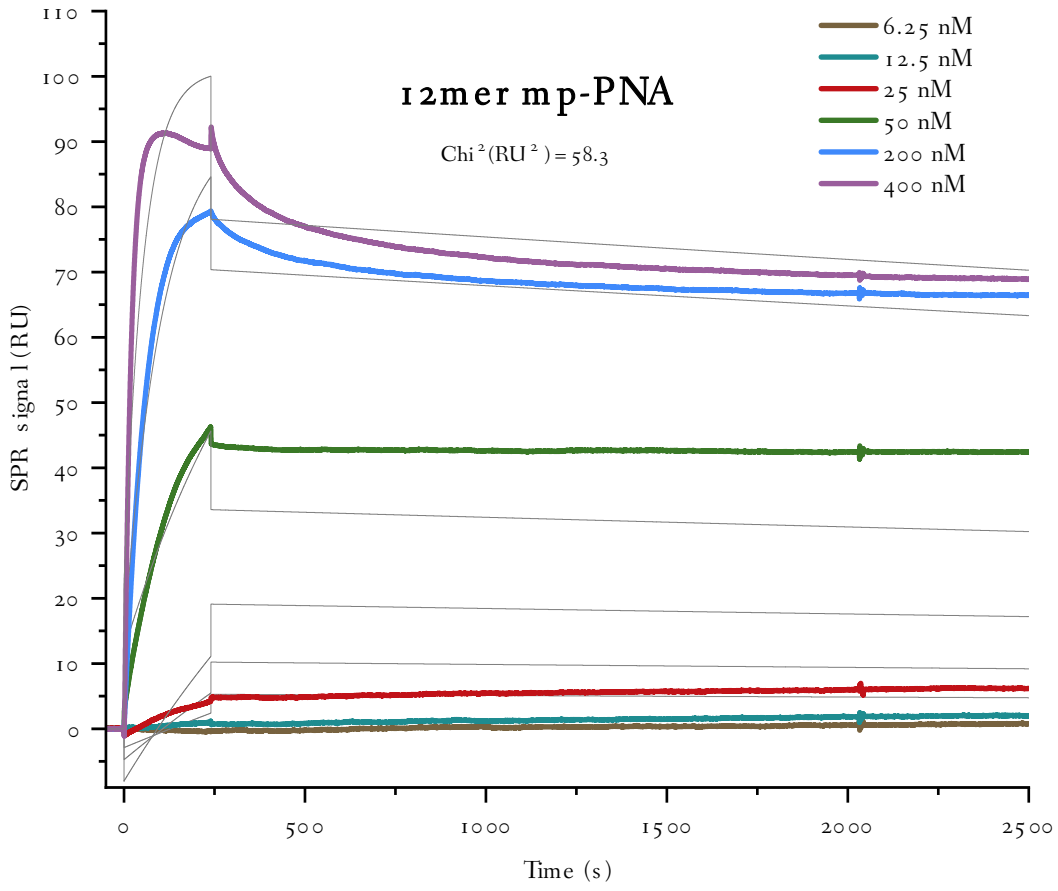

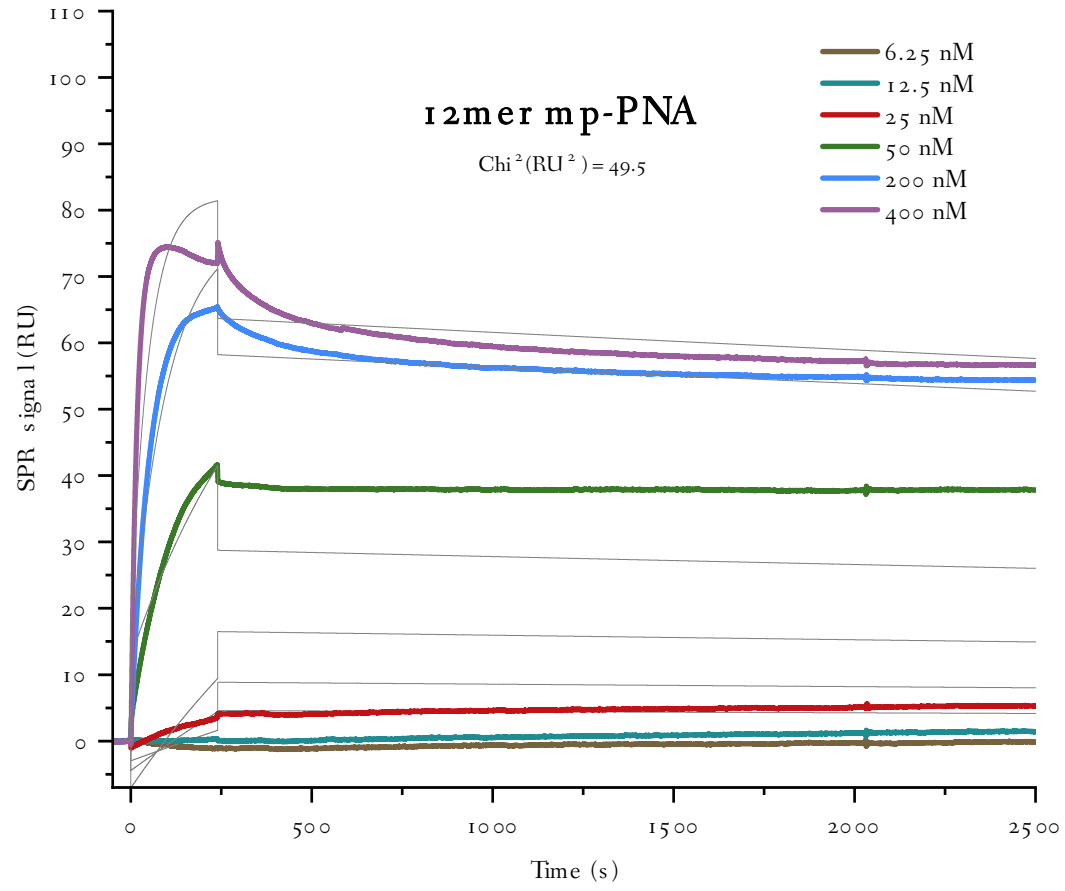

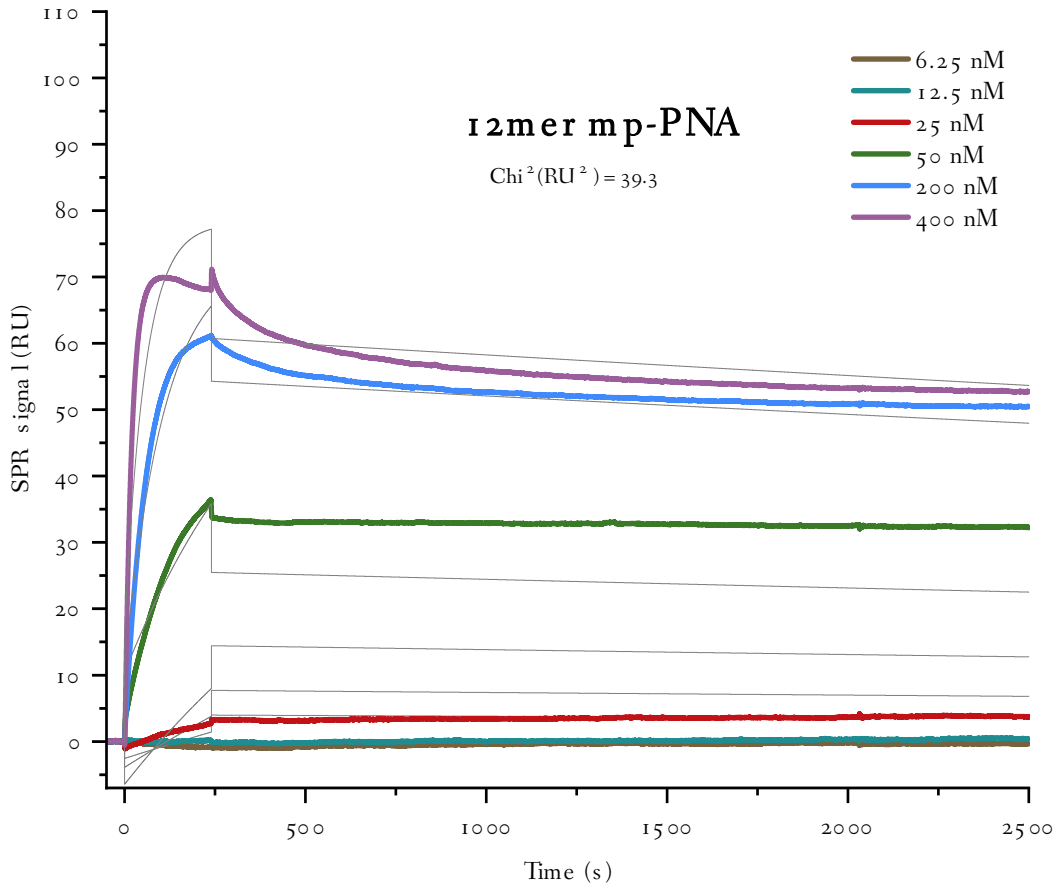

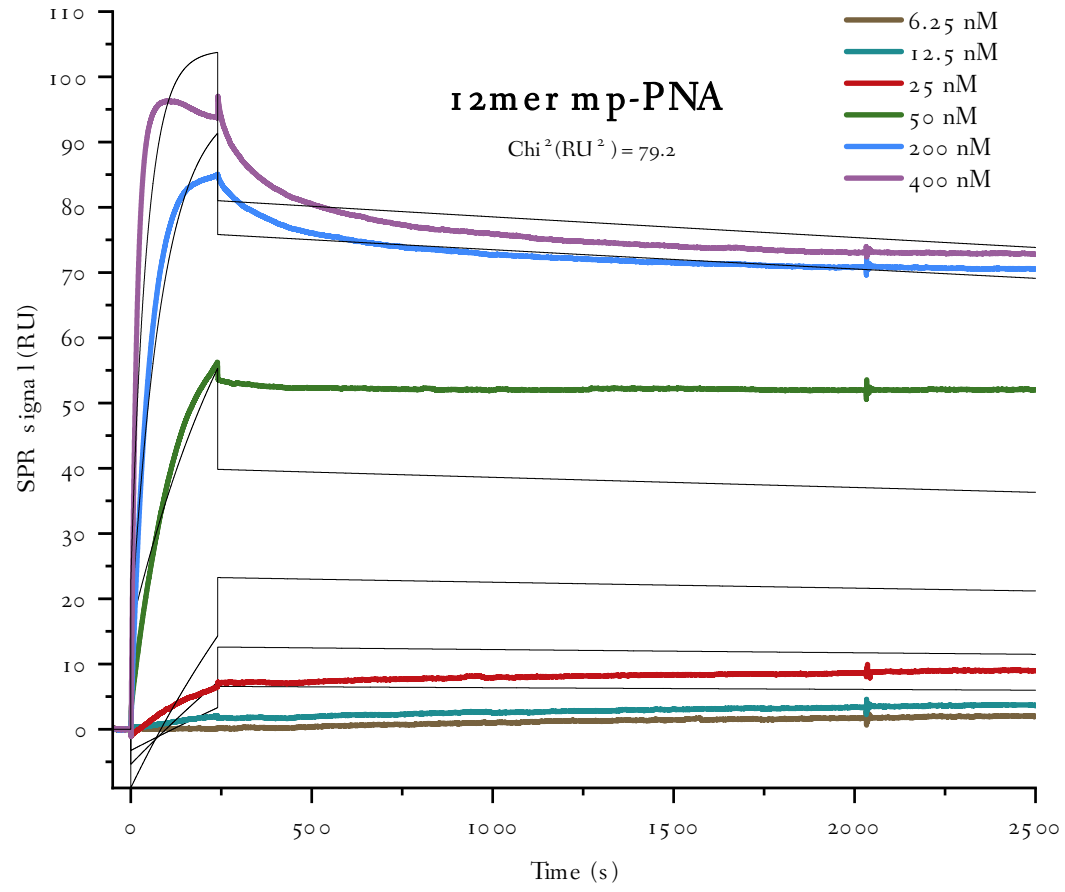

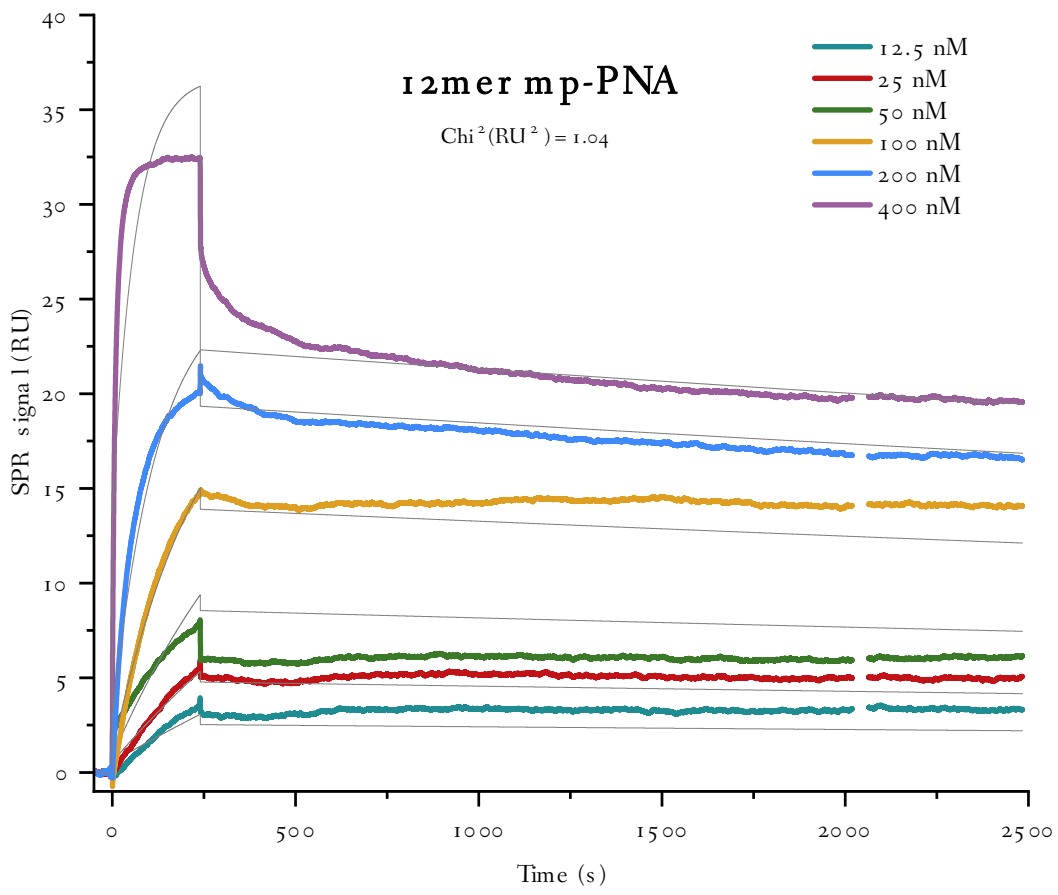

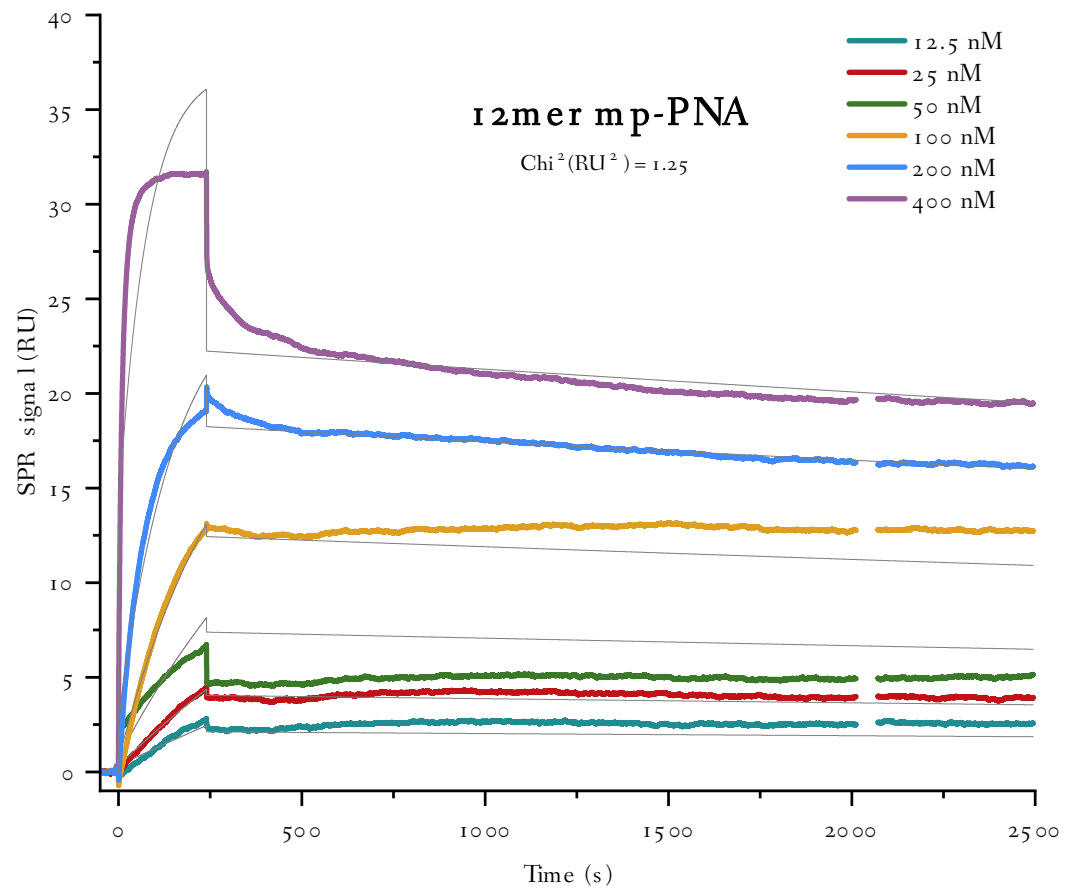

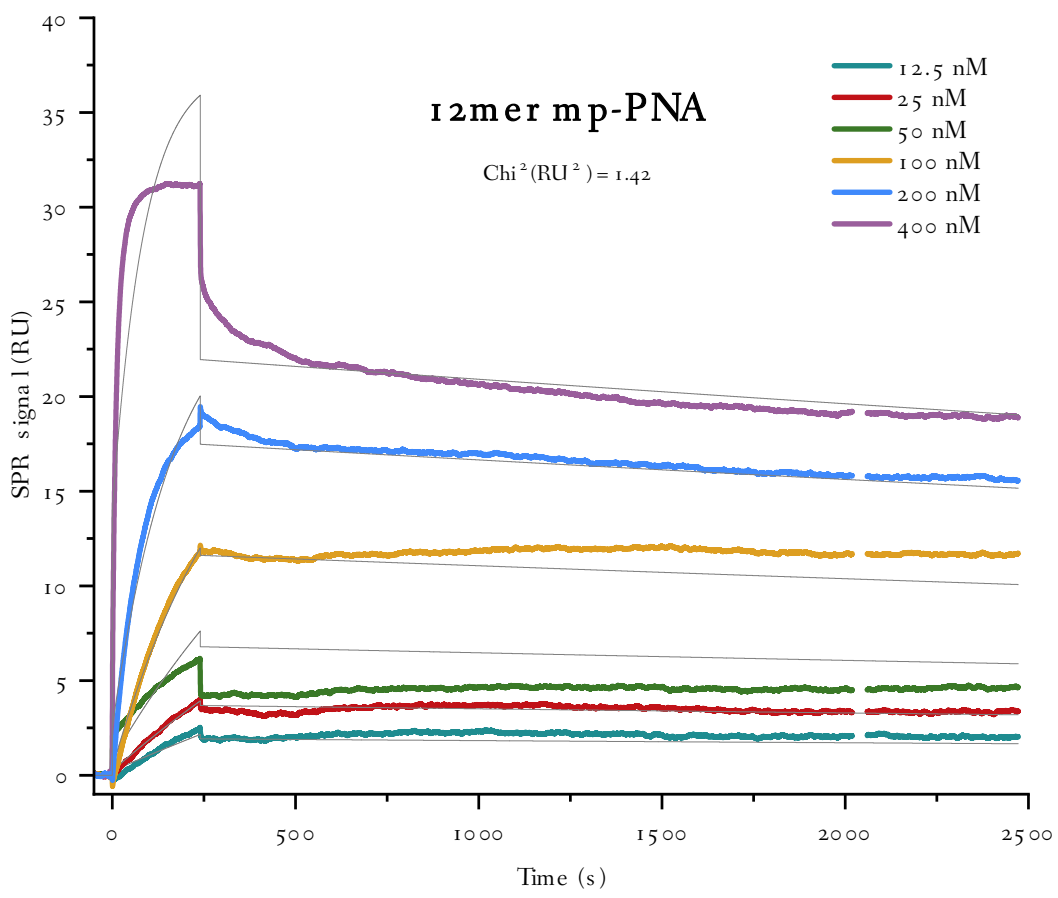

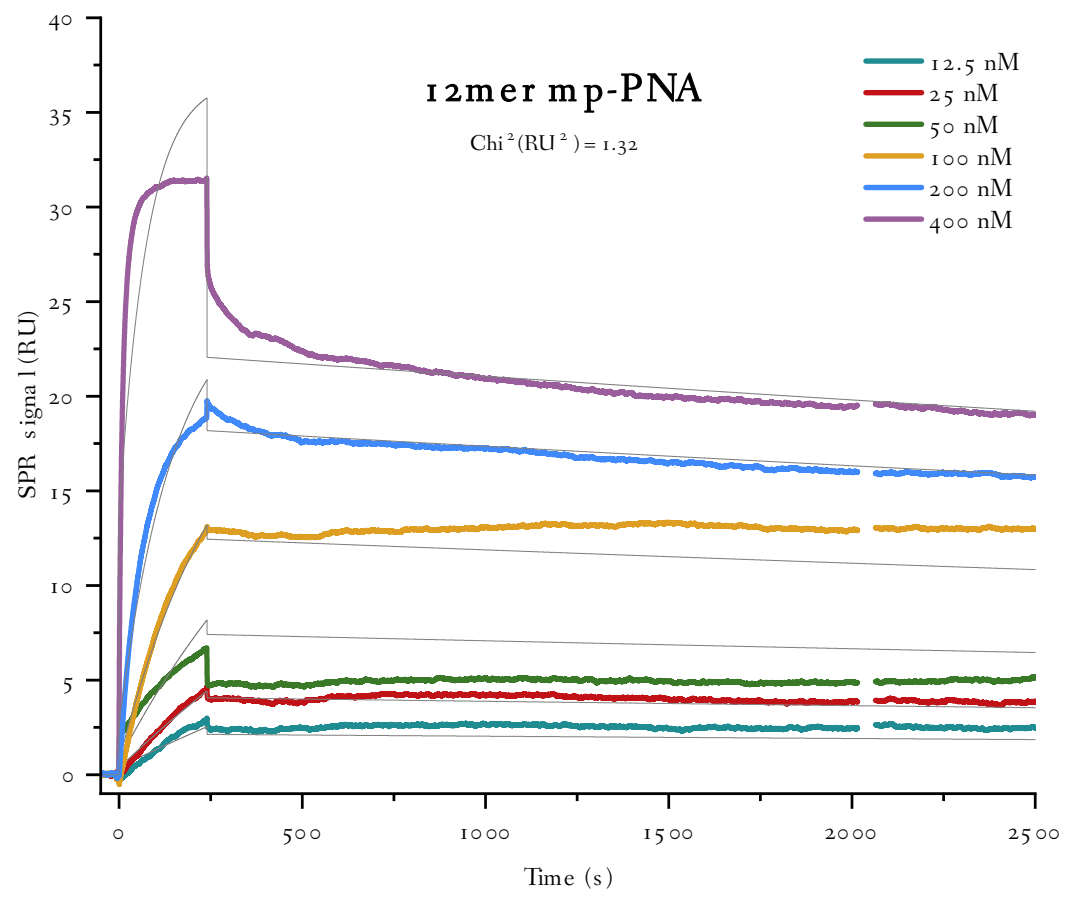

5.10. 14mer mp-PNA

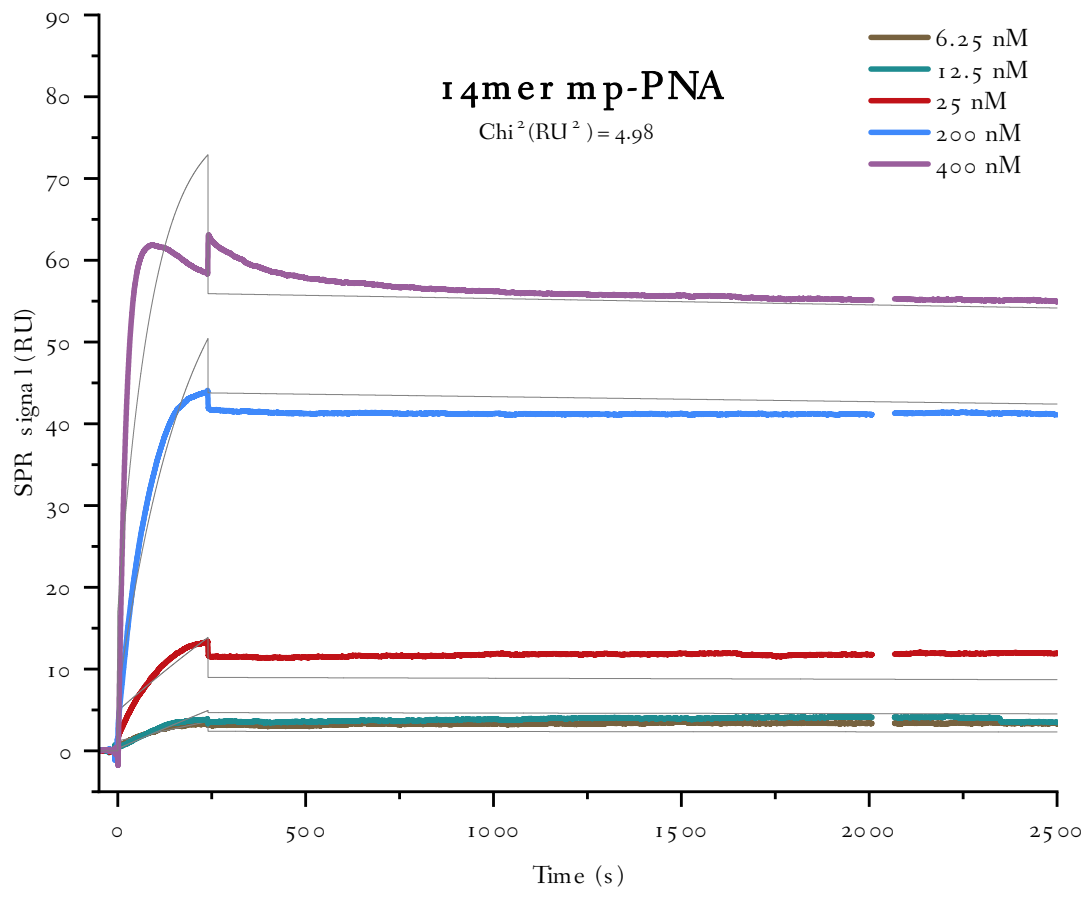
14mer mp-PNA
Chi²(RU²) = 4.98
6.25 nM
12.5 nM
25 nM
200 nM
400 nM
SPR signal (RU)
Time (s)

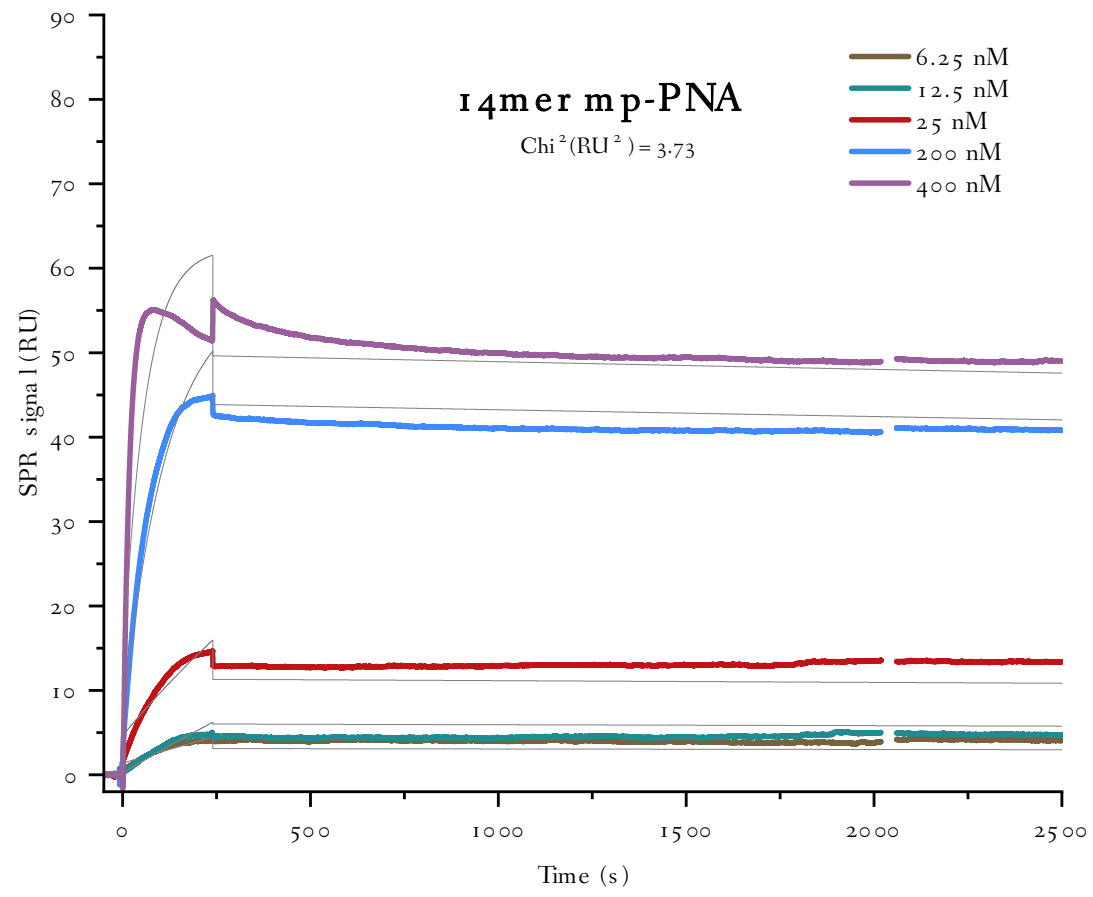
14mer mp-PNA
Chi²(RU²) = 3.73
6.25 nM
12.5 nM
25 nM
200 nM
400 nM
SPR signal (RU)
Time (s)

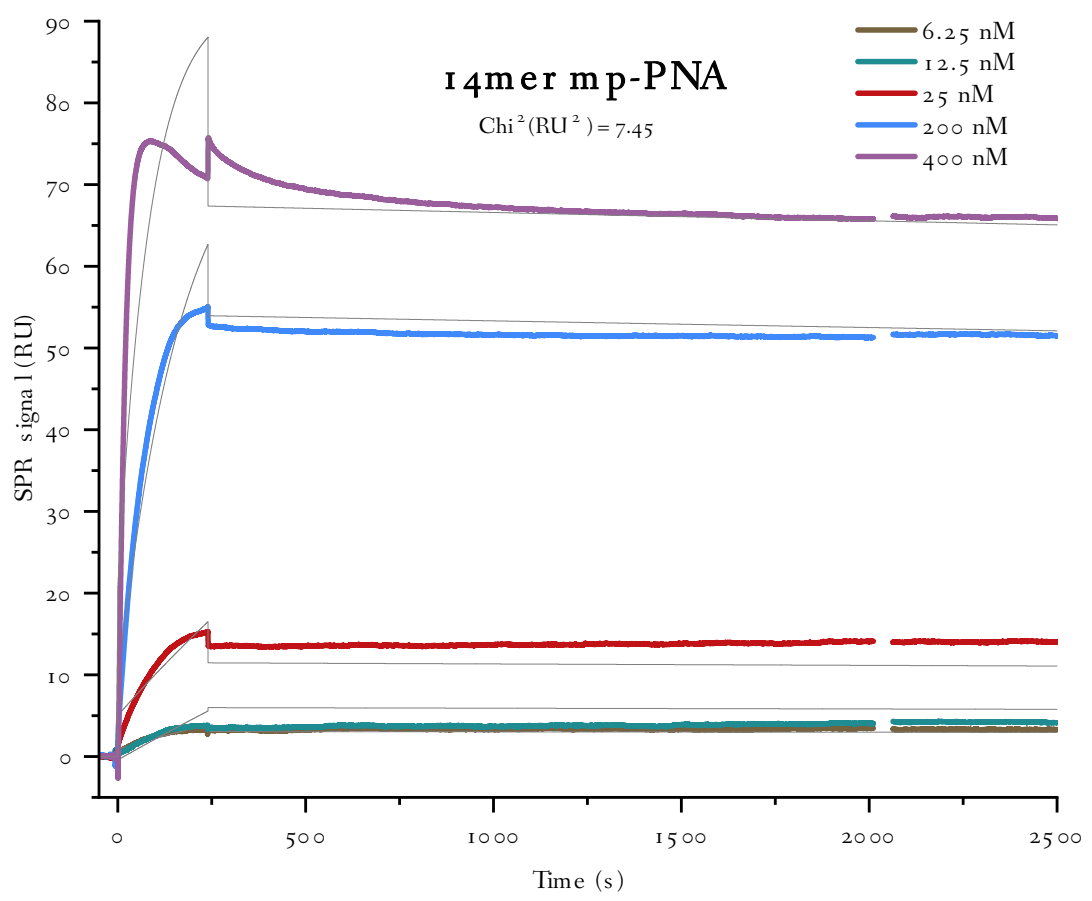
14mer mp-PNA
Chi²(RU²) = 7.45
6.25 nM
12.5 nM
25 nM
200 nM
400 nM
SPR signal (RU)
Time (s)

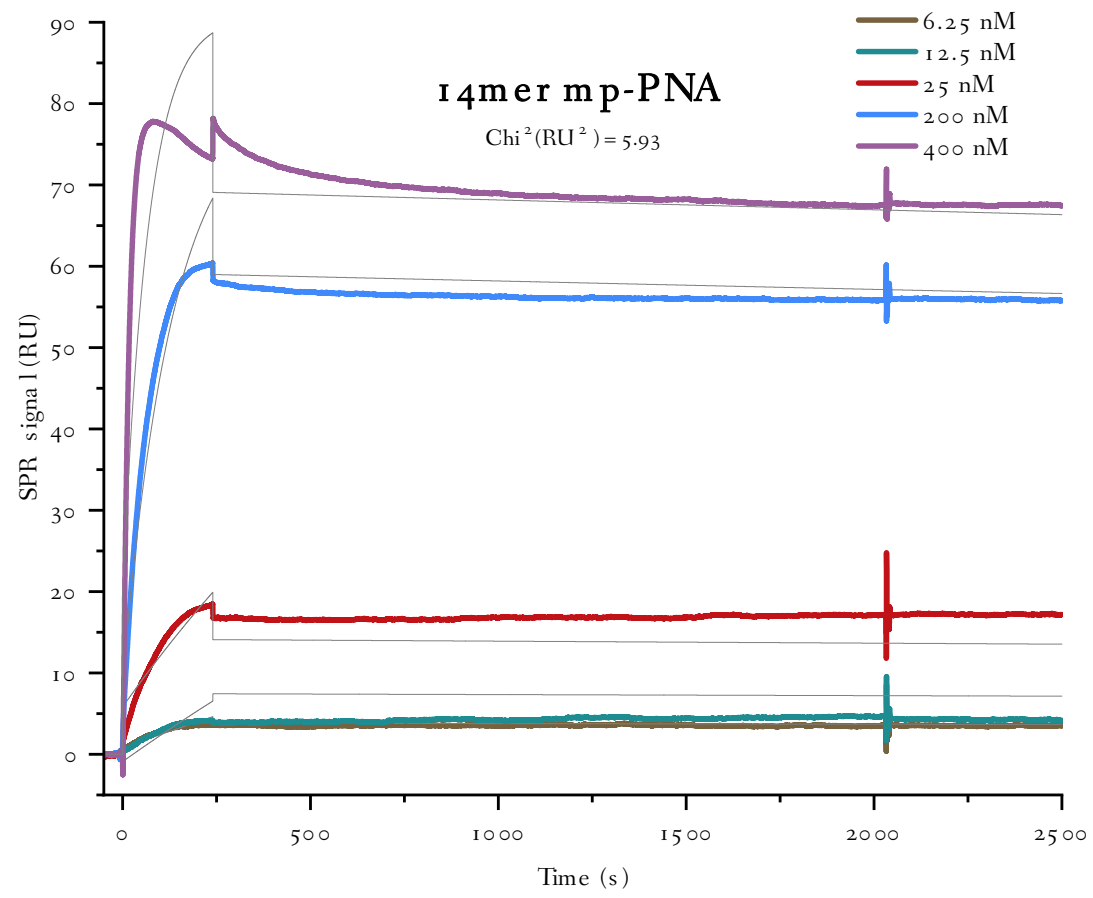
14mer mp-PNA
Chi²(RU²) = 5.93
6.25 nM
12.5 nM
25 nM
200 nM
400 nM
SPR signal (RU)
Time (s)

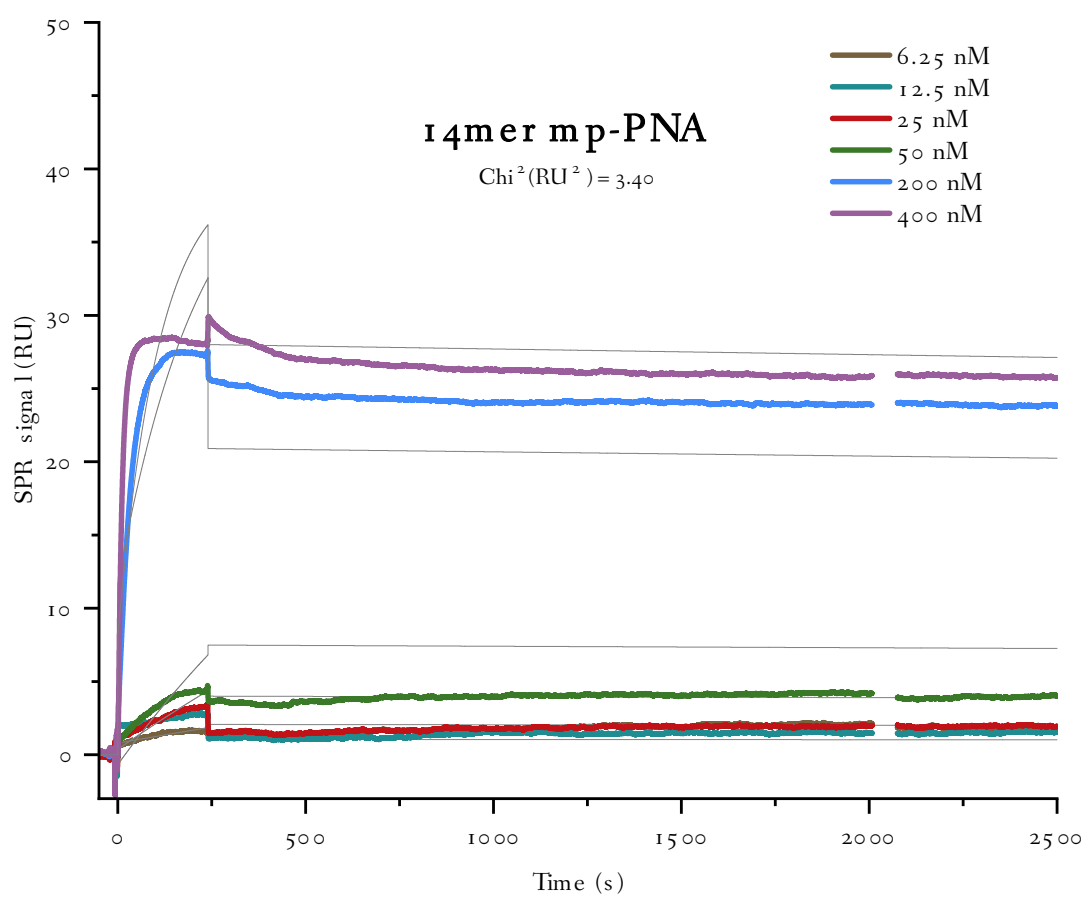
14mer mp-PNA
Chi²(RU²) = 3.40
6.25 nM
12.5 nM
25 nM
50 nM
200 nM
400 nM
SPR signal (RU)
Time (s)

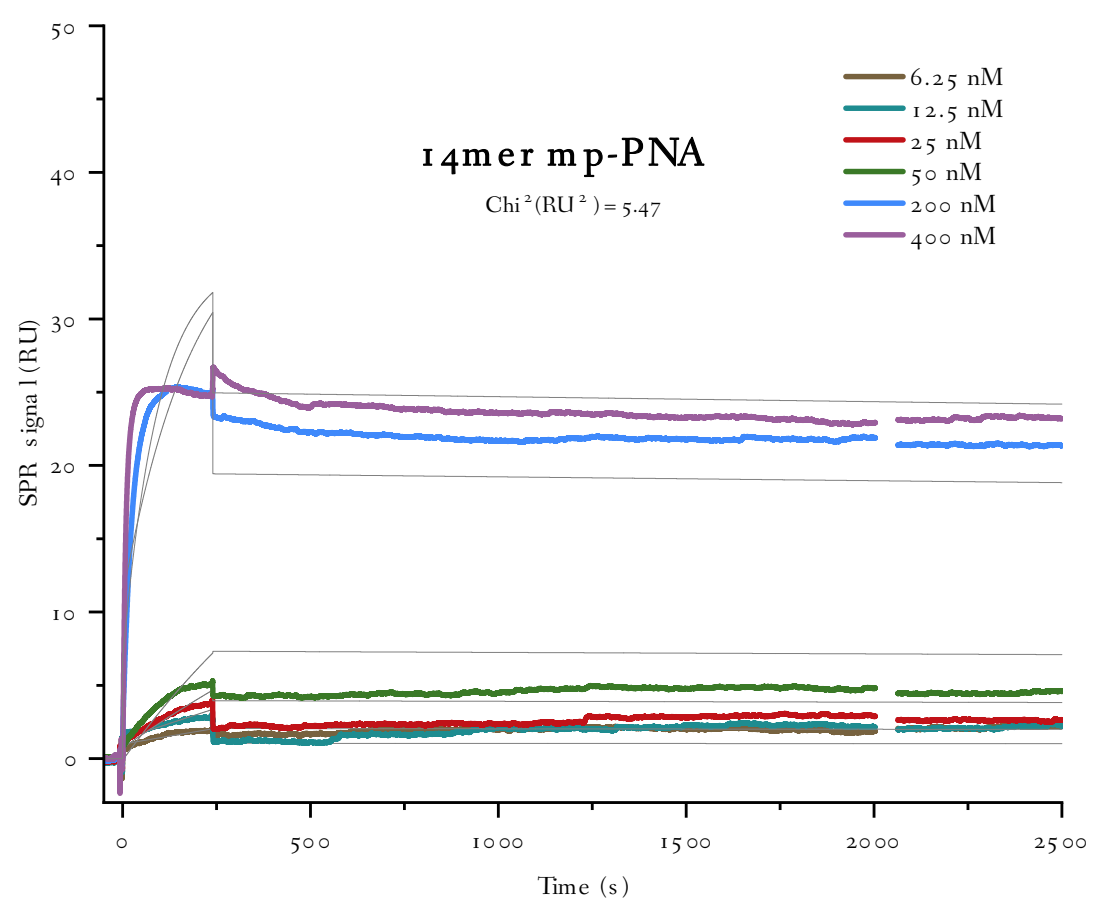
14mer mp-PNA
Chi²(RU²) = 5.47
6.25 nM
12.5 nM
25 nM
50 nM
200 nM
400 nM
SPR signal (RU)
Time (s)

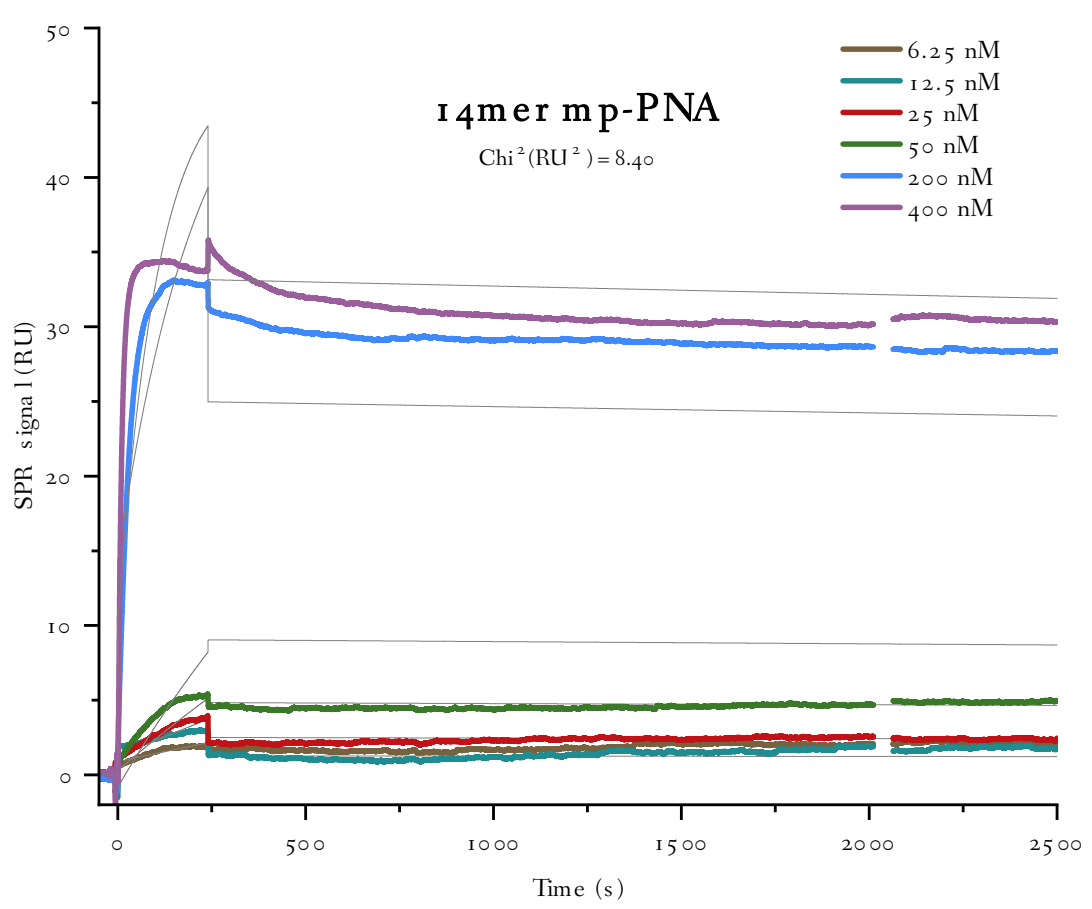
14mer mp-PNA
Chi²(RU²) = 8.40
6.25 nM
12.5 nM
25 nM
50 nM
200 nM
400 nM
SPR signal (RU)
Time (s)

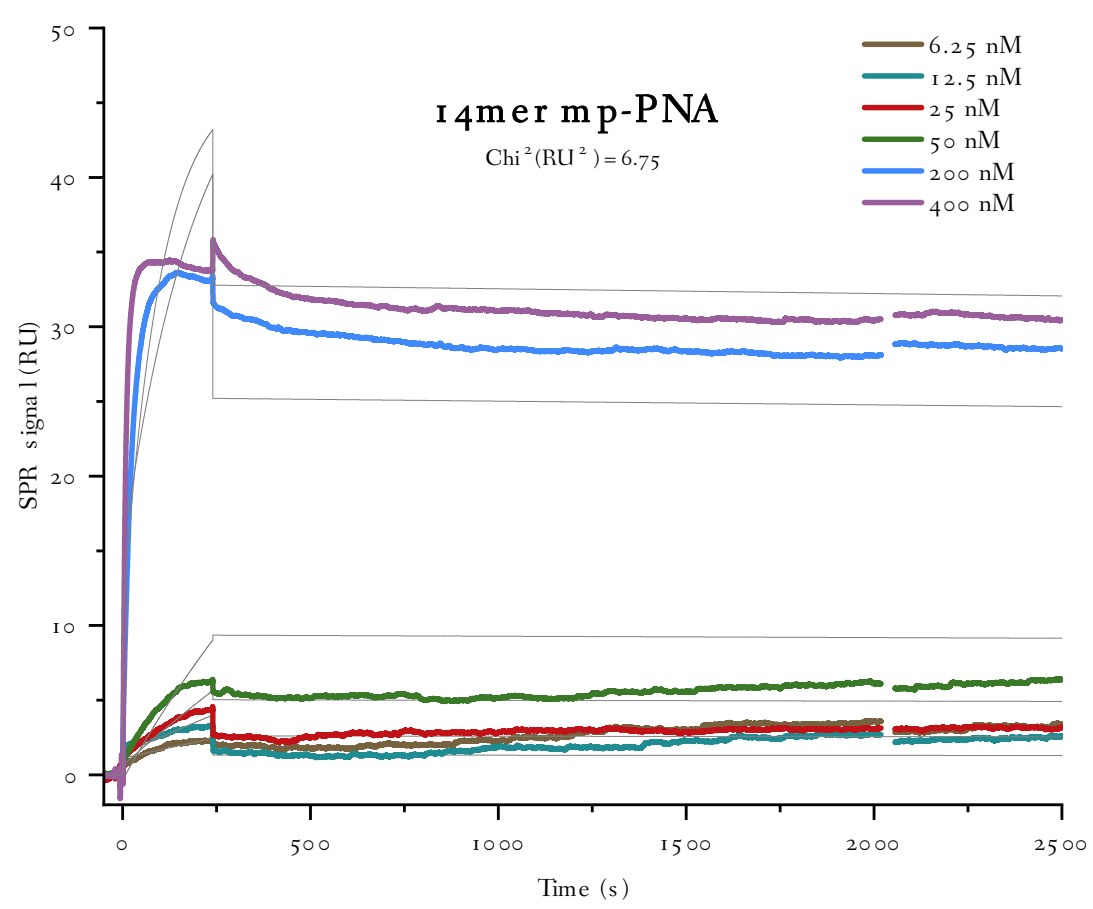
14mer mp-PNA
Chi²(RU²) = 6.75
6.25 nM
12.5 nM
25 nM
50 nM
200 nM
400 nM
SPR signal (RU)
Time (s)

5.11. 12mer PNA 37 °C

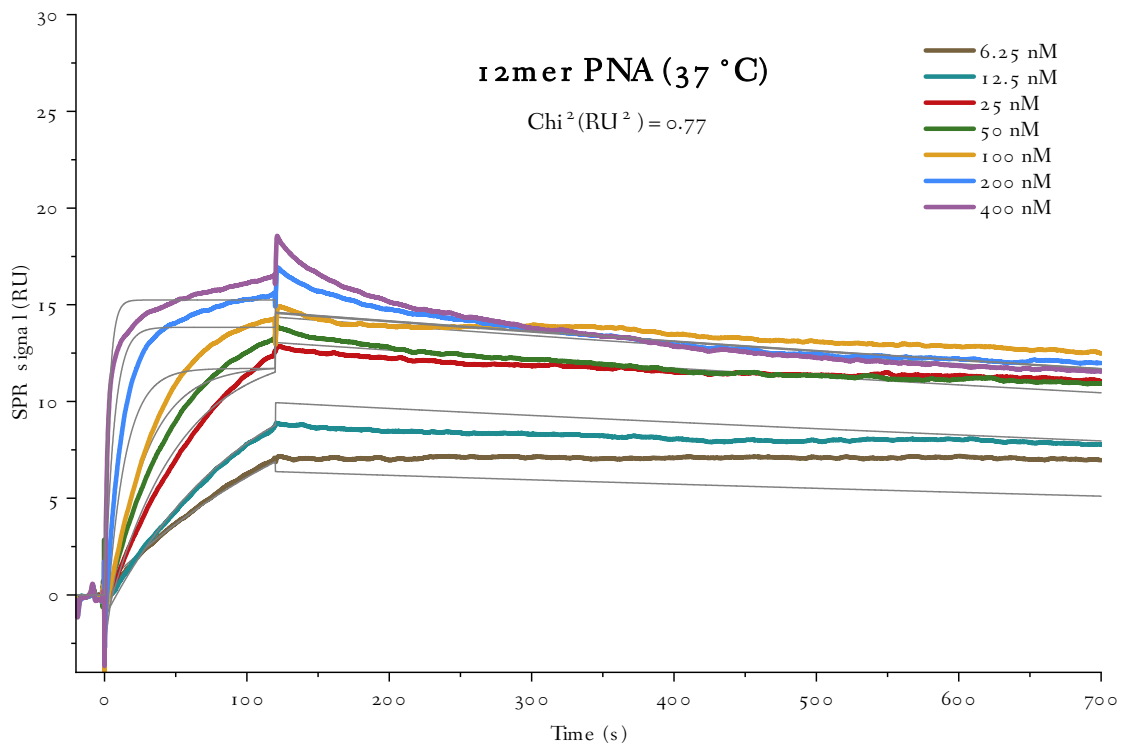

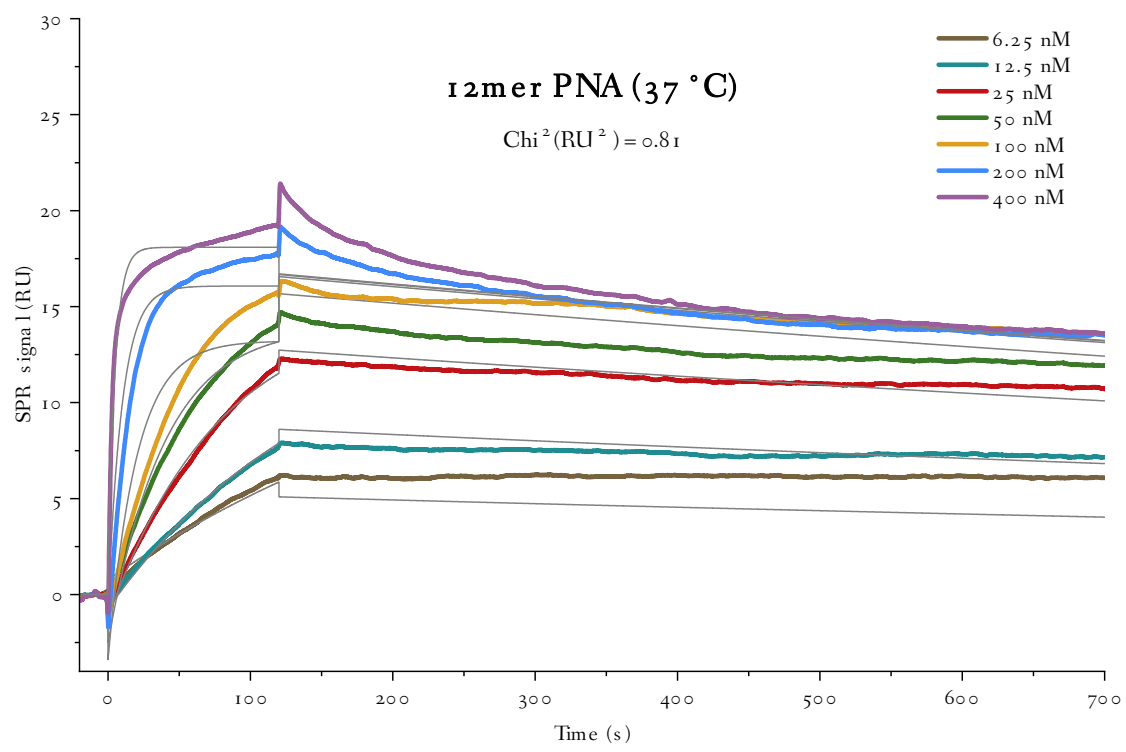

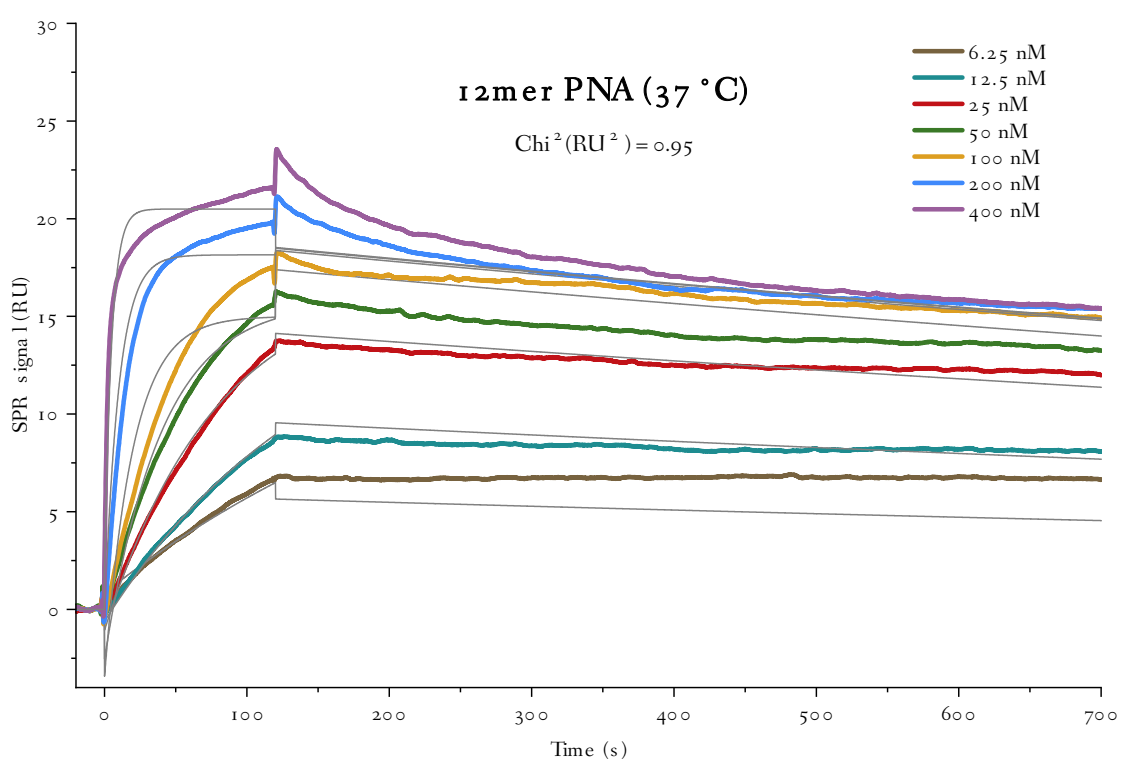

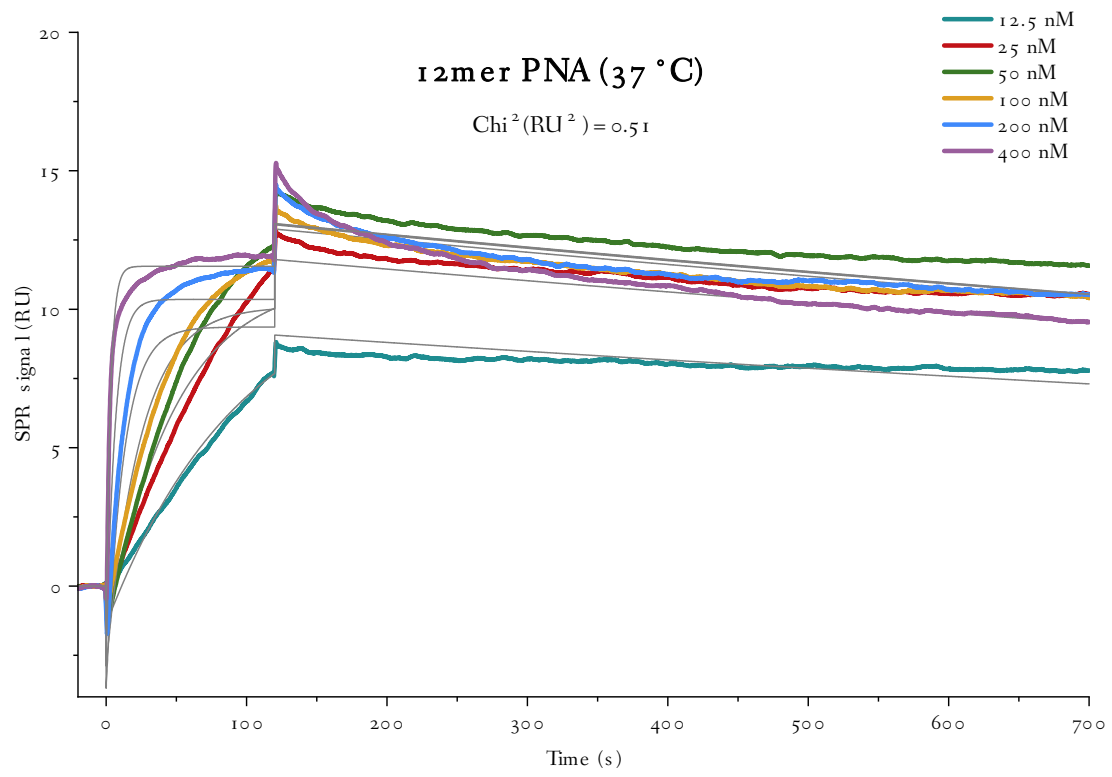

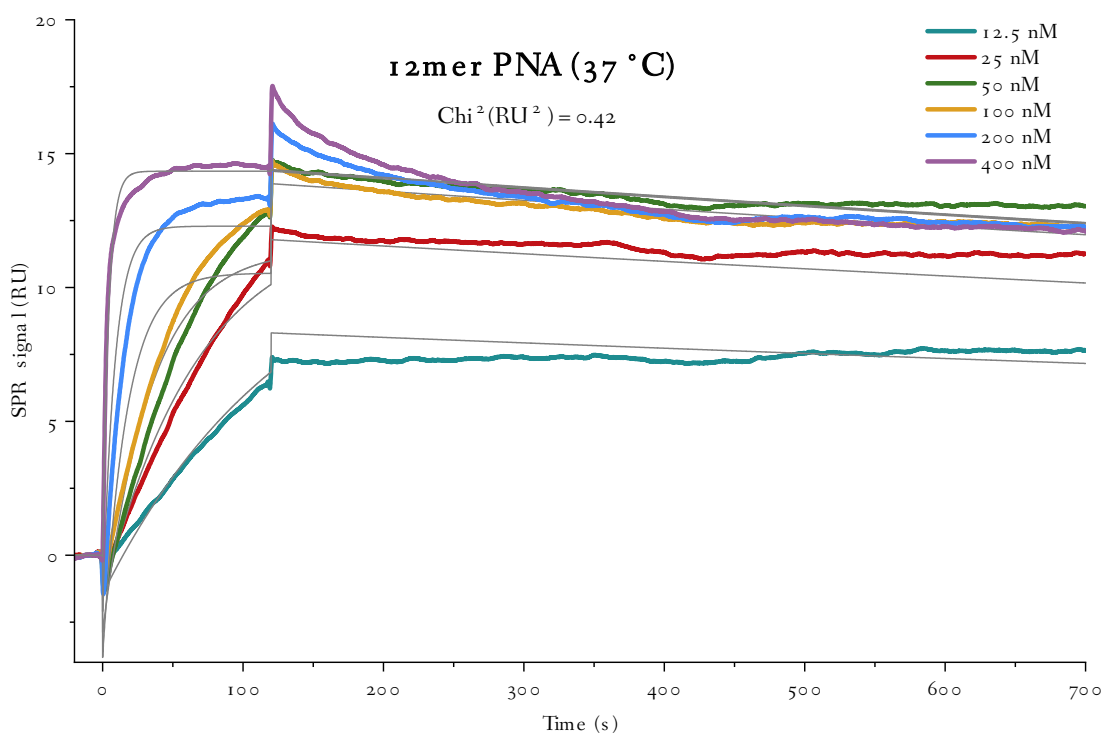

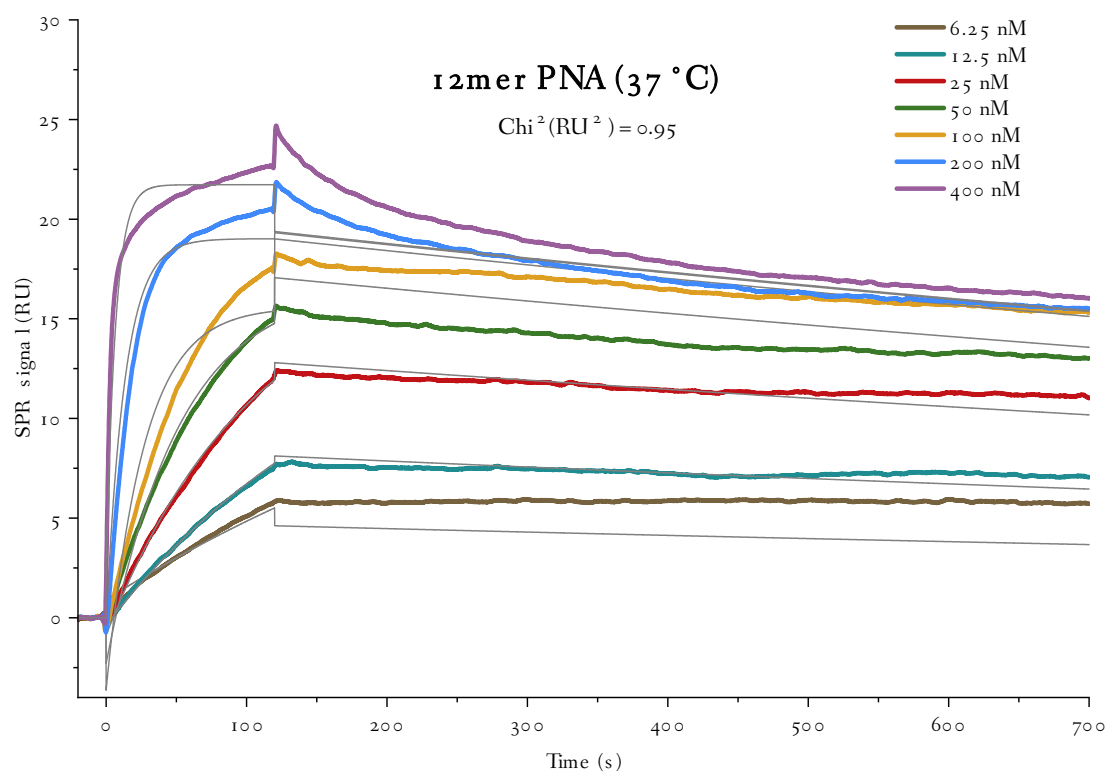

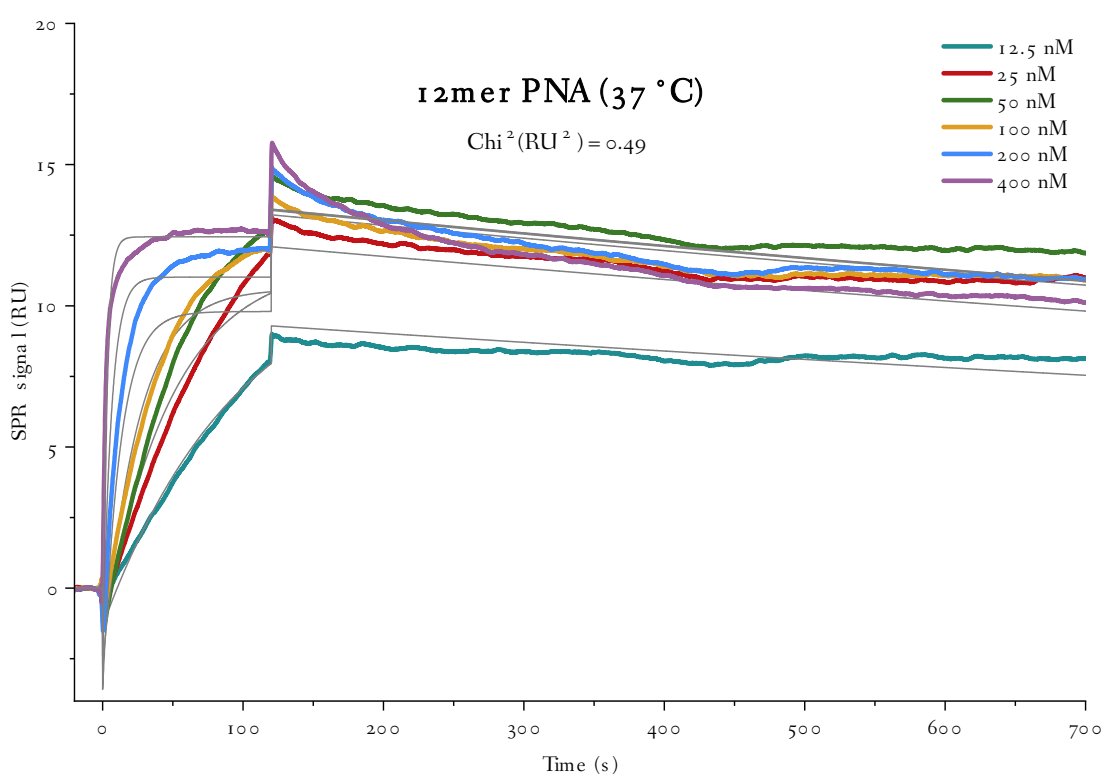

5.12. Control experiment using scrambled PNA

To demonstrate the specificity of the selected complementary strands and to exclude non-specific binding, a scrambled PNA (sc-PNA, H-ACT CTT TAT CGT-NH_2) was tested against the complementary 12mer PNA in control experiments. With three consecutive injections for each PNA, no hybridization of the scrambled PNA was observed, whereas the complementary 12mer PNA produced a clear SPR signal enhancement (~60 RU), confirming specific hybridization to the target RNA (SI Figure 4).

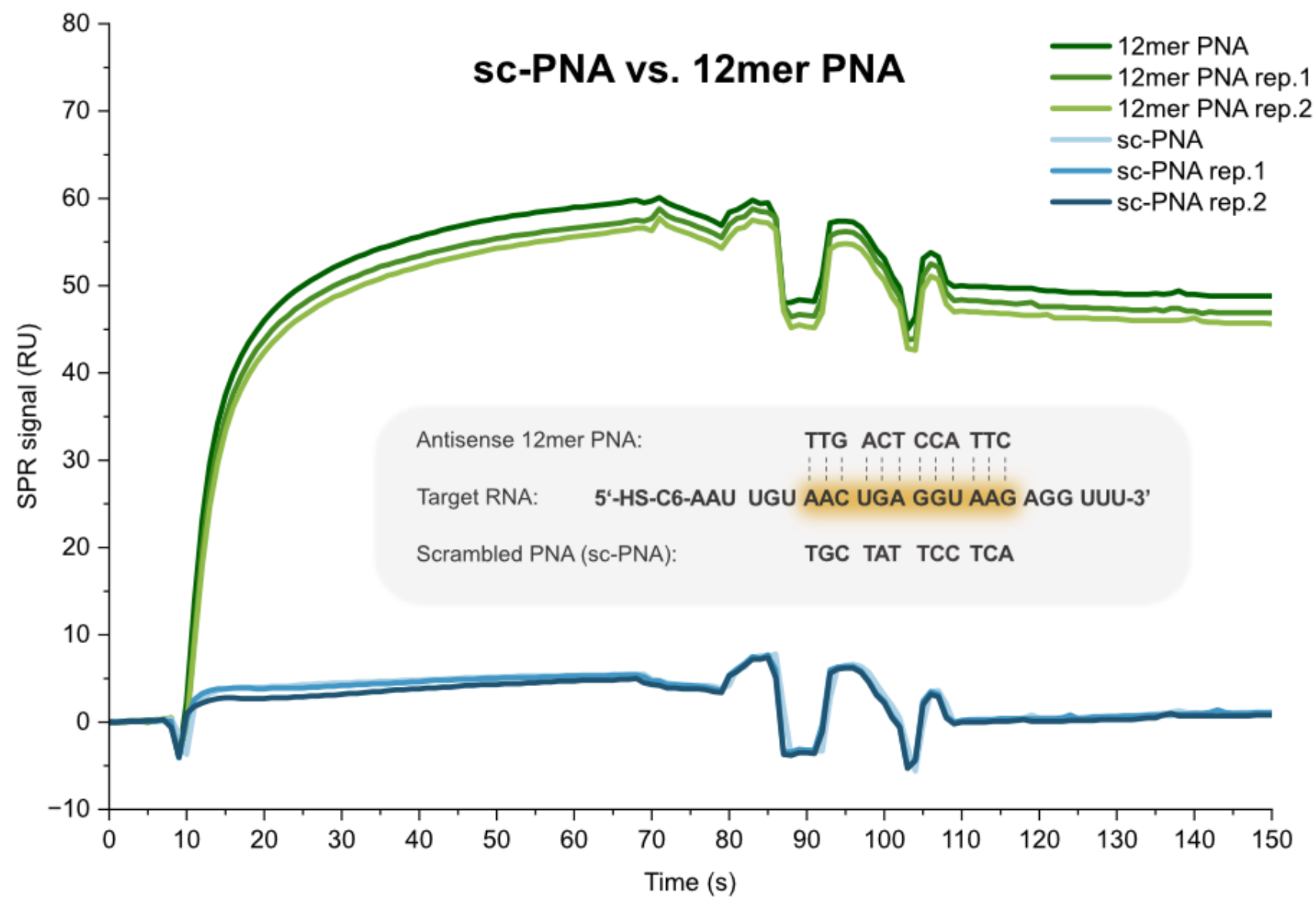

SI Figure 4: Hybridization of scrambled PNA (sc-PNA, blue) vs. complementary 12mer PNA (green) to target RNA. Abbreviations: RU = resonance units.

5.13. Sensorgram of 6mer PNA without lysine modification

Hybridization experiments of 6mer PNA without lysine modification were conducted using the same protocol as described in section 1.3. A representative measurement is shown in SI Figure 5. No clearly detectable concentration-dependent SPR response was observed under these conditions, indicating that hybridization of the unmodified 6mer PNA to the surface-immobilized RNA was below the reliable detection range of the experiment.

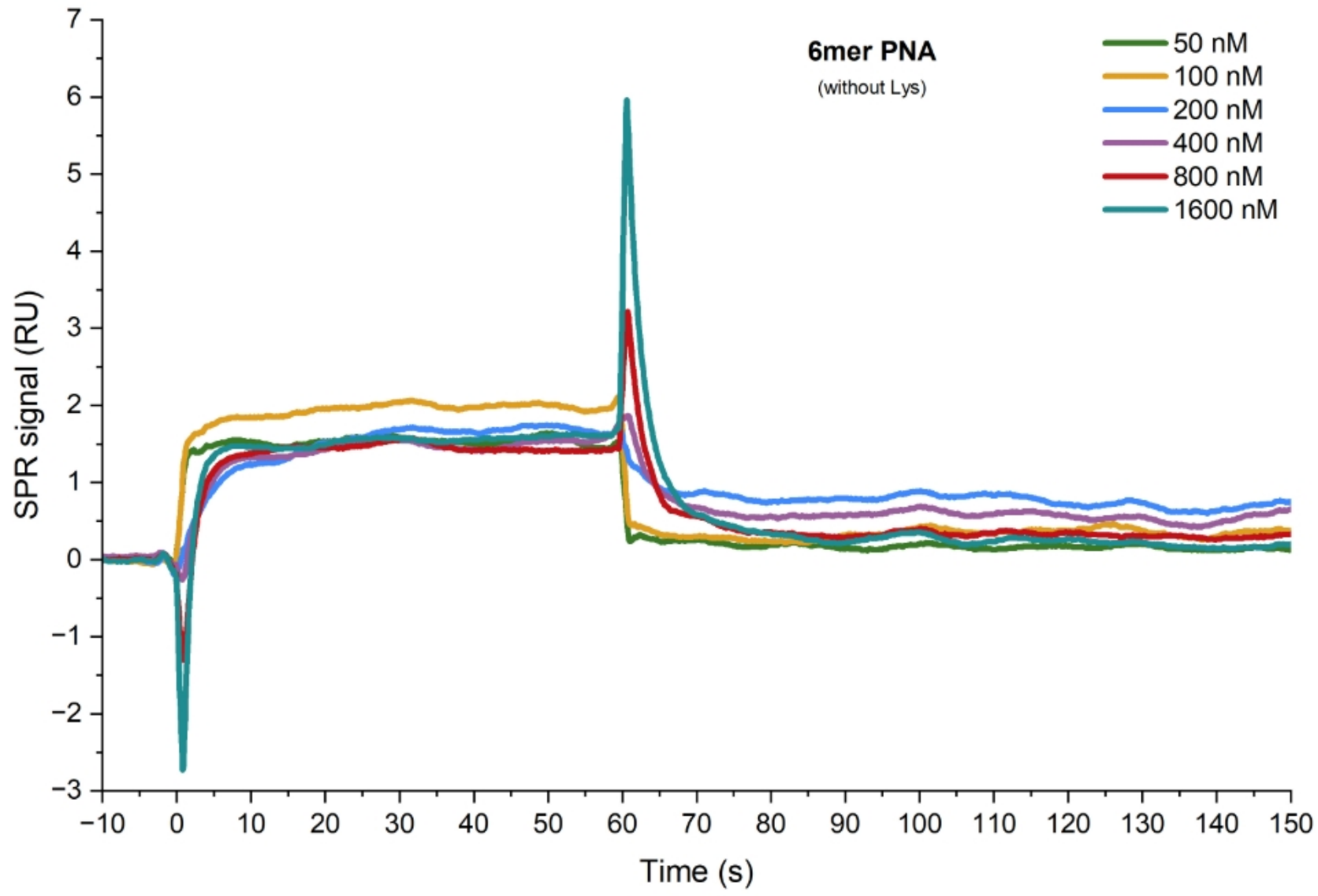

SI Figure 5: Hybridization of 6mer PNA without lysine (Lys) modification to target RNA. Abbreviations: RU = resonance units.

5.14. Sensorgram of 10mer PNA without lysin modification

Hybridization experiments of 10mer PNA without lysine modification were conducted using the same protocol as described in section 1.3. Two representative measurements are shown in SI Figure 6. In these experiments, the expected monotonic increase of SPR signal with increasing analyte concentration was not consistently observed, and therefore, these measurements were not used for quantitative determination of k_a and k_d. Specifically, the order of concentrations based on RU signals deviates from the expected trend. This discrepancy indicates that the concentration-dependent response is not strictly preserved under these conditions.

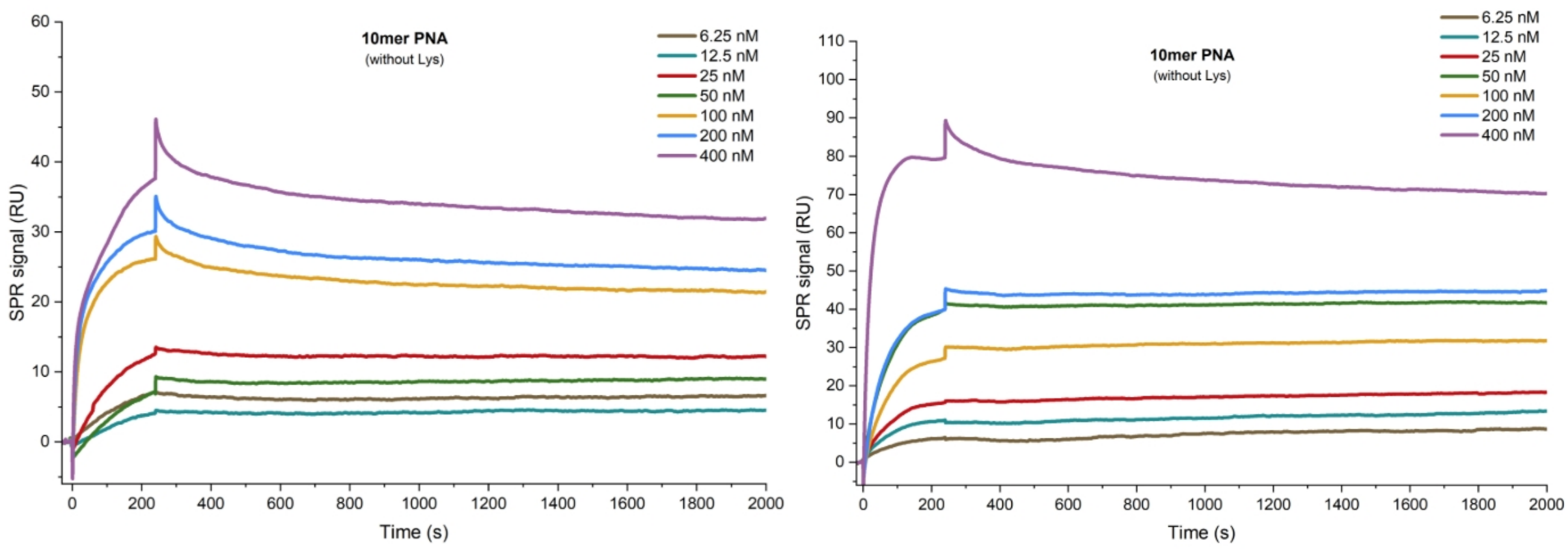

SI Figure 6: Hybridization of 10mer PNA without lysine (Lys) modification to target RNA (two measurements as representatives); Abbreviations: RU = resonance units.

5.15. Control of surface regeneration

To assess the stability of the immobilized RNA surface, the same concentration was measured at the beginning and at the end of a hybridization cycle. The comparable SPR responses indicate that the hybridization capacity of the immobilized RNA was maintained throughout the cycle. One representative measurement is shown in SI Figure 7.

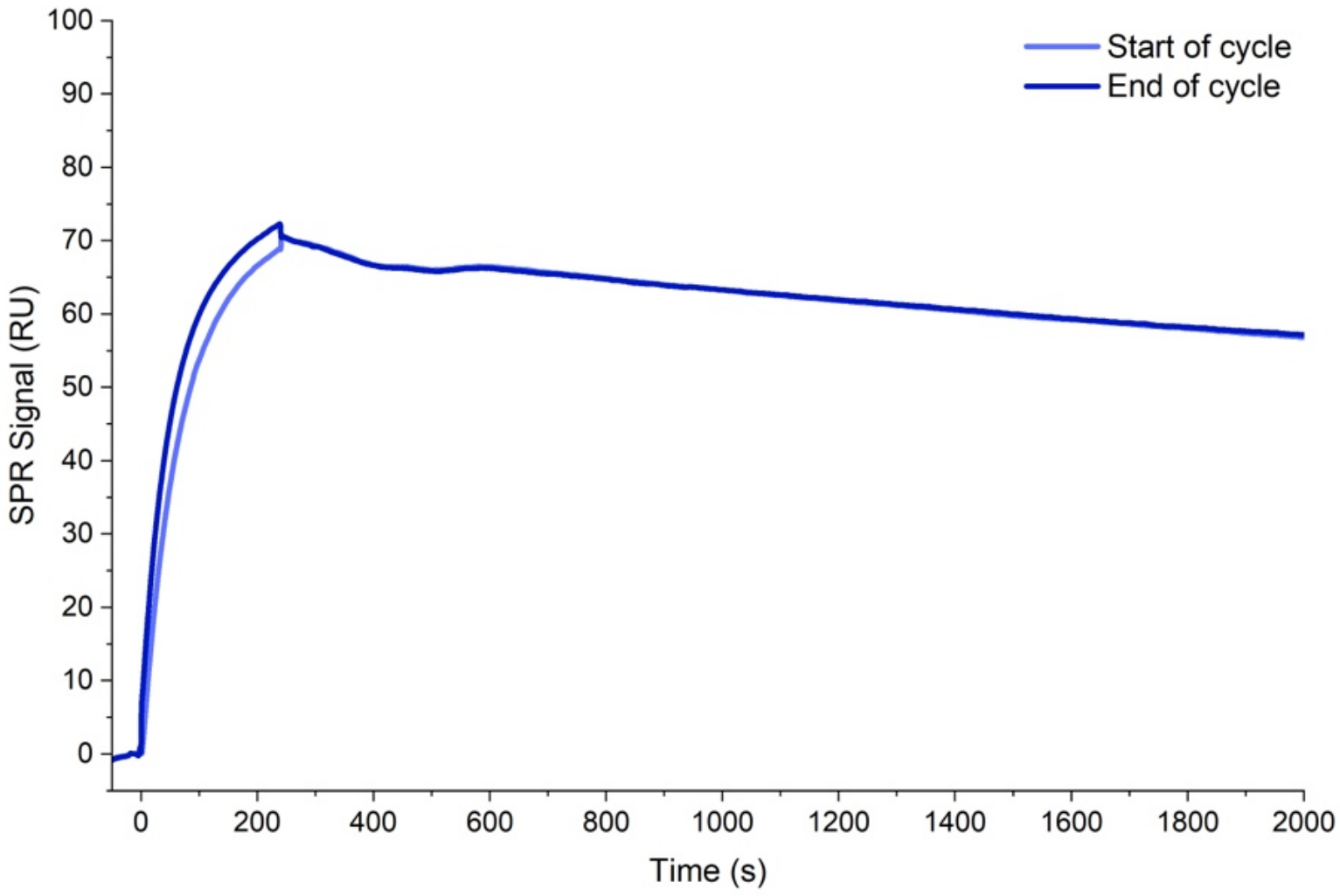

SI Figure 7: Hybridization of 12mer RNA at the start and the end of a hybridization kinetic cycle; Abbreviations: RU = resonance units.

6. References

18. Sahu, B.; Sacui, I.; Rapireddy, S.; Zanotti, K. J.; Bahal, R.; Armitage, B. A.; Ly, D. H., Synthesis and Characterization of Conformationally Preorganized, (R)-Diethylene Glycol-Containing γ-Peptide Nucleic Acids with Superior Hybridization Properties and Water Solubility. *The Journal of Organic Chemistry* **2011,** *76* (14), 5614-5627.

39. Christensen, L.; Fitzpatrick, R.; Gildea, B.; Petersen, K. H.; Hansen, H. F.; Koch, T.; Egholm, M.; Buchardt, O.; Nielsen, P. E.; Coull, J.; Berg, R. H., Solid-phase synthesis of peptide nucleic acids. *Journal of peptide science : an official publication of the European Peptide Society* **1995,** *1* (3), 175-83.
